# Design and synthesis of tricyclic terpenoid derivatives as novel PTP1B inhibitors with improved pharmacological property and *in vivo* antihyperglycaemic efficacy

**DOI:** 10.1080/14756366.2019.1690481

**Published:** 2019-11-19

**Authors:** Lingling Yang, Feng Chen, Cheng Gao, Jiabao Chen, Junyan Li, Siyan Liu, Yuanyuan Zhang, Zhouyu Wang, Shan Qian

**Affiliations:** a Department of Pharmaceutical Engineering, College of Food and Bioengineering, Xihua University, Chengdu, China; b Department of Chemistry, College of Science, Xihua University, Chengdu, China

**Keywords:** Protein tyrosine phosphatase 1B, oleanolic acid, insulin-resistant, antihyperglycaemic effect, type 2 diabetes

## Abstract

Overexpression of protein tyrosine phosphatase 1B (PTP1B) induces insulin resistance in various basic and clinical research. In our previous work, a synthetic oleanolic acid (OA) derivative **C10a** with PTP1B inhibitory activity has been reported. However, **C10a** has some pharmacological defects and cytotoxicity. Herein, a structure-based drug design approach was used based on the structure of **C10a** to elaborate the smaller tricyclic core. A series of tricyclic derivatives were synthesised and the compounds **15**, **28** and **34** exhibited the most PTP1B enzymatic inhibitory potency. In the insulin-resistant human hepatoma HepG2 cells, compound **25** with the moderate PTP1B inhibition and preferable pharmaceutical properties can significantly increase insulin-stimulated glucose uptake and showed the insulin resistance ameliorating effect. Moreover, **25** showed the improved *in vivo* antihyperglycaemic potential in the nicotinamide–streptozotocin-induced T2D. Our study demonstrated that these tricyclic derivatives with improved molecular architectures and antihyperglycaemic activity could be developed in the treatment of T2D.

## Graphical Abstract

**Figure:**
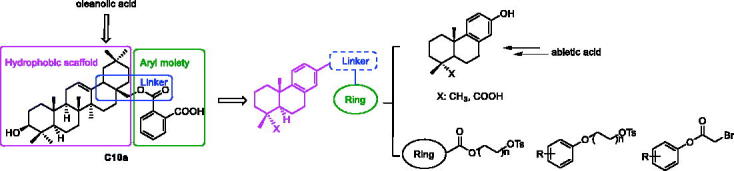

## 1. Introduction

The predominant pathophysiological factor of type 2 diabetes (T2D) is insulin resistance^1–4^. Though many anti-T2D drugs have emerged in the past decade^5^^,^^6^, traditional anti-T2D agents such as repaglinide, metformin, dipeptidyl peptidase-IV inhibitors, α-glucosidase inhibitors and glucagon-like peptide-1 agonists, and thiazolidinediones are still the most efficacious oral drugs in the first-line monotherapy of T2D^7^. It is undoubted that new insulin sensitisers will meet great needs of T2D patients^8^^,^^9^.

Protein tyrosine phosphatase 1B (PTP1B) dephosphosphorylates the tyrosine-phosphorylated insulin receptor (IR) and the downstream insulin receptor substrate (IRS) to down regulate insulin transduction^10–15^. PTP1B inhibitors could potentially improve insulin sensitivity and normalise glucose levels and therefore could be a promising therapeutic strategy in the T2D patients. Recent studies also identified the involvement of intra-islet PTP1B in the regulation of insulin release and reinforce the potential of PTP1B inhibitors for the treatment of beta-cell secretory failure in the pathogenesis of T2D^16^^,^^17^. Besides, PTP1B-mediated dephosphorylation has been implicated in the development of diabetes^18^, cancer^19^, hepatic fibrosis^20^, bacterial infection^21^, rheumatoid arthritis^22^ and hypertension^23^. Many PTP1B inhibitors have been reported, but the discovery of PTP1B inhibitors with superior cell permeability and *in vivo* potency is difficult and so far there is no PTP1B inhibitors entered III phase clinical trial^18^^,^^24^.

**Figure 1.**
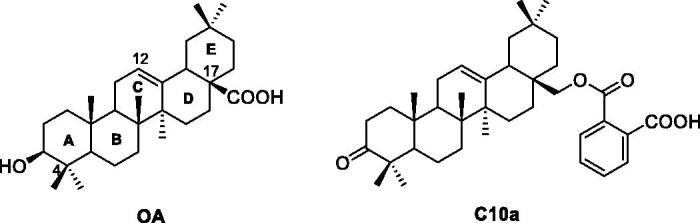
The chemical structures of OA and lead compound **C10a**.

Hundreds of natural products have been isolated and identified as PTP1B inhibitors, and natural products with interesting structural diversity have potential to develop the new PTP1B inhibitors^25–27^. In our previous work, some oleanolic acid (OA) derivatives with modified A-ring, C-ring, and C17 moiety were designed and synthesized^28–33^. Within these OA derivatives, compound **C10a** (Figure 1) exhibited the most PTP1B inhibition (IC_50_: 3.12 μM), which was 7.6-fold more than the parent compound OA^28^. However, the triterpenoid derivative **C10a** has too large molecular weight (>500) and some pharmacological defects, such as weak cell permeability, poor bioavailability and improper lipid/water partition coefficient. **C10a** also showed the considerable cytotoxicity. Therefore, the structure of **C10a** needs to be optimised to develop the potent PTP1B inhibitors with favourable pharmacological properties.

**Figure 2.**
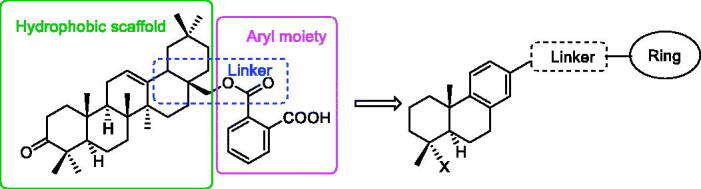
The structural optimisation strategy.

**Figure 3.**
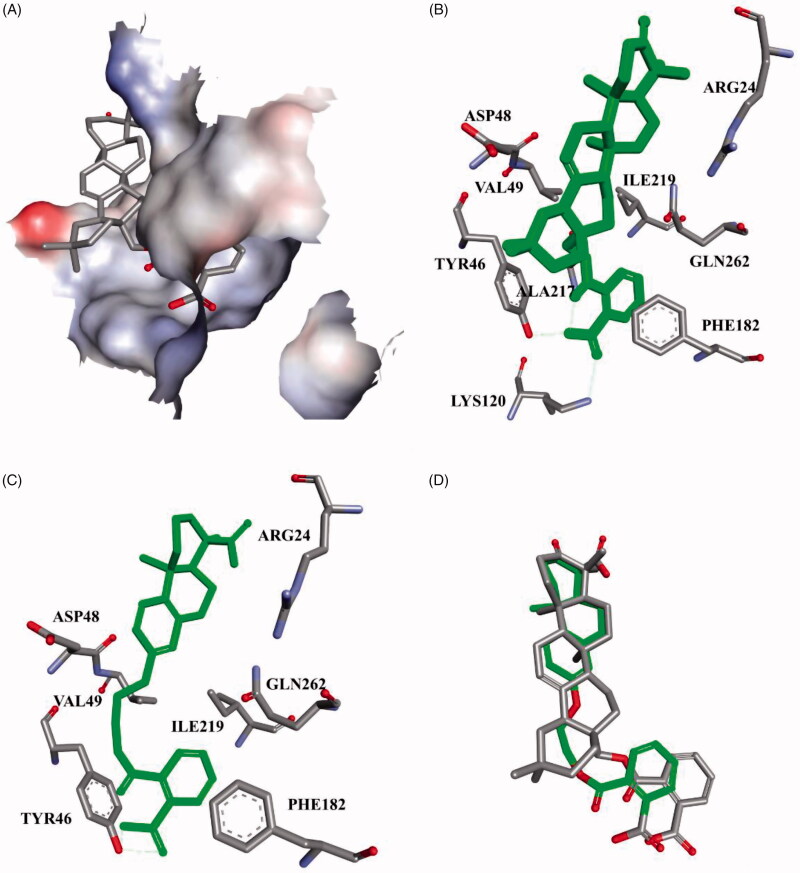
**C10a** and **15** docked in the PTP1B active site (PDB ID: 2B07^34^). (A) Only the active site was shown, displaying the protein in surface representation and ligand **C10a** in stick representation; (B) **C10a**, Coloured green and displayed in stick representation, bound to these important residues in the interior of the active site. All hydrogen atoms are omitted for clarity; (C) **15** bound to the important residues in the interior of the active site; (D) The overlapping docking modes of **C10a** and **15**.

The structural optimisation strategy is shown in Figure 2. The structure of **C10a** contains hydrophobic scaffold, linker and aryl moiety. As displayed in Figure 3(A,B), the molecular docking has demonstrated the hydrophobic interactions between the terpenoid scaffold of **C10a** and the surrounding amino residues of PTP1B are critical for the complex stability, but the pentacyclic core of **C10a** is too complicated. We assumed this scaffold could be simplified to the smaller tricyclic fragment containing the same stereo-conformation of fused A/B ring junction, such as the tricyclic terpenoid scaffold of compound **15** as shown in Figure 3(C). One of the methyl group at 4-position also was retained, because it was beneficial for interaction with Arg24^28^, which is an important residue at the second site of PTP1B for substrate specificity (the second site of PTP1B is a noncatalytic cleft-like binding pocket, which is not conserved among all PTPs)^35^. As shown in Figure 3(C), C ring was replaced with the substituted benzene ring, which could provide opportunities to form more hydrophobic and π–π interactions. D ring and E ring were simplified to the linker from C ring to the aryl moiety. Insertion of polar group (e.g. carboxyl, ether) into this linker would be beneficial for the favourable balance between hydrophilicity and hydrophobicity. The overlapping figure of **C10a** and **15** indicated these compounds have similar docking modes with amino residues of PTP1B (Figure 3(D)). Only two hydrogen-bond interactions between **C10a** and PTP1B were observed (Tyr46 and Lys120), so the aryl moiety of **C10a** was replaced with various substituted rings in order to enhance inhibition, since the aryl moiety was important for the substrate recognition^19^.

## 2. Results and discussion

### 2.1. Chemistry

**Scheme 1.**
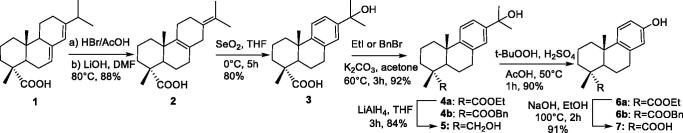
Synthesis of the compounds **2–7**.

The synthesis procedure to achieve 15-hydroxydehydroabietic acid (**3**) from abietic acid (AA) involved addition, elimination, and oxidation. However, according to the literatures^36–38^, alcohol **3** was obtained in only 10% yield in our laboratory. We therefore improved the synthetic method and **3** was finally obtained in 70% overall yield (Scheme 1). According to the improved synthetic procedure, AA (**1**) was treated with 33% HBr/AcOH and the resulting 8, 15-dibromo derivative was heated in the presence of LiOH/DMF to afford diene (**2**), with four methyl groups of all singlets by ^1^HNMR. Oxidative rearrangement of **2** with SeO_2_ provided 15-hydroxydehydroabietate (**3**) in 80% yield. **3** was esterified by treatment with EtI (or BnBr) to give ester **4a** (**4 b**). **4a** was then reduced with LiAlH_4_ to give alcohol **5**. We found 15-hydroxydehydroabietic derivatives are not suitable synthesis intermediates because of high steric hindrance of C-15 position, and thus hydroperoxide rearrangement of esters **4a–b** with *t*-BuOOH and H_2_SO_4_/AcOH gave the 13-hydroxy-8,11,13-podocarpatriene derivatives (**6a–b**)^39^. Finally, ester **6a** was saponified with aq.NaOH to give acid **7**.

**Scheme 2.**
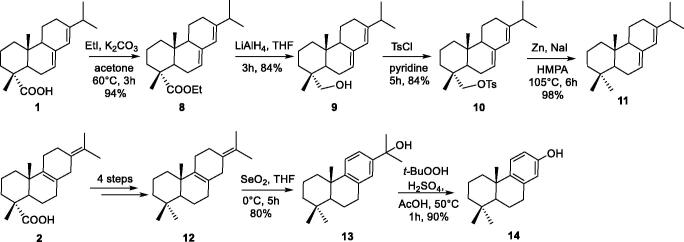
Synthesis of the compounds **8–14**.

A couple of methyl groups at 4-position is most frequently found in the terpenoid scaffold. As shown in Scheme 2, the conversion of the COOH at 4-position to CH_3_ was also investigated. Acids **1** and **2** were firstly esterified by treatment with EtI and K_2_CO_3_ to give corresponding esters, which were subsequently reduced to afford alcohols. Conversion of alcohols to its tosylates in pyridine proceeded in satisfactory yield. Zn/NaI reduction^40^ of abietane-tosylates afforded dienes **11–12** in satisfactory yield, respectively. Then, diene **12** was oxidative rearranged with SeO_2_ to obtain alcohol **13**, which was converted to phenol **14** by hydroperoxide rearrangement.

**Scheme 3.**
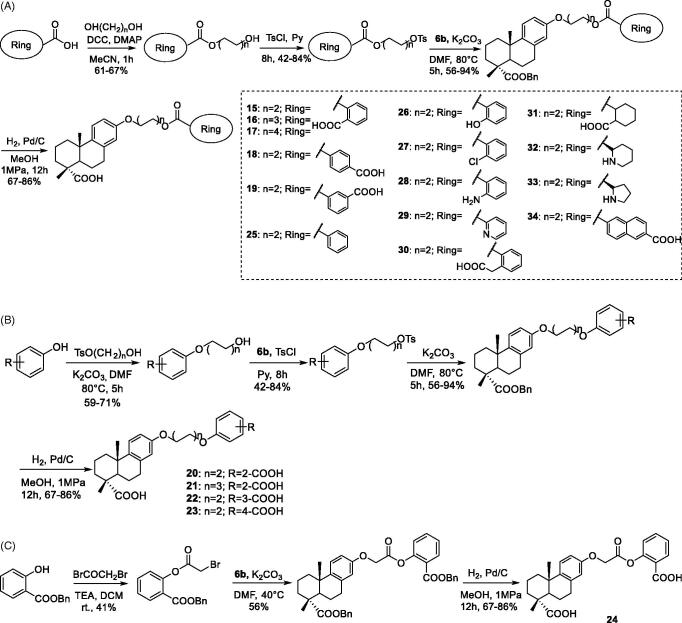
Synthesis of the compounds **15–34**.

The final products **15–34** were synthesised via nucleophilic substitution of **6 b** and corresponding tosylate or alkyl bromide, and subsequent deprotection of O-protected intermediates (Scheme 3(A–C)). As shown in Scheme 3(A), the tosylate intermediates of **15–19** and **25–34** were provided from corresponding alcohols, which were synthesised via condensation of the acids with different rings and diols with different length in the presence of DCC/DMAP. As shown in Scheme 3(B), the tosylate intermediates of **20–23** were provided from corresponding alcohols, which were synthesised via nucleophilic substitution. As shown in Scheme 3(C), the alkyl bromide intermediate of **24** was synthesised from the phenol and bromoacetyl bromide in alkaline condition.

### 2.2. Enzymatic hPTP1B inhibitory activities and SAR analysis

As shown in Table 1, the PTP1B inhibitory activities of abietic derivatives with two types of terpenoid scaffolds (A and B) were firstly evaluated. Structure–activity relationship (SAR) was also discussed. The carboxyl group at 4-position was beneficial for PTP1B inhibition. Esterification of the carboxyl group to give corresponding ethyl ester (**3** vs **4a**), or reduction to the corresponding alcohol (**1** vs **9**, **3** vs **5**) all resulted in loss of activity. Replacing the carboxyl group at 4-position with methyl group resulted in significant drop in activity (**1** vs **11**). Besides, transformation of hydroxy isopropyl group at 13-position of 8,11,13-podocarpatriene derivatives (scaffold B) to hydroxy group (**3** vs **7**, **13** vs **14**) resulted in loss of activity.

**Table 1.** The PTP1B inhibitory activities of abietic derivatives **1–14.**


Cpd.	terpenoid scaffold	X	Y	Inh%@10 μM^a^
**1**	A	COOH	C(CH_3_)_2_	35
**3**	B	COOH	C(CH_3_)_2_OH	19
**4a**	B	COOEt	C(CH_3_)_2_OH	4
**5**	B	CH_2_OH	C(CH_3_)_2_OH	0
**7**	B	COOH	OH	1
**9**	A	CH_2_OH	C(CH_3_)_2_	3
**10**	A	CH_2_OTs	C(CH_3_)_2_	2
**11**	A	CH_3_	C(CH_3_)_2_	0
**13**	B	CH_3_	C(CH_3_)_2_OH	22
**14**	B	CH_3_	OH	15

^a^ These experiments were performed in triplicate.

Among tricyclic derivatives **15**–**34**, the compounds **15** (2.9 μM), **28** (4.8 μM) and **34** (8.2 μM) exhibited the most PTP1B enzymatic inhibitory activities, which were equal to that of the lead compound **C10a** (3.1 μM), and they have smaller molecular weight (Table 2). SAR analysis demonstrated that the derivatives with different linker between terpenoid scaffold and ring moiety displayed different PTP1B inhibition activity. The inhibitory activity of PTP1B decreased along with an increase of the length of linker (**15 > 16 > 17**). Also, the structure of the linker influenced PTP1B inhibition. Compared with **20** and **24**, **15** displayed higher PTP1B inhibitory activity. Besides, the derivatives containing a benzene or naphthalene at the ring moiety (**15**, **18–19**, **25–28** and **34**) showed higher inhibitory activity relative to the analogues bearing the heteroaromatic ring (**29**) or alicyclic ring (**31–33**). At the ring moiety, a clear preference was identified for the polar groups, with an increase in activity for the carboxyl group (**15** and **34**) and amino group (**28**), whereas substitution with carboxylmethyl group (**30**) led to significant loss of activity. The benzene ring with a carboxyl group at the *o*-position (**15**) increased the potency relative to those at the *p*-position (**18**) or *m*-position (**19**). Overall, our data demonstrated these compounds with tricyclic diterpene moiety are potent PTP1B inhibitors.

**Table 2.** The PTP1B inhibitory activities of tricyclic derivatives **15–34.**

					

^a^ These experiments were performed in triplicate.

^b^ SD: standard deviation.

### 2.3. Cellular glucose uptake in insulin-resistant HepG2 cells

**Figure 4.**
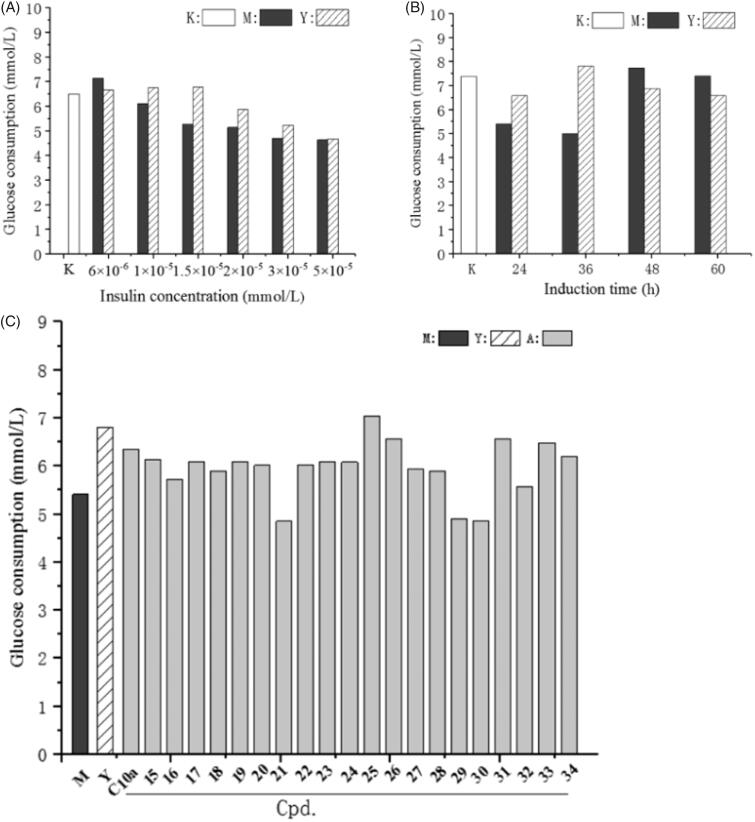
The effect of the compounds on glucose consumption in insulin-resistant HepG2 cells. (A) The HepG2 cells were induced with different concentrations of insulin; (B) The HepG2 cells were induced for different induction times; (C) HepG2 cells were induced with 1.5 × 10^−5 ^mmol of insulin for 36 h to afford insulin-resistant cells, and then treated with rosiglitazone (Y group) or the compounds for 24 h. After incubation, glucose content in the culture medium was measured by glucose oxidase method. M group: insulin-resistant HepG2 cells without rosiglitazone and the compounds; K group: HepG2 cells without induction by insulin.

To assess the insulin sensitisation, the cellular effect of these compounds on insulin-resistant human hepatoma HepG2 cells was performed. The concentration of insulin and induction time were screened to induce insulin resistance in HepG2 cells according to the reported method^41^^,^^42^ with some modification. As shown in Figure 4(A), treatment with above 1 × 10^−5 ^mmol of insulin reduced glucose uptake, whereas treatment with 6 × 10^−6 ^mmol of insulin enhanced glucose uptake. Insulin resistance can be obviously observed when the HepG2 cells were treatment with 1.5 × 10^−5 ^mmol of insulin, and an insulin sensitiser (rosiglitazone) can significantly decrease the insulin resistance and improve the glucose uptake. Treatment with higher concentration (>2 × 10^−5 ^mmol) of insulin inhibited cell growth, and microscopic examination revealed atrophy of the cells. As shown in Figure 4(B), insulin resistance was induced in HepG2 cells at 36 h, and rosiglitazone can decrease the insulin resistance. The cell growth was accelerated after 36 h and insulin resistance was decreased along with induction time. Therefore, the cells were treated with 1.5 × 1 0 ^−5 ^mmol of insulin for 36 h to induce the insulin resistance and produce the most obvious difference of glucose consumption between insulin resistance group (M group) and rosiglitazone group (Y group).

As shown in Figure 4(C), most compounds can enhance the glucose consumption in insulin-resistant HepG2 cells, and the insulin stimulatory effects on glucose uptake were similar to that of **C10a**, but inferior to that of rosiglitazone. Cell viability was evaluated with the MTT assay and the result demonstrated that the cytotoxicities of most compounds were acceptable, while **C10a** showed the considerable cytotoxicity (see Figure 1S, Supplementary material). Compared with **15**, which exhibited the most PTP1B enzymatic inhibitory potency, compound **25** has lower PTP1B inhibition (26.2 μM), but it can more significantly increase insulin-stimulated glucose uptake in cellular assay. This result was unsurprising, because most active site-directed PTP1B inhibitors are phosphotyrosine mimetics with weak cell permeability^43^. We have previously reported that there is a trade-off between enzymatic inhibition and cell permeability^18^. We calculated the pharmaceutical properties of **15**, **25** and **C10a** using the Swiss ADME. As shown in Table 3, the MW and cLog*P* value of **C10a** are too large, as well as the HB acceptors of **15**. Both of these compounds could not fulfil Lipinski Rule^44^. **25** fulfilled Lipinski Rule of Five and has the lowest TPSA, which means more acceptable oral absorption and cell permeability. These results demonstrated *in silico* preferable pharmaceutical properties of **25** were beneficial for its cellular effect.

**Table 3.** Pharmaceutical properties of **15**, **25** and **C10a**.

Cpd.	MW	HB donors	HB acceptors	cLog *P*	Rotatable bonds	TPSA (Å^2^)	GI absorption	P-gp substrate	Cytotoxicity
**15**	466	2	7	4.23	8	110.13	High	Yes	Low
**25**	422	1	5	4.68	7	72.83	High	Yes	Low
**C10a**	588	1	5	7.32	5	80.67	Low	No	High

cLog *P*: consensus log of the octanol/water partition coefficient; GI absorption: gastrointestinal absorption; MW: molecular weight; TPSA: topological polar surface area.

### 2.4. The antihyperglycaemic effect in NIDDM mice

**Figure 5.**
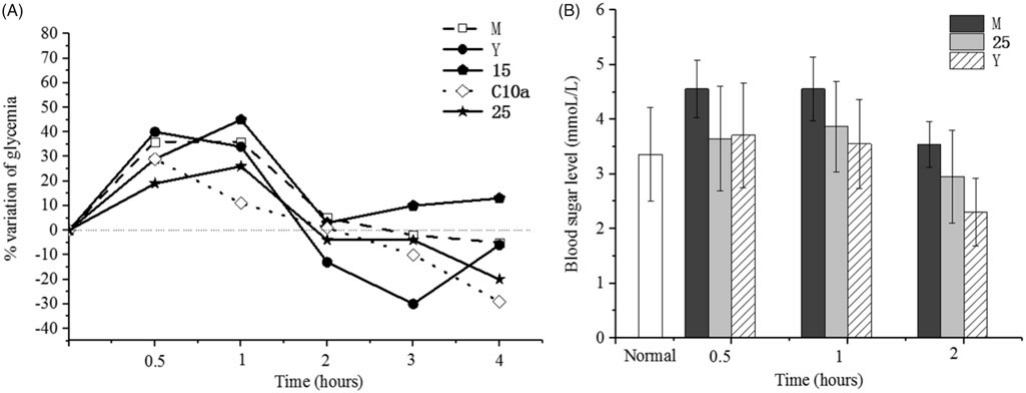
The antihyperglycaemic effect in NIDDM mice (*n* = 8). (A) Diminution of plasmatic glucose concentration over NIDDM mice treated with **15**, **25**, **C10a** and rosiglitazone, respectively; (B) The acute antidiabetic effect of **25** were observed with respect to positive control. M group: NIDDM mice were treated with saline alone. Y group: NIDDM mice were treated with rosiglitazone.

The antihyperglycaemic effect of the compounds **15**, **25** and **C10a** were evaluated on non-insulin dependent diabetes mellitus (NIDDM) mice. Nicotinamide and streptozotocin were administrated intraperitoneally to induce hyperglycaemia and the plasma glucose of mice raised over 4.5 mmol/L^45^. Rosiglitazone has not shown effect during the first hour, but it showed the significant antihyperglycaemic effect after 1 h (Figure 5(A,B)). Though **C10a** showed the antihyperglycaemic effect, some mice treated with **C10a** appeared the hydroabdomen, thrombus and tail necrosis, suggesting the acute toxicity of **C10a**. Compared with **15**, **25** showed *in vivo* antihyperglycaemic effect, correlating with insulin-resistant cell assay.

## 3. Conclusions

In this article, a series of tricyclic derivatives containing various hydrophobic scaffold, linker and aryl moiety were designed and synthesised from 13-hydroxy-8,11,13-podocarpatriene, and their inhibitory activity against the PTP1B enzyme was assessed. Among these compounds, some tricyclic derivatives were described as potent inhibitors of PTP1B. Compound **25** showed the most effect on glucose consumption in insulin-resistant HepG2 cells. Moreover, **25** showed the most antihyperglycemic effect in NIDDM mice. The therapeutic potential of PTP1B inhibition, as well as the insulin sensitising and *in vivo* antihyperglycemic effect of these compounds, made this discovery an important opportunity.

## 4. Experimental section

### 4.1. Chemistry

^1^H NMR spectra were recorded on a Bruker Avance 400 spectrometer. ^13^C NMR spectra were recorded on a Bruker DPX 300 spectrometer. TMS was the internal standard. Chemical shifts as δ values were recorded in ppm and coupling constants as J values were recorded in Hz. HRMS spectra were recorded on a micrOTOF-Q II 10203 spectrometer. TLC were performed on the HSGF254 silica gel and UV detection at 254 nm and 365 nm, and appropriate chromogenic agents.

#### 4.1.1. Synthesis of abieta-8(9),13(15)-dien-18-oic acid (2)

To a solution of **1** (20.00 g, 66.40 mmol) in 64 ml of HOAc was added to 33% HBr/HOAc (64 ml). The mixture was stirred at rt for 6 h, and then was filtered and the filter cake was washed with HOAc, dried in vacuum overnight to give corresponding crude dibromo derivative. To a solution of the crude 8,15-dibromo derivative was added LiOH (2.64 g, 64.72 mmol) in 200 ml of DMF, and the mixture was stirred at 80 °C for 7 h. Solvent was removed under reduced pressure, and the pH was adjusted to 3 with HCl (aq) and concentrated to give a brown oil **2** without further purification.

#### 4.1.2. General procedure 1: SeO_2_-mediated oxidative rearrangement

To a solution of 1 equiv. diene in 10 ml/mmol of dry THF was added 4 equiv. SeO_2_, and the mixture was stirred at 0 °C for 5 h under an atmosphere of Ar. The solid was removed by filtration, and the filtrate was extracted with EtOAc. Solvent was removed under reduced pressure, and the resulting residue was purified by flash column chromatography to give product.

*15-Hydroxydehydroabieta-18-oic acid (****3****).* Yellow oil; mp: 92–93 °C; ^1^H NMR (400 MHz, CDCl_3_) δ 7.24 (m, 2H), 7.18 (s, 1H), 2.96 (m, 1H), 2.43–2.00 (m, 3H), 1.92–1.85 (m, 2H), 1.85–1.77 (m, 2H),1.77–1.70 (s, 3H), 1.58 (s, 6H), 1.46 (s, 3H), 1.31 (s, 3H). ^13^C NMR (300 MHz, CDCl_3_): δ 184.5, 147.8, 145.9, 134.7, 124.9, 124.1, 121.9, 120.2, 72.4, 60.4, 47.3, 44.5, 37.8, 36.9, 36.7, 31.6, 30.1, 25.0, 18.5, 16.2. HRMS (AP-ESI) Calcd. For C_20_H_28_NaO_3_: 339.2038 [M + Na]^+^. Found: 339.2087.

*15-Hydroxydehydroabietane (****13****).* Yellow oil; ^1^H NMR (400 MHz, CDCl_3_) δ 7.80 (d, *J* = 8.2 Hz, 1H), 7.37 (d, *J* = 8.0 Hz, 1H), 7.22 (s, 1H), 5.32 (s, 1H), 3.85 (d, *J* = 9.3 Hz, 1H), 3.62 (d, *J* = 9.4 Hz, 1H), 2.93–2.73 (m, 2H), 2.48 (s, 3H), 1.71 (m, 2H), 1.60 (s, 3H), 1.58 (s, 3H), 1.51–1.30 (m, 6H), 1.20 (s, 3H), 0.91 (s, 3H). ^13^C NMR (300 MHz, CDCl_3_) δ 129.8, 127.9, 124.8, 124.2, 121.9, 77.6, 72.2, 43.5, 38.0, 37.3, 37.1, 35.0, 31.6, 29.9, 21.6, 18.8, 18.3, 17.1. HRMS (AP-ESI) Calcd. for C_20_H_30_NaO: 309.2195 [M + Na]^+^. Found: 309.2185.

#### 4.1.3. General procedure 2: K_2_CO_3_-mediated esterification

To a solution of 1 equiv. abieta-18-oic acid in acetone was added 3 equiv. K_2_CO_3_, and the mixture was stirred for 15 min. The solution was dropwise added 2 equiv. EtI (for **4a** and **8**) or BnBr (for **4 b**) and the mixture was stirred at 60 °C for 3 h. The mixture was extracted with EtOAc. Solvent was removed under reduced pressure, and the resulting residue was purified by flash column chromatography to give product.

*Ethyl 15-hydroxydehydroabietate (****4a****).* Yellow solid; mp: 51–52 °C; ^1^H NMR (400 MHz, CDCl_3_) δ 7.26 (s, 2H), 7.19 (s, 1H), 4.11–4.18 (m, 2H), 3.05–2.61 (m, 2H), 2.44–2.25 (m, 2H), 1.91–1.83 (m, 2H), 1.83–1.70 (m, 3H),1.31–1.27 (m, 5H), 1.26 (s, 3H), 1.24 (s, 3H). ^13^C NMR (300 MHz, CDCl_3_) δ 178.4, 147.8, 145.9, 134.6, 126.8, 124.8, 124.1, 121.9, 72.1, 60.4, 47.3, 44.6, 37.8, 36.9, 36.4, 31.5, 30.2, 29.6, 25.0, 23.9, 21.5, 18.5, 16.4, 14.1. HRMS (AP-ESI) Calcd. for C_22_H_32_KO_3_: 283.1988 [M + K]^+^. Found: 383.1933.

*Benzyl 15-hydroxydehydroabietate (****4 b****).* Yellow solid; mp: 112–113 °C; ^1^HNMR (400 MHz, CDCl_3_) δ 7.42–7.31 (m, 1H), 7.26–7.15 (m, 1H), 7.06–7.00 (m, 1H), 6.89 (s, 1H), 5.19 (dd, *J* = 12.4, 3.4 Hz, 2H), 5.10 (s, 1H), 5.07 (s, 1H), 2.91–2.75 (m, 1H), 2.36–2.25 (m, 1H), 1.86–1.61 (m, 2H), 1.33–1.31 (m, 1H), 1.29 (s, 1H), 1.24 (dd, *J* = 6.4, 3.8 Hz, 1H). ^13^C NMR (300 MHz, CDCl_3_) δ 177.9, 146.9, 145.7, 136.1, 134.7, 128.9, 127.6, 127.1, 124.9, 124.3, 123.0, 73.9, 67.0, 51.8, 43.8, 38.4, 37.6, 31.6, 30.0, 28.3, 24.7, 20.1, 19.7. HRMS (AP-ESI) Calcd. for C_27_H_35_O_3_: 407.2581 [M + H]^+^. Found: 407.2585.

#### 4.1.4. General procedure 3: LiAlH_4_- participated reduction

To a suspension of 4 equiv. LiAlH_4_ in THF was added 1 equiv. ester in 10 ml/mmol of THF at 0 °C. The mixture was stirred at rt for 3 h, and then was quenched by addition of aq.NaOH. The resulting solid was filtered off. Solvent of the filtrate was removed under reduced pressure, and the resulting residue was purified by flash column chromatography to give product.

*1-Hydroxylmethyl-15-hydroxydehydroabietate (****5****)*. Colourless oil; ^1^H NMR (400 MHz, *d_6_*-DMSO) δ 12.17 (br, 1H), 8.99 (br, 1H), 7.03 (d, *J* = 8.6 Hz, 1H), 6.53 (dd, *J* = 8.5, 2.5 Hz, 1H), 6.37 (d, *J* = 2.4 Hz, 1H), 4.12 (br, 1H), 3.17 (d, *J* = 2.0 Hz, 2H), 2.73–2.67 (m, 2H), 2.25 (d, *J* = 12.4 Hz, 1H), 2.00 (dd, *J* = 12.3, 1.6 Hz, 1H), 1.75–1.55 (m, 8H), 1.38–1.24 (m, 4H), 1.15 (s, 3H), 1.09 (s, 3H). ^13^C NMR (300 MHz, *d_6_*-DMSO) δ 179.9, 155.0, 140.5, 135.8, 125.5, 114.9, 113.6, 53.7, 49.0, 46.7, 45.3, 38.4, 36.7, 36.5, 30.0, 25.4, 21.6, 18.6, 16.7, 14.4. HRMS (AP-ESI) Calcd. for C_20_H_31_O_2_: 303.2246 [M + Na]^+^. Found: 303.2221.

*1-Hydroxymethylabietane (****9****).* Yellow solid; mp: 125–126 °C; ^1^HNMR (400 MHz, CDCl_3_) δ 5.81 (s, 1H), 5.49–5.42 (m, 1H), 4.25 (s, 1H), 3.54–3.45 (m, 2H), 2.37–1.92 (m, 6H), 1.91–1.77 (m, 4H), 1.67–1.52 (m, 5H), 1.53–1.40 (m, 4H), 1.34–1.26 (m, 6H), 1.26–1.20 (s, 3H), 1.20–1.15 (m, 6H), 1.09–0.98 (m, 8H), 0.98–0.86 (m, 11H), 0.81 (s, 4H). ^13^C NMR (300 MHz, CDCl_3_) δ 146.0, 145.9, 134.7, 124.9, 124.1, 121.9, 75.7, 72.4, 48.3, 44.5, 37.8, 36.9, 36.7, 31.6, 30.2, 25.0, 21.7, 19.5, 14.2. HRMS (AP-ESI) Calcd. for C_20_H_33_O: 289.2526 [M + Na]^+^. Found: 289.2531.

#### 4.1.5. General procedure 4: t-BuOOH-mediated hydroperoxide rearrangement

To a solution of 1 equiv. ester **5** in 5 ml/mmol AcOH was added 3 equiv. *t*-BuOOH followed by addition of 0.1 equiv. H_2_SO_4_. The mixture was stirred at 50 °C for 1 h. The mixture was diluted with water and the aqueous phase was extracted with DCM. The organic phase was washed with Na_2_CO_3_ (aq), water and dried. Solvent was removed under reduced pressure, and the resulting residue was purified by flash column chromatography to furnish the product.

*Ethyl 13-hydroxy-8,11,13-podocarpatriene-1-carboxylate (****6a****).* White solid; mp: 96–97 °C; ^1^H NMR (400 MHz, CDCl_3_) δ 7.12 (d, *J* = 8.6 Hz, 1H), 6.66 (dd, *J* = 8.5, 2.6 Hz, 1H), 6.53 (d, *J* = 2.4 Hz, 1H), 5.25 (br, 1H), 4.35–3.96 (m, 2H), 2.85 (dd, *J* = 10.8, 5.2 Hz, 2H), 2.44–2.17 (m, 2H), 1.90–1.62 (m, 7H), 1.29–1.24 (m, 6H), 1.21 (s, 3H). ^13^C NMR (300 MHz, CDCl_3_) δ 178.8, 153.2, 142.0, 136.6, 125.5, 114.8, 113.1, 60.6, 47.4, 44.9, 38.1, 36.5, 30.1, 25.2, 23.6, 21.5, 18.6, 16.4, 14.2. ESI-MS: 303.1 [M + H]^+^.

*Benzyl 13-hydroxy-8,11,13-podocarpatriene-1-carboxylate (****6 b****).* White solid; mp: 102–103 °C; ^1^H NMR (400 MHz, CDCl_3_) δ 9.26 (s, H),7.44–7.31 (m, 6H), 7.27–7.16 (m, 2H), 2.88–2.78 (m, 2H), 2.35–2.20 (m, 2H), 1.85 –1.67 (m, 2H), 1.58 (s, 3H), 1.23 (d, *J* = 3.3 Hz, 3H). ^13^C NMR (300 MHz, CDCl_3_) δ 178.5, 153.1, 142.0, 136.6, 136.3, 128.5, 128.1, 128.1, 125.6, 114.9, 113.1, 66.4, 47.7, 45.0, 38.1, 36.4, 36.5, 30.0, 25.3, 21.5, 18.6, 16.6. HRMS (AP-ESI) Calcd. for C_24_H_29_O_3_: 365.2111 [M + H]^+^. Found: 365.2119.

*13-Hydroxy-8,11,13-podocarpatriene (****14****).* Yellow solid. mp: 145–146 °C; ^1^H NMR (400 MHz, CDCl_3_) δ 7.14 (d, *J* = 8.6 Hz, 1H), 6.64 (dd, *J* = 8.5, 2.7 Hz, 1H), 6.53 (d, *J* = 2.6 Hz, 1H), 4.77 (s, 1H), 3.02–2.65 (m, 2H), 1.88 (m, 1H)1.81–1.56 (m, 4H), 1.54–1.33 (m, 2H), 1.33–1.23 (m, 2H),1.19 (s, 3H), 0.97 (s, 3H), 0.95 (s, 3H). ^13^C NMR (300 MHz, CDCl_3_) δ 148.1, 138.1, 132.2, 120.9, 110.1, 108.2, 45.8, 37.0, 34.3, 32.6, 28.7, 28.6, 25.7, 20.2, 16.9, 14.6, 14.3. HRMS (AP-ESI) Calcd. for C_17_H_25_O: 245.1878 [M + H]^+^. Found: 245.1895.

#### 4.1.6. Synthesis of 13-hydroxy-8,11,13-podocarpatriene-1-carboxylic acid (7)

To a solution of **6** (0.10 g, 0.33 mmol) in 4 ml EtOH/H_2_O (1:1) was added NaOH (13.1 mg, 0.99 mmol), and the mixture was stirred at 100 °C for 2 h. The mixture was cooled to rt and pH was adjusted to 6 with HCl(aq). The resulting solid was filtered off to give the product **7** (82.5 mg, 91%) as a white solid. mp: 101–102 °C; ^1^H NMR (400 MHz, *d*_6_-DMSO) δ 12.17 (br, 1H), 8.99 (br, 1H), 7.03 (d, *J* = 8.6 Hz, 1H), 6.51 (dd, *J* = 8.5, 2.5 Hz, 1H), 6.38 (d, *J* = 2.4 Hz, 1H), 2.82–2.60 (m, 1H), 2.25 (d, *J* = 12.4 Hz, 1H), 2.00 (dd, *J* = 12.3, 1.6 Hz, 1H), 1.78–1.67 (m, 2H), 1.67–1.56 (m, 2H), 1.56–1.45 (m, 2H), 1.44–1.18 (m, 2H), 1.16 (s, 3H), 1.10 (s, 3H). ^13^C NMR (300 MHz, *d*_6_-DMSO) δ 180.0, 155.1, 140.5, 135.9, 125.5, 115.0, 113.6, 46.8, 45.4, 40.6, 39.3, 36.5, 30.1, 25.5, 21.6, 18.7, 16.8. HRMS (AP-ESI) Calcd. for C_17_H_22_NaO_3_: 297.1467 [M + Na]^+^. Found: 297.1461.

#### 4.1.7. General procedure 5: TsCl-participated sulfonylation

The solution of 1 equiv. alcohol and 5 equiv. tolylsulfonyl chloride in 3 ml/mmol of pyridine was stirred at rt for 6 h. After removal of solvent by evaporation, the mixture was dissolved in DCM, followed by washed and dried. The mixture was then concentrated and subjected to flash column chromatography to furnish the product.

*1-Hydroxymethyl-abietane-p-methylbenzenesulfonate (****10****)*. White solid; mp:67–68 °C; ^1^H NMR (400 MHz, CDCl_3_) δ 7.74–7.66 (d, 2H), 7.40–7.51 (d, 2H),5.81 (s, 1H), 5.58–5.34 (m, 1H), 2.53 (s, 3H),2.32–1.94 (m, 4H), 1.93–1.77 (m, 2H), 1.67–1.54 (m, 3H), 1.50–1.42 (m, 1H), 1.34–1.16 (m, 6H), 1.03 (dd, *J* = 6.8, 4.0 Hz, 6H), 0.94 (q, *J* = 6.5 Hz, 3H), 0.92–0.84 (m, 3H). ^13^C NMR (300 MHz, CDCl_3_) δ 146.0, 144.4, 140.4, 133.5, 130.5, 128.3, 122.4, 121.3, 73.1, 51.7, 51.1, 39.5, 37.0, 35.4, 34.9, 27.7, 24.2, 22.7, 21.0, 20.3, 18.4, 14.7. HRMS (AP-ESI) Calcd. for C_27_H_39_O_3_S: 443.2614 [M + H]^+^. Found: 443.2619.

#### 4.1.8. General procedure 6: Zn/NaI-mediated reduction

The solution of 1 equiv. *p*-toluenesulfonate, 10 equiv. zinc and 5 equiv. sodium iodide in 10 ml/mmol of HMPA was stirred at 105 °C for 7 h. The mixture was dissolved in water and the aqueous layer was extracted with EtOAc. The combined organic layers were washed with brine and dried. Solvent was removed under reduced pressure, and the resulting residue was purified by flash column chromatography to furnish the product.

*7-Isopropyl-1,1,4-trimethyl-decahydrophenanthrene (****11****)*. White oil*;*^1^H NMR (400 MHz, CDCl_3_) δ 5.81 (s, 1H), 5.58–5.34 (m, 1H), 2.32–1.94 (m, 2H), 1.93–1.77 (m, 2H), 1.67–1.54 (m, 3H), 1.50–1.42 (m, 1H), 1.34–1.16 (m, 6H), 1.03 (dd, *J* = 6.8, 4.0 Hz, 6H), 0.94 (q, *J* = 6.5 Hz, 3H), 0.92–0.84 (m, 3H), 0.81 (s, 3H). ^13^C NMR (300 MHz, *d_6_*-DMSO) δ 142.6, 137.5, 123.5, 120.6, 52.2, 50.4, 41.8, 40.4, 39.6, 35.05, 33.5, 31.6, 22.7, 20.8, 19.1, 18.4, 15.2, 14.3. HRMS (AP-ESI) Calcd. for C_20_H_31_: 271.2426 [M – H]^−^. Found: 271.2242.

#### 4.1.9. General procedure 7: DCC-promoted condensation

To a solution of 1 equiv. carboxylic acid in 1.5 equiv. diol was added to a solution of 1.5 equiv. DCC and 0.05 equiv. DMAP in 5 ml/mmol of acetonitrile. The mixture was stirred at 0 °C for 1 h, then transferred to rt and stirred for 5 h. The mixture was extracted with EtOAc. The combined organic layers were washed with brine and dried. Solvent was removed under reduced pressure, and the resulting residue was purified by flash column chromatography to furnish the product.

#### 4.1.10. General procedure 8: K_2_CO_3_-mediated nucleophilic substitution

A solution of 3 equiv. ester in 2 ml/mmol of DMF was added to a solution of 1 equiv. **6b** and 5 equiv. K_2_CO_3_ in 2 ml/mmol of DMF. The mixture was stirred at 80 °C for 5 h. The mixture was extracted with EtOAc. The combined organic layers were washed with brine and dried. Solvent was removed under reduced pressure, and the resulting residue was purified by flash column chromatography to furnish the product.

#### 4.1.11. General procedure 9: Pd/C-catalyzed hydrogenolysis of the O-protected group

Pd/C (10%, 1 mg/mg) was added to the solution of 1 equiv. benzyl ester in 10 ml/mmol of methanol. The mixture was hydrogenolised under 1 MPa at rt for 12 h. The reaction mixture was filtered. Solvent of the filtrate was removed under reduced pressure, and the resulting residue was purified by flash column chromatography to furnish the product.

*Compound* ***15***. White solid; mp: 72–73 °C; ^1^HNMR (400 MHz, CDCl_3_) δ 7.91 (d, *J* = 7.0 Hz, 1H), 7.67 (d, *J* = 7.1 Hz, 1H), 7.63–7.44 (m, 2H), 7.12 (t, *J* = 8.8 Hz, 1H), 6.73 (dd, *J* = 8.5, 1.9 Hz, 1H), 6.58 (s, 1H), 4.64 (s, 2H), 4.23 (s, 1H), 4.15 (q, *J* = 7.1 Hz, 1H), 2.84 (d, *J* = 5.4 Hz, 2H), 2.35–2.11 (m, 2H), 2.08 (s, 1H), 1.92–1.65 (m, 6H), 1.29 (t, *J* = 7.1 Hz, 5H), 1.19 (d, *J* = 7.4 Hz, 3H). ^13^C NMR (300 MHz, CDCl_3_) δ 185.4, 179.8, 173.7, 171.3, 156.1, 142.4, 136.3, 130.9, 129.9, 128.8, 125.5, 125.4, 115.0, 114.1, 113.1, 112.9, 65.5, 60.5, 47.4, 44.7, 38.0, 36.6, 30.2, 25.2, 21.7, 18.5, 16.2, 14.2. HRMS (AP-ESI) Calcd. for C_27_H_29_O_7_: 465.1919 [M – H]^−^. Found: 465.1921.

*Compound* ***16***. White solid; mp: 109–110 °C; ^1^HNMR (400 MHz, CDCl_3_) δ 7.92 (d, *J* = 6.8 Hz, 1H), 7.70 (d, *J* = 7.1 Hz, 1H), 7.59 (dt, *J* = 13.2, 7.2 Hz, 2H), 7.14 (d, *J* = 8.7 Hz, 1H), 6.71 (dd, *J* = 8.6, 2.8 Hz, 1H), 6.56 (dd, *J* = 11.1, 2.6 Hz, 1H), 5.32 (s, 2H), 4.64 (dd, *J* = 5.8, 2.8 Hz, 1H), 4.52 (t, *J* = 6.3 Hz, 1H), 4.29 (d, *J* = 5.2 Hz, 1H), 4.06 (t, *J* = 6.1 Hz, 1H), 4.00 (t, *J* = 6.2 Hz, 1H), 2.86 (s, 3H), 2.25 (dd, *J* = 30.0, 10.2 Hz, 4H), 2.11–2.02 (m, 1H), 1.91–1.78 (m, 4H), 1.78–1.68 (m, 3H), 1.35–1.24 (m, 5H), 1.20 (s, 3H).13C NMR (300 MHz, CDCl_3_) δ 185.6, 172.5, 168.2, 142.0, 136.3, 133.5, 132.3, 130.8, 129.9, 129.9, 128.7, 125.3, 113.9, 112.7, 64.2, 63.0, 47.5, 44.7, 38.0, 36.7, 36.6, 30.2, 28.4, 25.2, 21.7, 18.5, 16.2. HRMS (AP-ESI) Calcd. for C_28_H_31_O_7_: 479.2075 [M – H]^−^. Found: 479.2070.

*Compound* ***17***. White solid; mp: 113–114 °C; ^1^HNMR (400 MHz, CDCl_3_) δ 7.69 (d, *J* = 6.2 Hz, 1H), 7.57 (d, *J* = 4.8 Hz, 2H), 7.12 (t, *J* = 9.5 Hz, 2H), 6.67 (dd, *J* = 21.8, 7.4 Hz, 2H), 6.52 (d, *J* = 19.8 Hz, 2H), 4.40 (s, 1H), 3.95 (s, 1H), 2.84 (s, 3H), 2.25 (dd, *J* = 26.4, 12.1 Hz, 4H), 1.97–1.67 (m, 12H), 1.29 (s, 6H), 1.20 (s, 6H). ^13^C NMR (300 MHz, CDCl_3_) δ 156.4, 141.9, 136.6, 136.3, 131.9, 130.9, 128.7, 125.5, 125.3, 115.0, 113.9, 113.1, 112.6, 67.1, 65.7, 47.4, 44.8, 38.1, 36.8, 36.6, 30.0, 25.2, 25.2, 21.7, 18.5, 16.2. HRMS (AP-ESI) Calcd. for C_28_H_33_O_7_: 493.2232 [M – H]^−^. Found: 493.2226.

*Compound* ***18***. White solid; mp: 112–113 °C; ^1^HNMR (400 MHz, CDCl_3_) δ 8.12 (dt, *J* = 21.5, 10.8 Hz, 1H), 7.13 (d, *J* = 8.6 Hz, 1H), 6.65 (dd, *J* = 8.6, 2.4 Hz, 1H), 6.52 (d, *J* = 2.4 Hz, 1H), 5.33 (s, 1H), 4.70 (dd, *J* = 8.9, 4.3 Hz, 1H), 4.33 (t, *J* = 4.3 Hz, 1H), 2.96–2.79 (m, 2H), 2.34–2.17 (m, 2H), 1.93–1.80 (m, 3H), 1.80–1.70 (m, 2H), 1.54 (q, *J* = 6.9 Hz, 2H), 1.31 (s, 3H), 1.22 (d, *J* = 6.7 Hz, 3H). ^13^C NMR (300 MHz, CDCl_3_) δ 185.0, 165.7, 156.1, 153.1, 142.0, 136.6, 130.1, 129.8, 125.5, 115.0, 114.4, 113.1, 112.7, 65.8, 64.2, 47.4, 44.7, 38.1, 36.7, 36.6, 30.0, 25.2, 21.7, 18.5, 16.2. HRMS (AP-ESI) Calcd. for C_27_H_29_O_7_: 465.1919 [M – H]^−^. Found: 465.1928.

*Compound* ***19***. White solid; mp: 113–114 °C; ^1^HNMR (400 MHz, *d_6_*-DMSO) δ 8.50 (s, 1H), 8.20 (d, *J* = 7.6 Hz, 1H), 8.14 (d, *J* = 7.6 Hz, 1H), 7.65 (t, *J* = 7.7 Hz, 1H), 7.16 (d, *J* = 8.7 Hz, 1H), 6.75 (dd, *J* = 8.6, 2.1 Hz, 1H), 6.63 (s, 1H), 4.62 (s, 2H), 4.30 (s, 2H), 2.87–2.66 (m, 3H), 2.28 (d, *J* = 12.4 Hz, 1H), 2.01 (d, *J* = 11.7 Hz, 1H), 1.45–1.19 (m, 4H), 1.16 (s, 3H), 1.1 (s, 3H). ^13 ^C NMR (300 MHz, CDCl_3_) δ 183.0, 169.7, 165.1, 153.1, 142.0, 136.6, 130.1, 129.8, 125.5, 118.0, 117.4, 116.1, 115.7, 65.8, 64.2, 47.4, 45.7, 38.1, 36.7, 36.6, 30.0, 29.2, 28.7, 24.7, 19.7. HRMS (AP-ESI) Calcd. for C_27_H_29_O_7_: 465.1919 [M – H]^−^. Found: 465.1926.

*Compound* ***20***. White solid; mp: 102–103 °C; ^1^HNMR (400 MHz, CDCl_3_) δ 8.21 (dd, *J* = 7.8, 1.4 Hz, 1H), 7.63–7.51 (m, 1H), 7.21–7.03 (m, 3H), 6.78 (dd, *J* = 8.6, 2.5 Hz, 1H), 6.63 (d, *J* = 2.3 Hz, 1H), 4.63–4.51 (m, 2H), 4.42–4.30 (m, 2H), 3.01–2.78 (m, 3H), 2.27 (dd, *J* = 31.5, 12.4 Hz, 3H), 1.95–1.81 (m, 4H), 1.81–1.70 (s, 3H), 1.28 (d, *J* = 13.2 Hz, 4H), 1.24–1.16 (m, 4H). ^13 ^C NMR (300 MHz, CDCl_3_) δ 184.8, 166.0, 157.3, 155.5, 143.0, 136.6, 134.9, 133.9, 125.6, 122.7, 118.5, 114.2, 113.4, 112.7, 68.5, 65.3, 47.4, 44.7, 38.0, 36.7, 30.2, 25.2, 21.6, 18.5, 16.2. HRMS: m/z calcd for C_26_H_31_O_6_ [M + H]^+^ 439.2115, found 439.2123.

*Compound* ***21***. White solid; mp: 112–113 °C; ^1^HNMR (400 MHz, CDCl_3_) δ 8.19 (dd, *J* = 7.8, 1.7 Hz, 1H), 7.62–7.52 (m, 1H), 7.20–7.03 (m, 3H), 6.77 (dd, *J* = 8.7, 2.6 Hz, 1H), 6.61 (d, *J* = 2.5 Hz, 1H), 5.32 (s, 1H), 4.44 (t, *J* = 6.0 Hz, 2H), 4.17 (t, *J* = 5.4 Hz, 2H), 2.97–2.84 (m, 3H), 2.43–2.34 (m, 2H), 2.30 (d, *J* = 12.6 Hz, 1H), 2.23 (d, *J* = 12.3 Hz, 1H), 1.92–1.80 (m, 4H), 1.80–1.69 (m, 3H),1.54–1.28 (m, 3H),1.25 (s, 3H),1.25 (s, 3H). ^13 ^C NMR (300 MHz, CDCl_3_) δ 184.9, 166.3, 157.7, 156.1, 142.4, 136.4, 135.0, 133.8, 125.5, 122.0, 117.8, 113.8, 112.7, 112.4, 67.5, 64.5, 47.4, 44.7, 38.1, 36.7, 36.6, 30.2, 29.1, 25.2, 21.6, 18.5, 16.2. HRMS: m/z calcd for C_27_H_33_O_6_ [M + H]^+^ 453.2272, found 453.2286.

*Compound* ***22***. White solid; mp: 156–157 °C; ^1^HNMR (400 MHz, CDCl_3_) δ 7.96–7.90 (m, 1H), 7.76–7.68 (m, 1H), 7.60 (tt, *J* = 13.6, 6.8 Hz, 2H), 5.27 (dd, *J* = 25.2, 11.3 Hz, 2H), 4.39 (d, *J* = 11.0 Hz, 1H), 3.92 (d, *J* = 11.1 Hz, 1H), 2.96 (d, *J* = 29.7 Hz, 1H), 2.64–2.50 (m, 1H), 2.40 (dd, *J* = 6.5, 3.5 Hz, 1H), 2.36 (dd, *J* = 6.4, 3.5 Hz, 1H), 2.15–2.07 (m, 1H), 2.02–1.87 (m, 6H), 1.77 (dt, *J* = 13.0, 8.3 Hz, 3H), 1.69–1.60 (m, 2H), 1.45 (s, 4H), 1.29 (t, *J* = 12.0 Hz, 5H), 1.23–1.15 (m, 6H). ^13 ^C NMR (300 MHz, CDCl_3_) δ 185.2, 171.8, 158.7, 156.1, 142.5, 136.5, 130.6, 129.6, 125.4, 123.0, 121.2, 115.2, 114.3, 112.7, 66.8, 66.3, 53.4, 47.4, 44.7, 38.1, 36.6, 30.2, 25.2, 21.7, 18.5, 16.2. HRMS: m/z calcd for C_26_H_31_O_6_ [M + H]^+^ 439.2115, found 439.2120.

*Compound* ***23***. White solid; mp: 158–159 °C; ^1^HNMR (400 MHz, CDCl_3_) δ 7.74 (d, *J* = 7.6 Hz, 1H), 7.68 (s, 1H), 7.40 (t, *J* = 7.9 Hz, 1H), 7.25–7.16 (m, 2H), 6.82–6.76 (m, 1H), 6.65 (s, 1H), 5.33 (s, 1H), 4.36 (dd, *J* = 13.3, 3.8 Hz, 3H), 3.02–2.83 (m, 2H), 2.29 (dd, *J* = 23.2, 12.4 Hz, 2H), 1.87 (dd, *J* = 21.6, 11.8 Hz, 2H), 1.77 (t, *J* = 11.0 Hz, 2H), 1.57 (dd, *J* = 12.7, 6.6 Hz, 1H), 1.48–1.32 (s, 3H), 1.32–1.25 (s, 3H). ^13 ^C NMR (300 MHz, CDCl_3_) δ 185.2, 171.8, 158.7, 156.1, 142.5, 136.5, 130.6, 129.6, 125.4, 123.0, 121.2, 115.2, 114.3, 112.7, 66.8, 66.3, 47.4, 44.7, 38.1, 36.7, 30.2, 25.2, 21.7, 18.5, 16.2. HRMS: m/z calcd for C_26_H_31_O_6_ [M + H]^+^ 439.2115, found 439.2118.

*Compound* ***24***. Yellow oil; mp: 145–146 °C; ^1^H NMR (400 MHz, CDCl_3_) δ 8.21 (dd*, J* = 7.8, 1.8 Hz, 1H), 7.67–7.56 (m, 1H), 7.28 (t*, J* = 4.4 Hz, 1H), 7.22 (t, *J* = 7.2 Hz, 1H), 7.04 (d, *J* = 8.3 Hz, 1H), 6.92 (dd, *J* = 8.6, 2.5 Hz, 1H), 6.83 (d, *J* = 2.5 Hz, 1H), 5.06 (s, 2H), 1.85–1.76 (m, 2H),1.76–1.68 (m, 2H),1.68–1.62 (m, 2H), 1.66–1.40 (m, 3H), 1.31 (s, 3H), 1.23 (s, 3H). ^13 ^C NMR (300 MHz, CDCl_3_) δ 184.6, 166.5, 166.1, 156.5, 147.8, 147.3, 137.0, 134.9, 134.1, 125.7, 123.1, 120.9, 119.0, 118.2, 113.1, 66.4, 47.30, 44.3, 37.9, 37.1, 36.7, 29.9, 25.1, 21.4, 18.4, 16.2. HRMS: m/z calcd for C_26_H_28_O_7_ [M + H]^+^ 454.1908, found 454.1902.

*Compound* ***25***. White solid; mp: 138–139 °C; ^1^H NMR (400 MHz, CDCl_3_) δ 8.13–8.05 (m, 1H), 7.59 (t, *J* = 7.4 Hz, 1H), 7.46 (t, *J* = 7.7 Hz, 1H), 7.19 (d, *J* = 8.7 Hz, 1H), 6.78 (dd, *J* = 8.7, 2.6 Hz, 1H), 6.64 (d, *J* = 2.5 Hz, 1H), 4.67 (t, *J* = 9.2 Hz, 1H), 4.30 (t, *J* = 9.6 Hz, 1H), 3.04–2.83 (m, 2H), 2.39–2.14 (m, 2H), 1.95–1.69 (m, 5H), 1.65–1.48 (m, 2H), 1.31 (s, 3H), 1.23 (s, 3H). ^13 ^C NMR (300 MHz, CDCl_3_) δ 185.0, 166.6, 156.2, 142.4, 136.5, 133.1, 129.9, 129.8, 128.4, 125.4, 125.4, 114.2, 112.7, 65.9, 63.6, 47.4, 44.7, 38.1, 36.7, 30.2, 26.9, 25.2, 21.7, 18.5, 16.2. HRMS: m/z calcd for C_26_H_30_O_5_ [M + H]^+^ 424.2166, found 224.2199.

*Compound* ***26***. White solid; mp: 111–112 °C; ^1^H NMR (400 MHz, CDCl_3_) δ 10.69 (s, 1H), 7.86 (dd, *J* = 8.0, 1.7 Hz, 1H), 7.51–7.43 (m, 1H), 7.19 (d, *J* = 8.7 Hz, 1H), 7.00 (d, *J* = 7.7 Hz, 1H), 6.92–6.85 (m, 1H), 6.77 (dd, *J* = 8.7, 2.7 Hz, 1H), 6.63 (d, *J* = 2.7 Hz, 1H), 4.74–4.63 (m, 2H), 4.33–4.26 (m, 2H), 2.31 (d, *J* = 12.7 Hz, 1H), 2.23 (dd, *J* = 12.4, 1.9 Hz, 1H), 1.97–1.68 (m, 5H), 1.60–1.41 (m,3H), 1.31 (s, 3H), 1.22 (s, 3H). ^13^C NMR (300 MHz, CDCl_3_) δ 184.8, 170.0, 161.7, 156.1, 142.6, 136.5, 135.9, 130.2, 125.5, 119.2, 117.6, 114.3, 112.8, 112.3, 65.7, 63.8, 60.4, 47.4, 44.7, 38.1, 36.8, 36.7, 30.2, 25.2, 21.7, 18.5, 16.2. HRMS: m/z calcd for C_26_H_30_O_6_ [M + H]^+^ 440.2115, found 440.2146.

*Compound* ***27***. White solid; mp: 128–129 °C; ^1^H NMR (400 MHz, CDCl_3_) δ 8.09–8.06 (m, 1H), 7.58 (t, *J* = 7.9 Hz, 1H), 7.48–7.43 (m, 1H), 7.19 (d, *J* = 8.8 Hz, 1H), 6.78 (dd, *J* = 8.7, 2.8 Hz, 1H), 6.66–6.64 (m, 1H), 4.67 (t, *J* = 9.2 Hz, 1H), 4.30 (t, *J* = 9.2 Hz, 1H), 1.90–1.83 (m, 4H), 1.83–1.73 (m, 3H),1.66–1.42 (m, 3H), 1.31 (s, 3H), 1.22 (s, 3H). ^13 ^C NMR (300 MHz, CDCl_3_) δ 183.0, 166.0, 154.2, 140.5, 134.5, 133.3, 131.4, 130.0, 126.8, 113.8, 111.4, 66.4, 63.9, 51.5, 43.9, 38.4, 37.6, 37.4, 30.1, 28.1, 24.7, 20.1, 19.7. HRMS: m/z calcd for C_26_H_29_ClO_5_ [M + H]^+^ 457.1776, found 457.1774.

*Compound* ***28***. White solid; mp: 126–127 °C; ^1^H NMR (400 MHz, CDCl_3_) δ 7.88 (dd, *J* = 8.1, 1.4 Hz, 1H), 7.33–7.25 (m, 1H), 7.18 (d, *J* = 8.7 Hz, 1H), 6.77 (dd, *J* = 8.7, 2.7 Hz, 1H), 6.71–6.60 (m, 3H), 4.62 (t, *J* = 9.6, 2H), 4.28 (t, *J* = 9.6, 2H), 3.01–2.83 (m, 2H), 2.36–2.18 (m, 2H), 1.96–1.82 (m, 4H), 1.82–1.73 (m, 2H), 1.31 (s, 3H), 1.22 (s, 3H). ^13 ^C NMR (300 MHz, CDCl_3_) δ 168.0, 156.2, 150.5, 142.4, 136.4, 134.2, 131.5, 125.4, 116.7, 116.3, 114.3, 112.8, 110.6, 66.0, 62.9, 47.4, 44.8, 38.1, 36.8, 36.7, 30.2, 25.2, 21.7, 18.5, 16.2. HRMS: m/z calcd for C_26_H_31_NO_5_ [M + H]^+^ 439.2275, found 439.2271.

*Compound* ***29***. White solid; mp: 158–159 °C; ^1^H NMR (400 MHz, CDCl_3_) δ 8.79 (d, *J* = 4.7 Hz, 1H), 8.14 (d, *J* = 7.8 Hz, 1H), 7.86 (t, *J* = 7.8 Hz, 1H), 7.56–7.47 (m, 1H), 7.16 (d, *J* = 8.8 Hz, 1H), 6.74 (s, 1H), 6.61 (d, *J* = 7.9 Hz, 1H), 4.76 (t, *J* = 9.2 Hz, 1H), 4.33 (t, *J* = 9.2 Hz, 1H), 3.01–2.83 (m, 2H), 2.36–2.18 (m, 2H), 1.95–1.83 (m, 4H), 1.83–1.70 (m, 2H), 1.31 (s, 3H), 1.22 (s, 3H). ^13 ^C NMR (300 MHz, CDCl_3_) δ 168.7, 155.9, 142.7, 136.5, 125.5, 114.2, 112.6, 65.4, 64.6, 56.4, 53.5, 47.3, 44.8, 43.8, 38.2, 36.8, 36.7, 30.2, 25.7, 25.2, 21.7, 18.6, 16.4. HRMS: m/z calcd for C_25_H_29_NO_5_ [M + H]^+^ 424.2118, found 424.2115.

*Compound* ***30***. White solid; mp: 161–162 °C; ^1^H NMR (400 MHz, CDCl_3_) δ 8.04 (d, *J* = 8.7 Hz, 1H), 7.52 (t, *J* = 7.5 Hz, 1H), 7.38 (t, *J* = 7.3 Hz, 1H), 7.30 (d, *J* = 8.4 Hz, 1H), 7.16 (d, *J* = 8.8 Hz, 1H), 6.75 (dd, *J* = 8.7, 2.6 Hz, 1H), 6.61 (d, *J* = 2.5 Hz, 1H), 4.68–4.61 (m, 2H), 4.33–4.22 (m, 2H), 4.06 (s, 2H), 2.94–2.70 (m, 2H), 2.29 (d, *J* = 12.5 Hz, 1H), 2.20 (d, *J* = 13.9 Hz, 1H), 1.94–1.84 (m, 2H), 1.84–1.68 (m, 3H), 1.60–1.41 (m, 2H), 1.28 (s, 3H), 1.20 (s, 3H). ^13 ^C NMR (300 MHz, CDCl_3_) δ 185.1, 177.0, 167.3, 156.1, 142.5, 136.4, 135.4, 132.8, 132.4 131.3, 129.4 127.7, 125.4, 114.2, 112.8, 65.7, 63.8, 47.4, 44.7, 40.7, 38.1, 36.7, 36.6, 30.1, 25.2, 21.6, 18.5, 16.2. HRMS: m/z calcd for C_28_H_32_O_7_ [M + Na]^+^ 504.2221, found 504.2071

*Compound* ***31***. White solid; mp: 116–117 °C; ^1^H NMR (400 MHz, CDCl_3_) δ 7.15 (d, *J* = 8.8 Hz, 1H), 6.71 (dd, *J* = 8.7, 2.6 Hz, 1H), 6.57 (s, 1H), 4.48–4.31 (m, 2H), 4.20–3.89 (m, 2H), 3.03–2.73 (m, 4H), 2.25 (dd, *J* = 32.2, 11.8 Hz, 2H), 2.04 (s, 1H), 1.88–1.82 (m, 2H), 1.82–1.76 (m, 2H), 1.76–1.71 (m, 2H), 1.55–1.36 (m, 5H), 1.28 (s, 3H), 1.20 (s, 3H). ^13 ^C NMR (300 MHz, CDCl_3_) δ 185.1, 179.9, 173.6, 156.1, 142.4, 136.3, 125.3, 114.3, 114.2, 112.8, 112.7, 65.8, 63.0, 60.4, 47.3, 44.7, 42.5, 42.4, 38.1, 36.7, 36.6, 30.1, 25.2, 21.6, 21.1, 18.5, 16.2, 14.2. HRMS: m/z calcd for C_27_H_36_O_7_ [M + H]^+^ 474.2534, found 474.2570.

*Compound* ***32***. White solid; mp: 109–110 °C; ^1^H NMR (400 MHz, CDCl_3_) δ 7.15 (d, *J* = 8.7 Hz, 1H), 6.69 (d, *J* = 6.9 Hz, 1H), 6.55 (s, 1H), 4.54 (s, 2H), 4.15 (s, 2H), 3.51 (s, 1H), 2.84 (s, 2H), 2.37–1.89 (m, 5H), 1.75 (m, 6H), 1.59–1.36 (m, 3H), 1.28 (s, 3H), 1.18 (s, 3H)^. 13 ^C NMR (300 MHz, CDCl_3_) δ 168.7, 155.9, 142.7, 136.5, 125.5, 114.2, 112.6, 65.4, 64.6, 56.4, 53.5, 47.3, 44.8, 43.8, 38.2, 36.8, 36.7, 30.2, 25.7, 25.2, 21.7, 18.6, 16.4. HRMS: m/z calcd for C_24_H_33_NO_5_ [M + H]^+^ 417.2431, found 417.2462.

*Compound* ***33***. White solid; mp: 48–49 °C; ^1^H NMR (400 MHz, CDCl_3_) δ 7.16 (d, *J* = 10.0 Hz, 1H), 6.72 (d, *J* = 8.6 Hz, 1H), 6.58 (s, 1H), 4.39–3.66 (m, 6H), 3.34–3.29 (m, 2H), 2.99–2.78 (m, 2H), 2.38–2.05 (m, 4H), 1.80–1.63 (m, 4H), 1.54–1.38 (m, 2H), 1.26 (s, 3H), 1.20 (s, 2H). ^13^C NMR (300 MHz, CDCl_3_) δ 183.4, 156.2, 142.6, 136.5, 125.4, 114.2, 112.5, 69.1, 61.4, 53.1, 47.3, 45.8, 44.9, 38.2, 36.8, 36.7, 30.2, 25.2, 21.6, 18.6, 16.4. HRMS: m/z calcd for C_25_H_35_NO_5_ [M + H]^+^ 431.2588, found 431.2615.

*Compound* ***34***. White solid; mp: 189–190 °C; ^1^H NMR (400 MHz, *d_6_*-DMSO) δ 12.72 (s, 2H), 8.67 (d, *J* = 4.9 Hz, 2H), 8.23 (dd, *J* = 8.4, 4.8 Hz, 2H), 8.04 (dd, *J* = 14.0, 8.7 Hz, 2H), 7.14 (d, *J* = 8.7 Hz, 1H), 6.76 (d, *J* = 10.5 Hz, 1H), 6.64 (s, 1H), 4.66 (s, 2H), 4.34 (s, 2H), 2.25 (d, *J* = 12.0 Hz, 1H), 2.01 (d, *J* = 11.6 Hz, 1H), 1.78–1.48 (m, 2H), 1.15 (s, 3H), 1.09 (s, 3H). ^13 ^C NMR (300 MHz, *d_6_*-DMSO) δ 179.9, 167.6, 166.0, 156.3, 142.6, 136.3, 134.8, 134.5, 130.9, 130.7, 130.6, 130.4, 130.3, 129.3, 126.5, 126.0, 125.8, 114.5, 113.2, 66.1, 64.4, 46.8, 45.3, 38.3, 36.7, 30.2, 25.3, 21.5, 18.6, 16.8. HRMS: m/z calcd for C_31_H_32_O_7_ [M + H]^+^ 517.2221, found 517.2231.

### 4.2. Biological assays

#### 4.2.1. PTP1B inhibition assay

The inhibitory assay was performed using human PTP1B (purchased from Sigma) and p-nitrophenylphosphate (pNPP) as substrate by UV absorption. 400 nmol pNPP were dissolved in H_2_O and 48 μL of the solution was added to a 100 μL reaction. The indicated amount of synthetic compounds was diluted in DMSO at a concentration of 5 mM. The PTP1B was diluted in pH 7.2 buffer contained 50 mM HEPES, 3 mM dithiothreitol, 2 mM EDTA and 100 mM NaCl. About 50 μL PTP1B dilution was added, and the mixture incubated at 37 °C for 30 min. 1N NaOH was added to terminate the reaction. UV–Vis absorption was detected at 410 nm using a Tecan Infinite M1000 plate reader.

#### 4.2.2. Glucose uptake assay

HepG2 cells were grown in DMEM with 10% foetal bovine serum, 100 mg/mL of streptomycin, and 100 U/mL of penicillin, in a humidified atmosphere of 5% CO_2_ at 37 °C. HepG2 cells were cultured in 96-well tuft plates. After overnight incubation, the cells were serum starved for 12 h. Then, the cells were treated with insulin (6 × 10^−6^–5 × 10^−5 ^mmol/L) for 0–60 h to induce insulin resistance. HepG2 cells were then cultured for another 24 h in the compound (40 μM) or rosiglitazone (10 μM, available from Bied Pharmaceutical Technology Co., Ltd., Shanghai, China) containing DMEM. After cultivation, the glucose content was determined by the glucose oxidase method (the glucose analysis kit was purchased from Rongsheng Biopharmaceutical Co., Ltd., Shanghai, China). The sample and the kit working solution were thoroughly mixed at a ratio of 1:100, and placed in a 37 °C water bath for 15 min. The absorbance was measured at 506 nm using a microplate reader (SpectraMax i3x, Molecular Devices). The residual glucose concentration is expressed as *A*_X_/*A*_0_ × 5.55 mmol/L (*A*_X_: OD value of sample; *A*_0_: OD value of glucose standard solution).

#### 4.2.3. Animal experiment

During the experiment, the male Kunming mice with 25–30 g body weight were fasted but received water prior to induction of diabetes. 110 mg/kg of nicotinamide and 65 mg/kg of streptozotocin (Macklin in Biochemical Technology Co., Ltd., Shanghai, China) were dissolved in pH 4.5 citrate buffer and intraperitoneally administrated to induce NIDDM mice model. The mice were intraperitoneally administered with a compound or rosiglitazone (100 mg/kg) dissolved in saline containing 1% DMSO and 10% Tween 80. Hyperglycaemia was confirmed by a plasma glucose increase of more than 4.5 mmol/L measured by a blood glucose metre (Sinocare GA-3, Changsha, China). Glucose concentrations from the tail of the mice were measured at 0, 0.5, 1, 2, 3 and 4 h by the blood glucose metre. Calculate the percentage of blood glucose by using the following formula: variation of glycaemia% = (*A*_X_ − *A*_0_)/*A*_0_ × 100% (*A*_X_: blood glucose at selected time; *A*_0_: initial blood glucose).

## Supplementary Material

Supplemental Material
