# Supplementary material for: Design and synthesis of tricyclic terpenoid derivatives as novel PTP1B inhibitors with improved pharmacological property and in vivo antihyperglycaemic efficacy
Source: J Enzyme Inhib Med Chem. 2019 Nov 19;35(1):152–64. doi: 10.1080/14756366.2019.1690481 (PMC6882489; doi:10.1080/14756366.2019.1690481)
Supplement: Supplemental Material [file IENZ_A_1690481_SM8822.pdf]

Supporting information

Design and synthesis of tricyclic terpenoid derivatives as novel PTP1B inhibitors with improved pharmacological property and in vivo antihyperglycemic efficacy

Lingling Yang[a], Feng Chen[a], Cheng Gao[a], Jiabao Chen[a], Junyan Li[a], Siyan Liu[a], Yuanyuan Zhang[b], Zhouyu Wang[b, **] and Shan Qian[a, *]

[a] *Department of Pharmaceutical Engineering, College of Food and Bioengineering, Xihua University, Chengdu 610039, China.*

[b] *Department of Chemistry, College of Science, Xihua University, Chengdu 610039, China*

CONTACT Shan Qian (qians33@163.com); Zhouyu Wang (zhouyuwang77@gmail.com); College of *Food and Bioengineering*, *Xihua University, Chengdu 610039, China.*

Table of Contents

1. Cell viability in the MTT assay

2. The NMR spectra of compounds

Cell viability in the MTT assay

The toxicities of the compounds were evaluated by (3-(4, 5-dimethylthiazolyl-2)-2, 5-diphenyltetrazolium bromide) test. HepG2 cells were routinely grown in Dulbecco's Modified Eagle Medium (DMEM) supplemented with 10% (v/v) fetal bovine serum, streptomycin (100mg/mL), and penicillin (100U/mL), in a humidified atmosphere of 95% air, 5% CO_2 at 37°C. HepG2 cells were planked in a 96 well plate with a concentration of 1×10^4 cells/well and cultured in 37°C and 5%CO_2 for 24 h. Then HepG2 cells were treated with 40 μM of the synthetic compound. HepG2 cells untreated were used as control. HepG2 cells were cultured in 37℃ and 5%CO_2 for 24 h, the old medium was carefully removed and were cultured for another 4 h in MTT (0.5%) containing DMEM, then the medium was carefully removed. 150 µL/well dimethyl sulfoxide was added and oscillated gently to make crystal dissolved. The absorbance at 560 nm was measured using a microplate reader. The cell viability was expressed as a percentage of OD560 (sample)/ OD560 (control) as shown in Fig.S1.

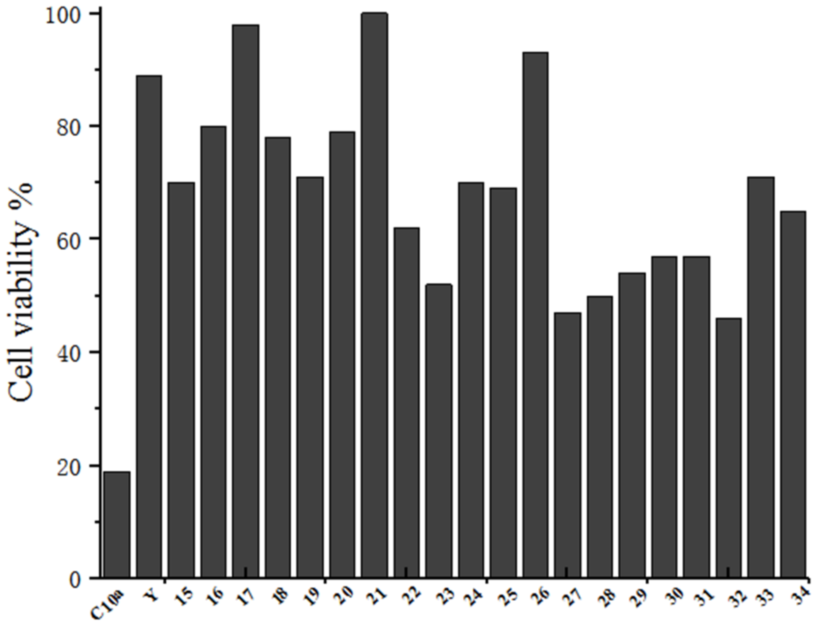

Fig. S1. Cell viability in the MTT assay.

The ^{1}H-NMR and ^{13}C-NMR spectra of the compounds

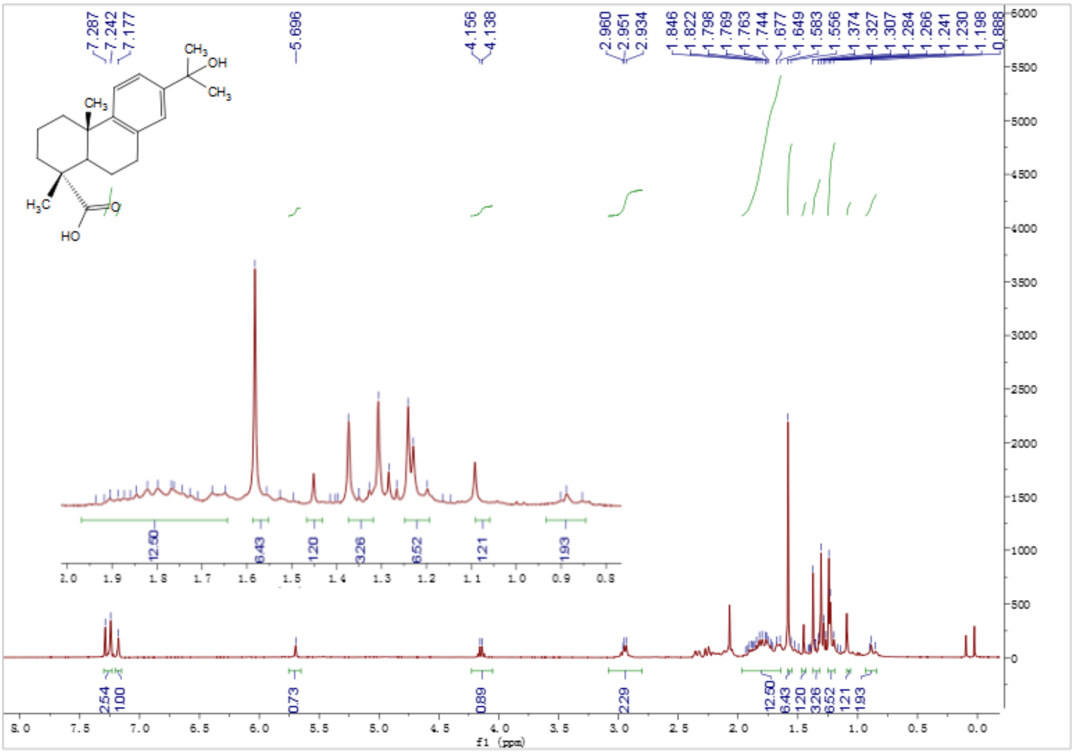

^{1}H NMR (400 MHz, $CDCl_3$) spectrum of compound 3

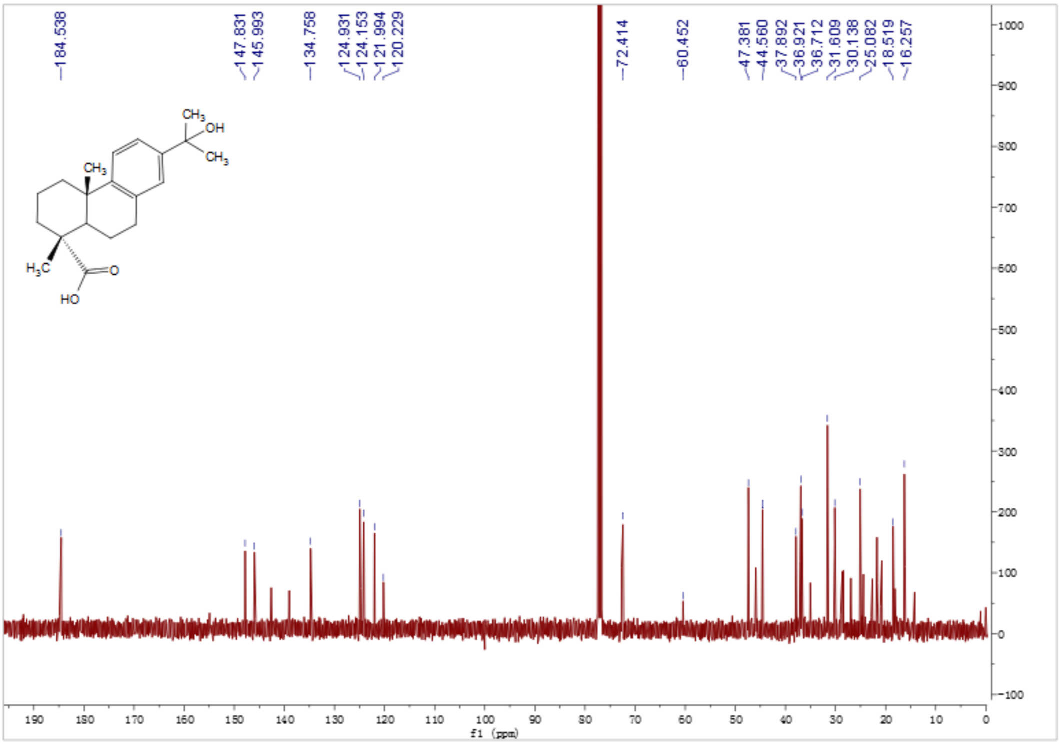

^{13}C NMR (300 MHz, $CDCl_3$) spectrum of compound 3

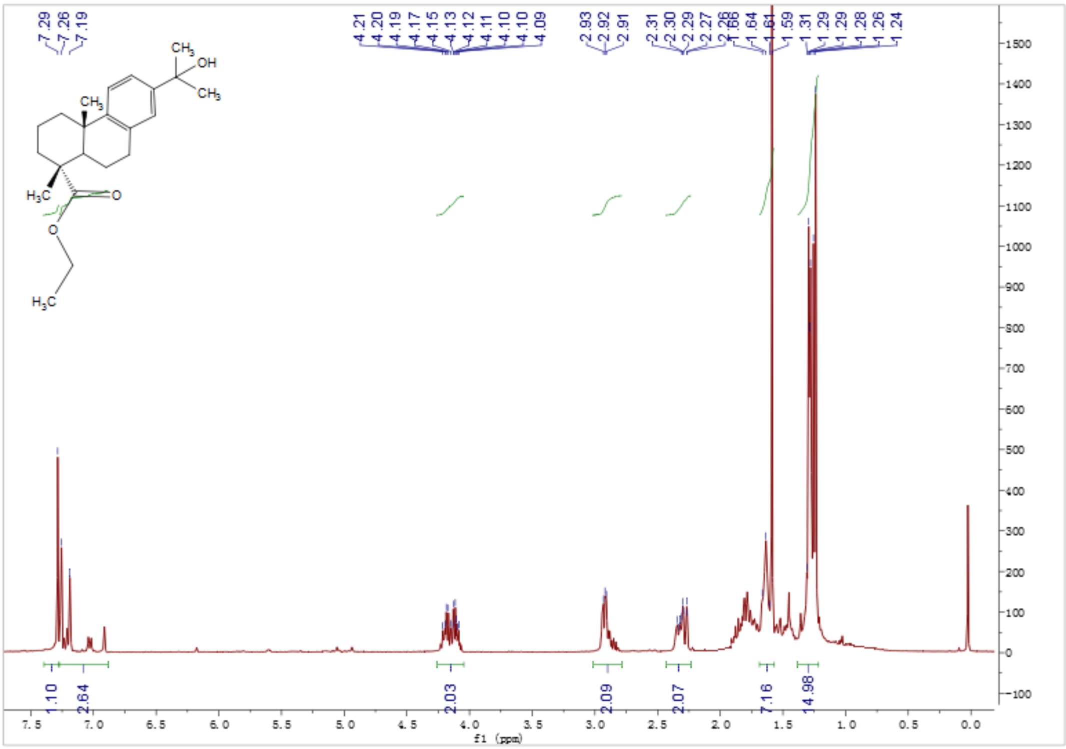

^{1}H NMR (400 MHz, $CDCl_3$) spectrum of compound 4a

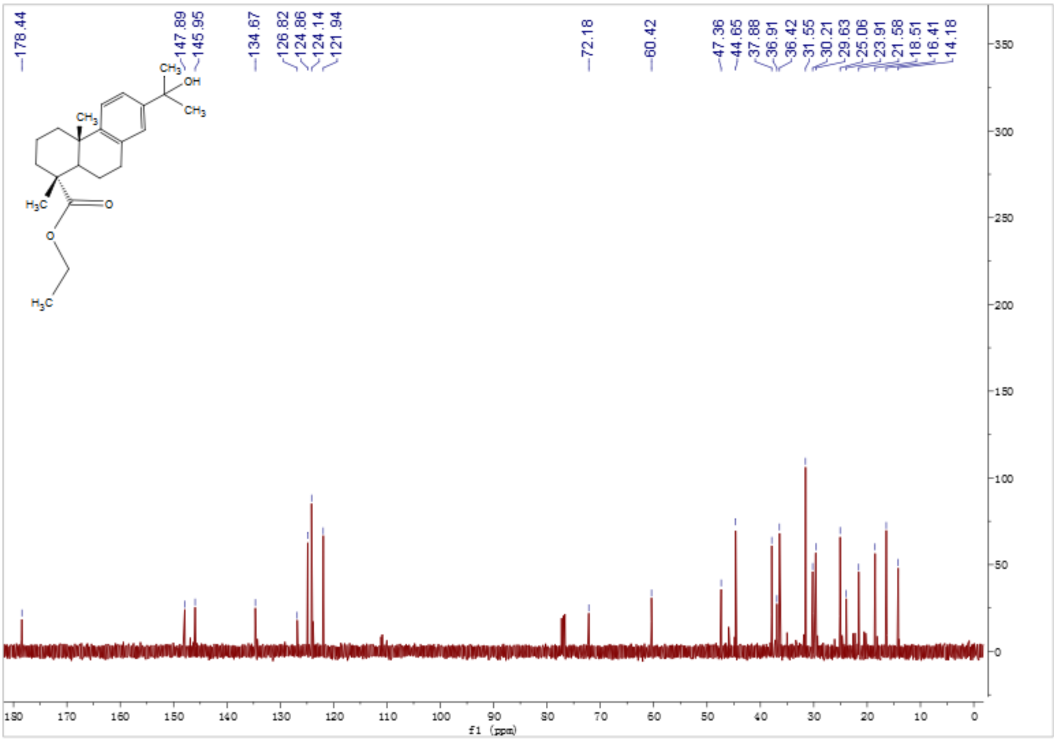

^{13}C NMR (300 MHz, $CDCl_3$) spectrum of compound 4a

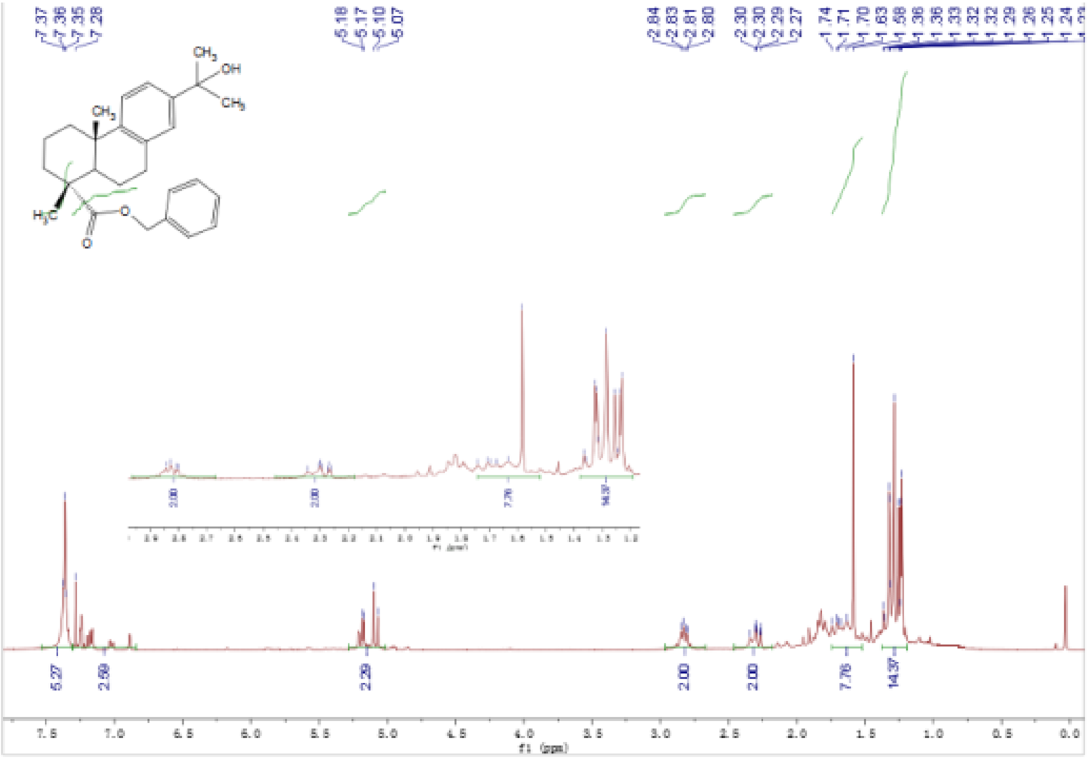

^{1}H NMR (400 MHz, $CDCl_3$) spectrum of compound 4b

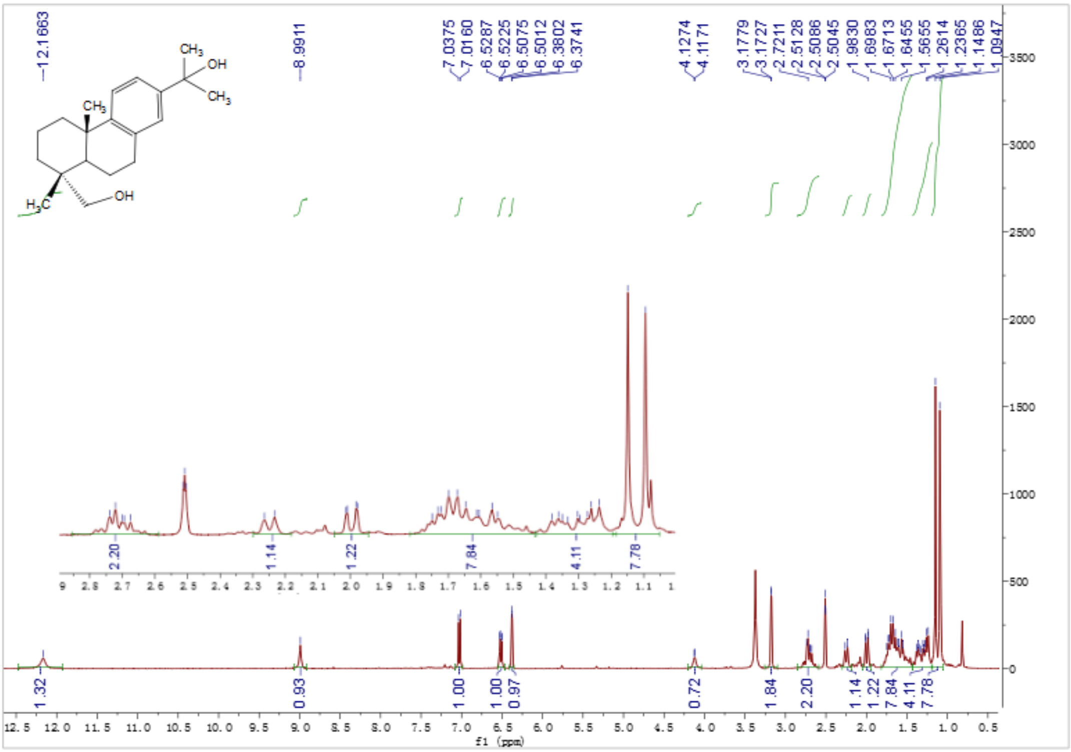

^{1}H NMR (400 MHz, DMSO) spectrum of compound 5

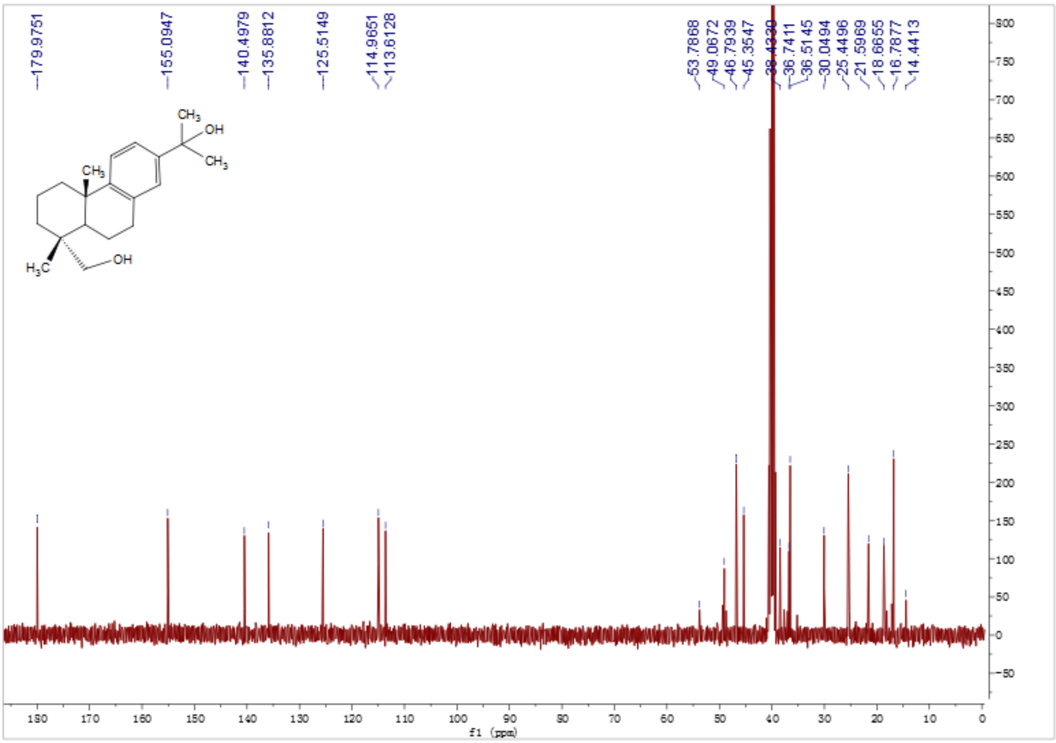

^{13}C NMR (300 MHz, $CDCl_3$) spectrum of compound 5

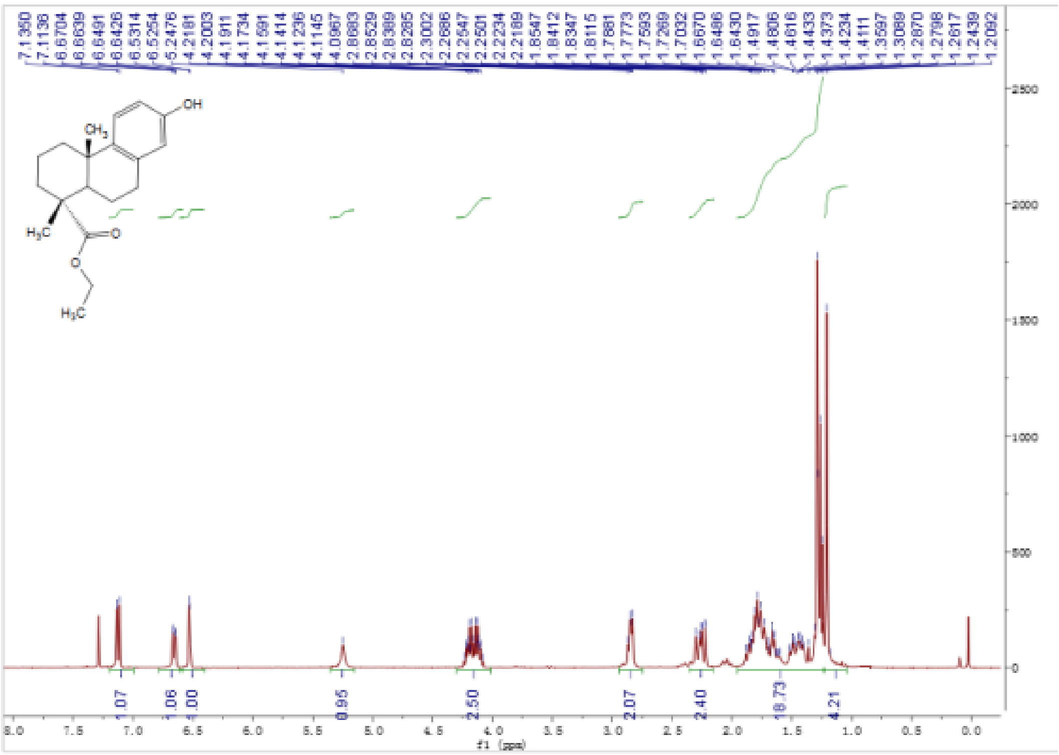

^{1}H NMR (400 MHz, $CDCl_3$) spectrum of compound 6a

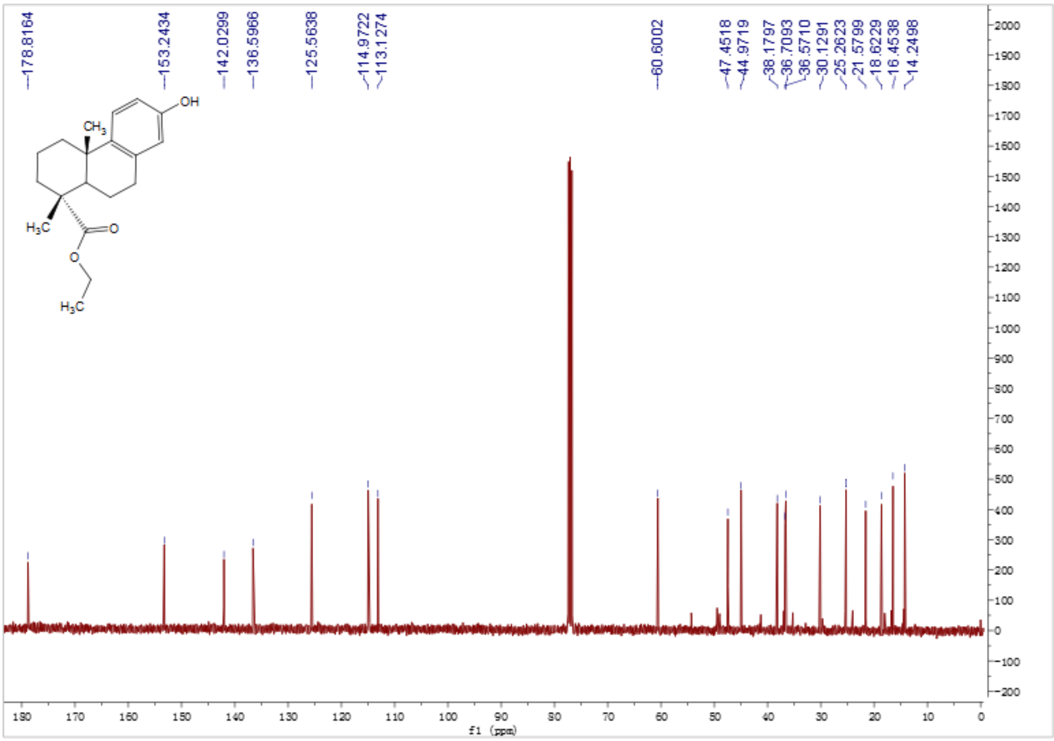

^{13}C NMR (300 MHz, $CDCl_3$) spectrum of compound 6a

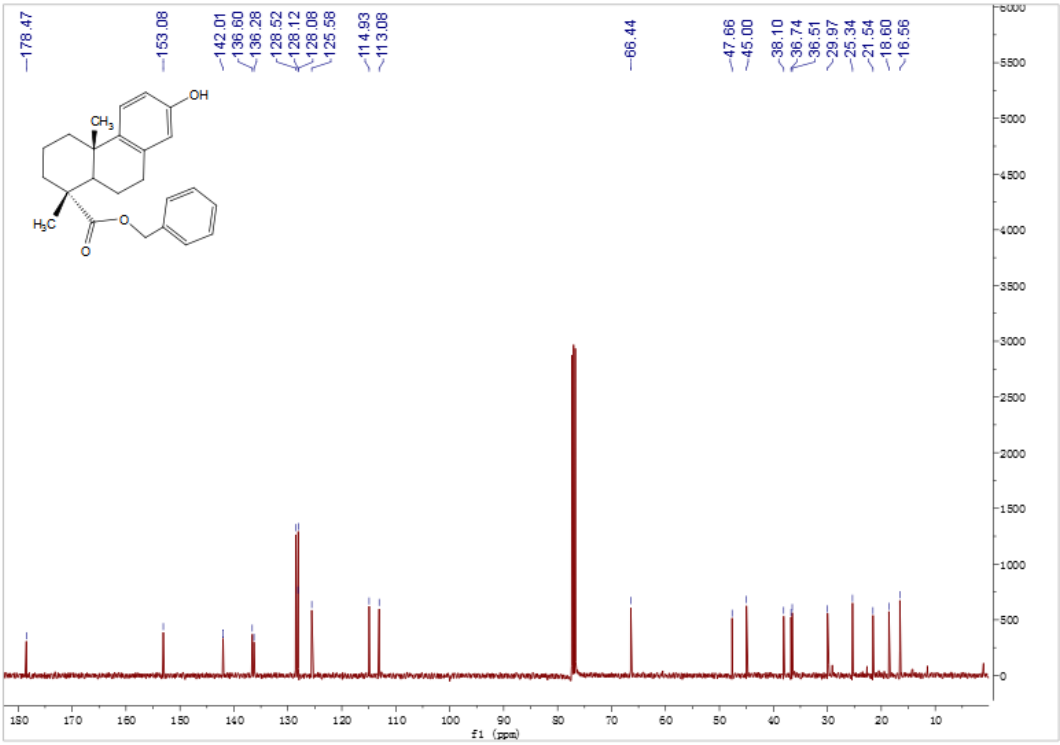

^{13}C NMR (300 MHz, $CDCl_3$) spectrum of compound 6b

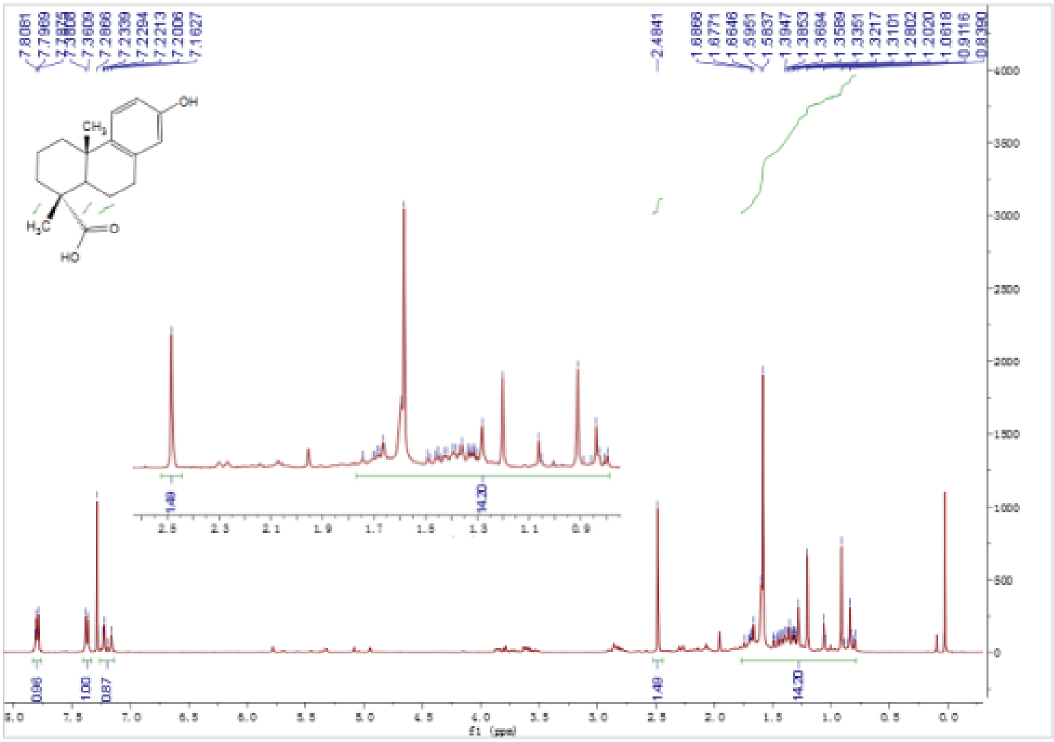

^{1}H NMR (400 MHz, $CDCl_3$) spectrum of compound 7

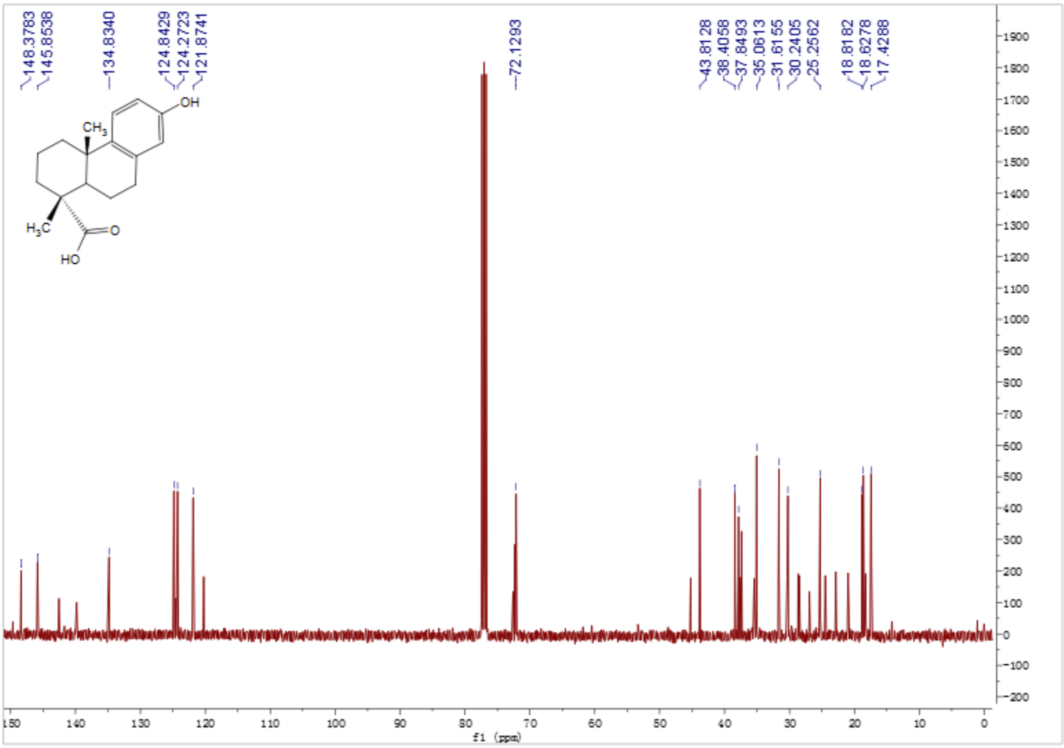

^{13}C NMR (300 MHz, $CDCl_3$) spectrum of compound 7

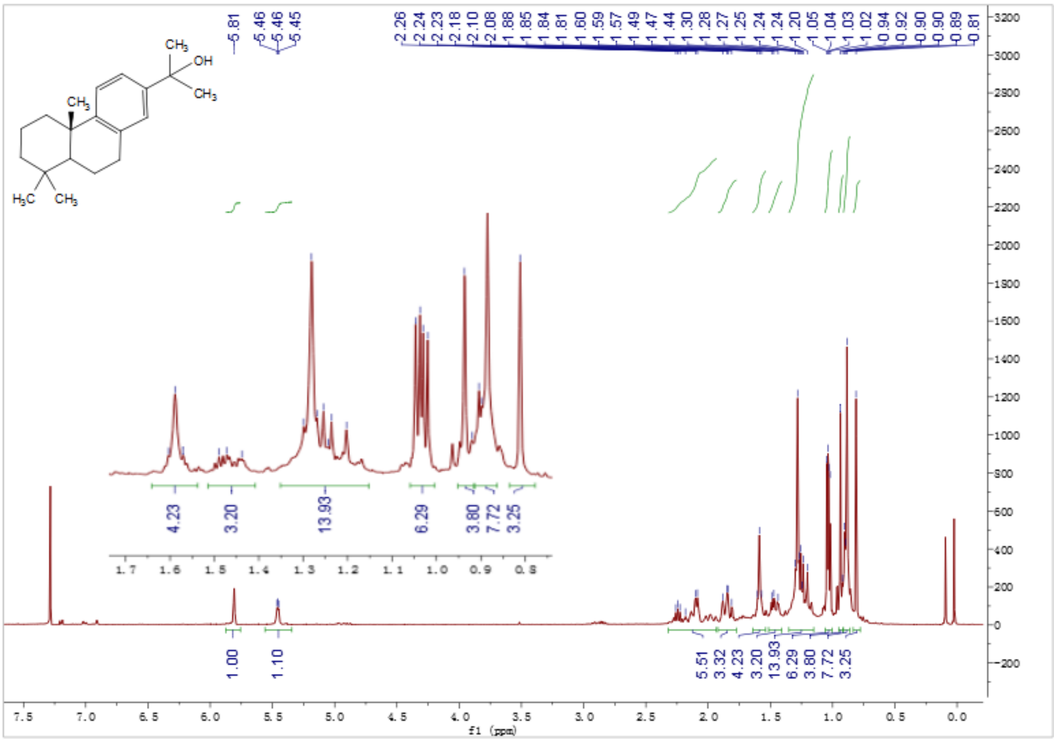

^{1}H NMR (400 MHz, $CDCl_3$) spectrum of compound 11

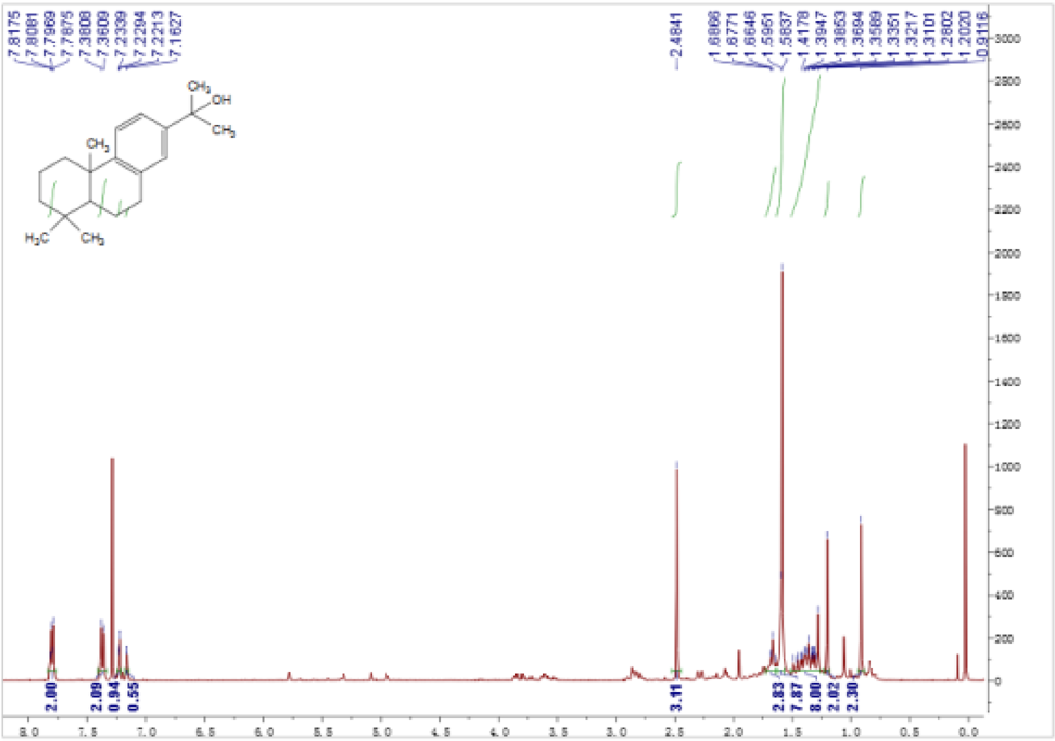

^{1}H NMR (400 MHz, $CDCl_3$) spectrum of compound 13

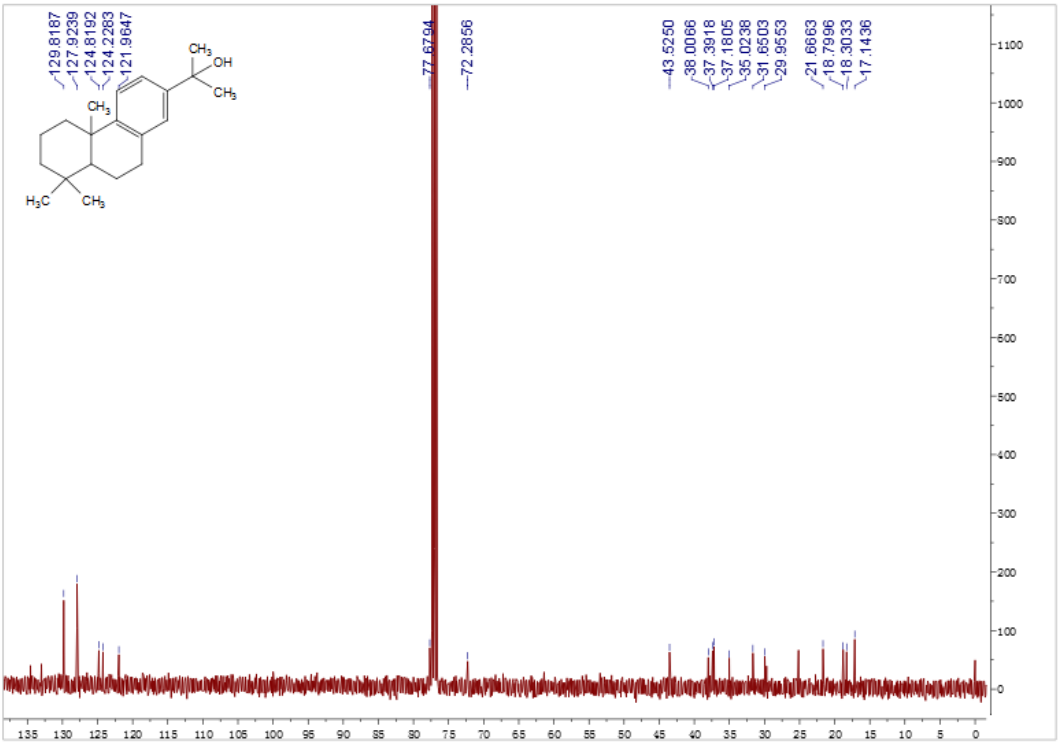

^{13}C NMR (300 MHz, $CDCl_3$) spectrum of compound 13

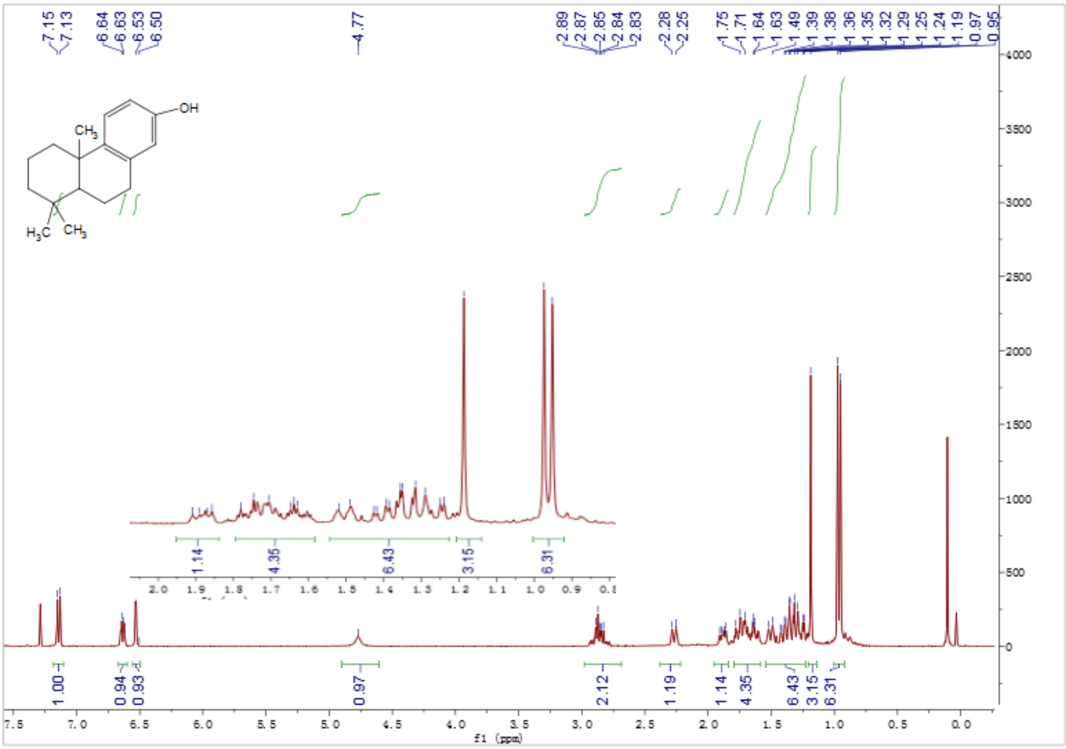

^{1}H NMR (400 MHz, $CDCl_3$) spectrum of compound 14

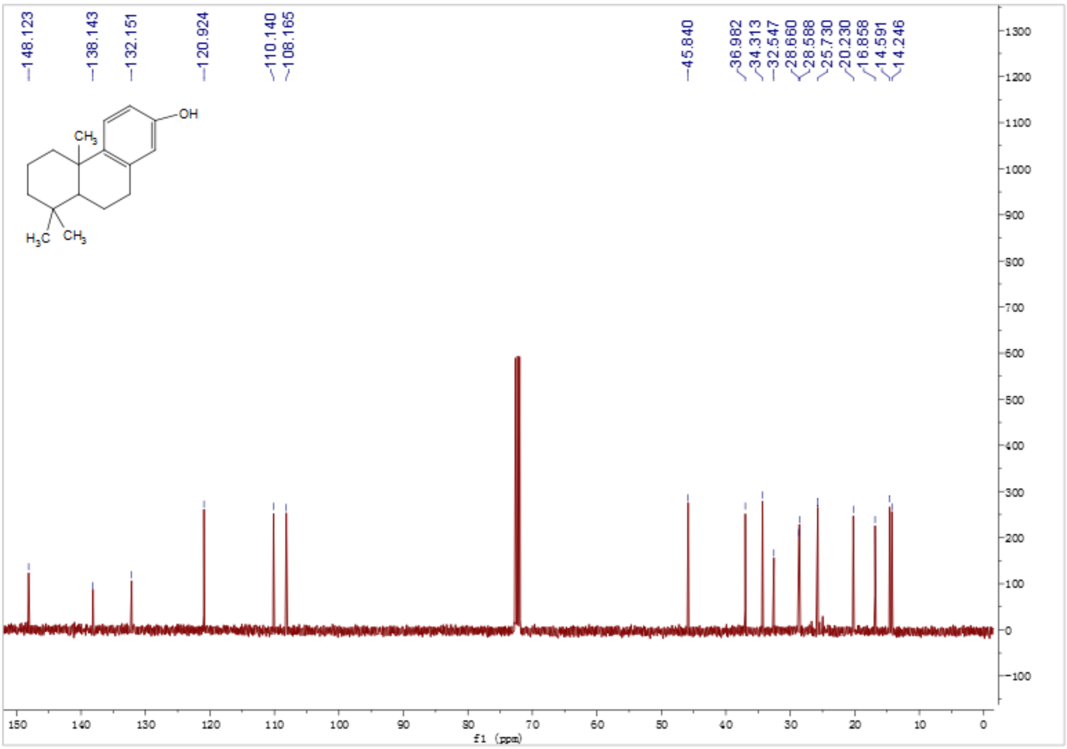

^{13}C NMR (300 MHz, $CDCl_3$) spectrum of compound 14

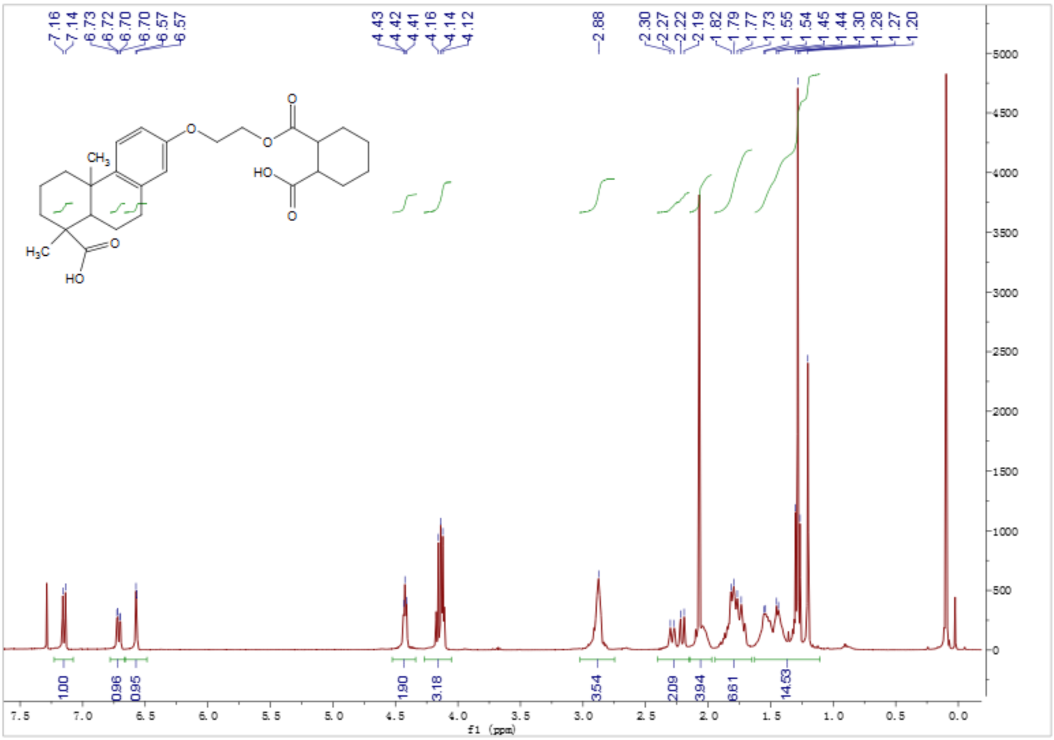

^{1}H NMR (400 MHz, $CDCl_3$) spectrum of compound 15

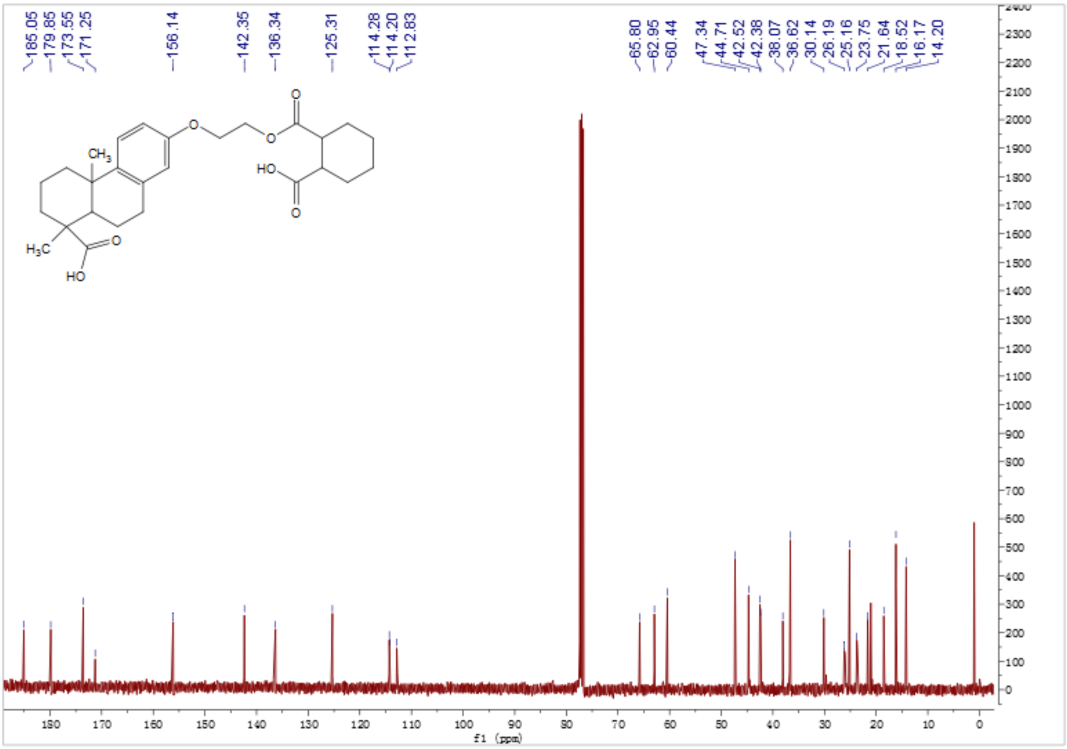

^{13}C NMR ((300 MHz, $CDCl_3$) spectrum of compound 15

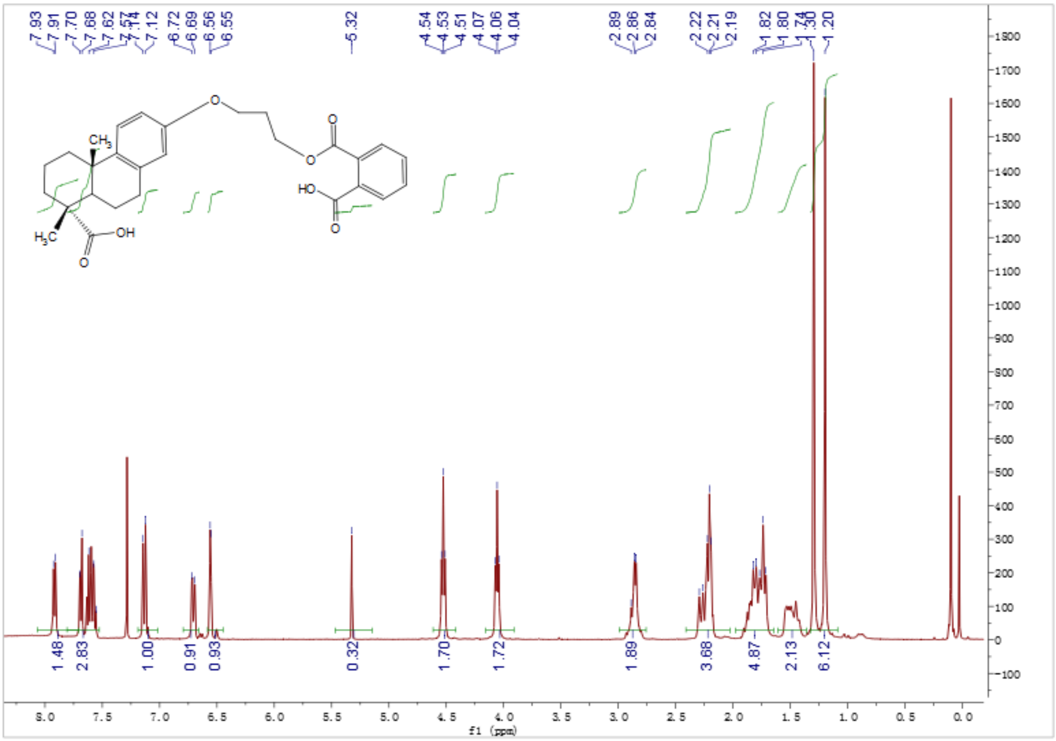

^{1}H NMR (400 MHz, $CDCl_3$) spectrum of compound 16

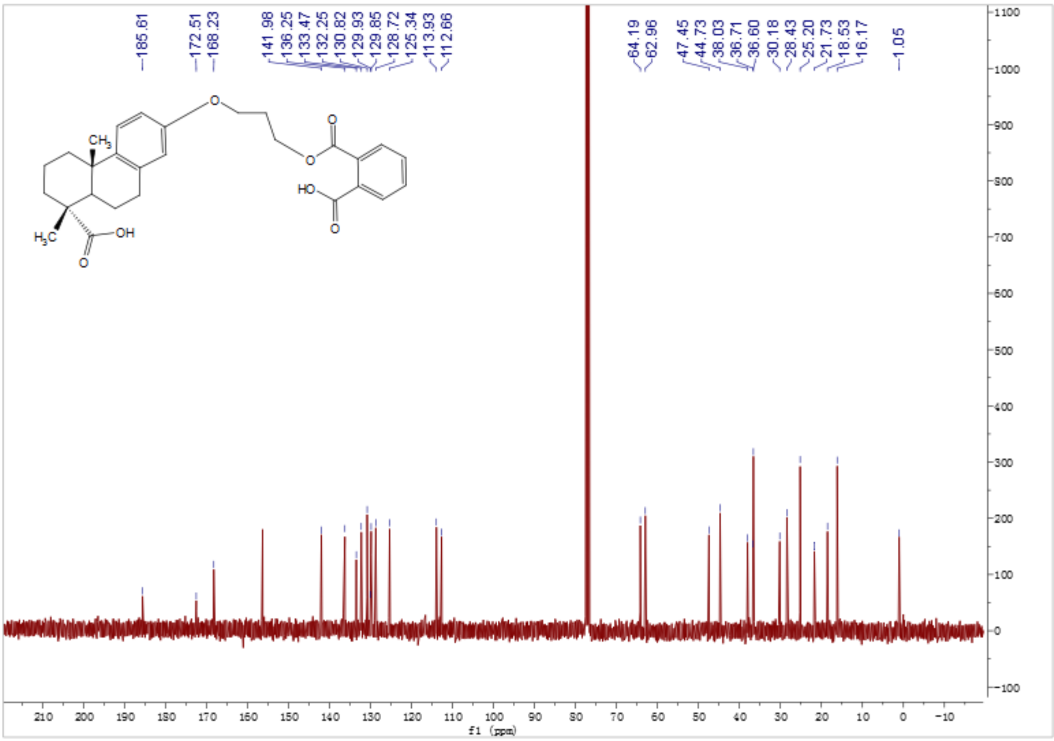

^{13}C NMR ((300 MHz, $CDCl_3$) spectrum of compound 16

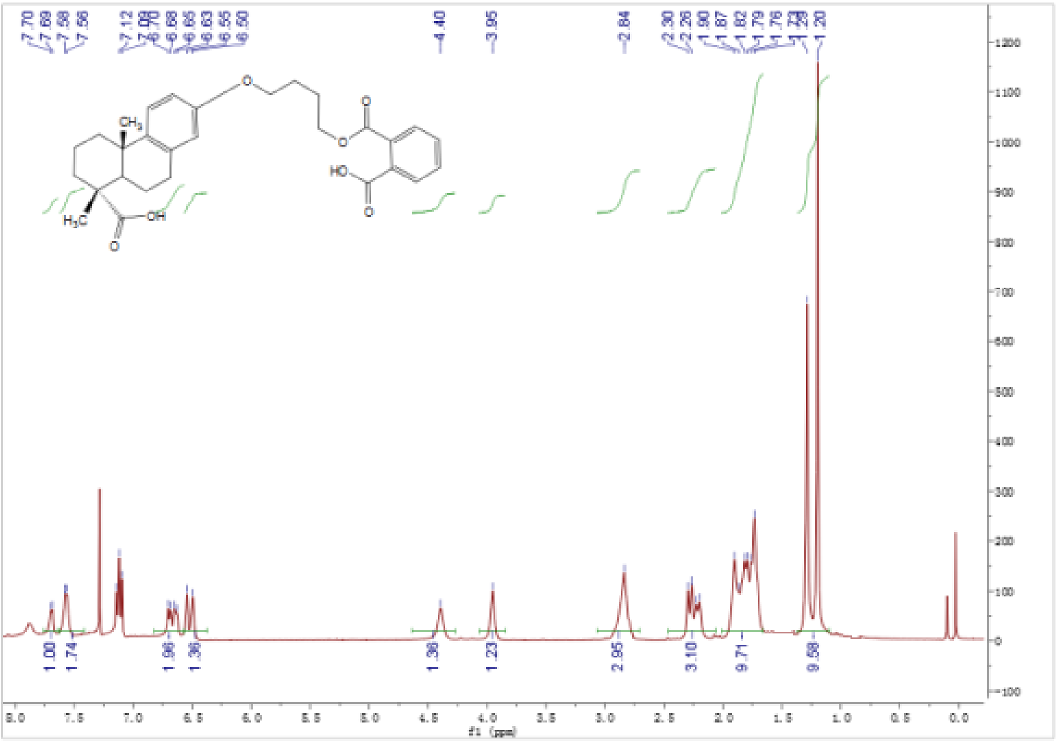

^{1}H NMR (400 MHz, $CDCl_3$) spectrum of compound 17

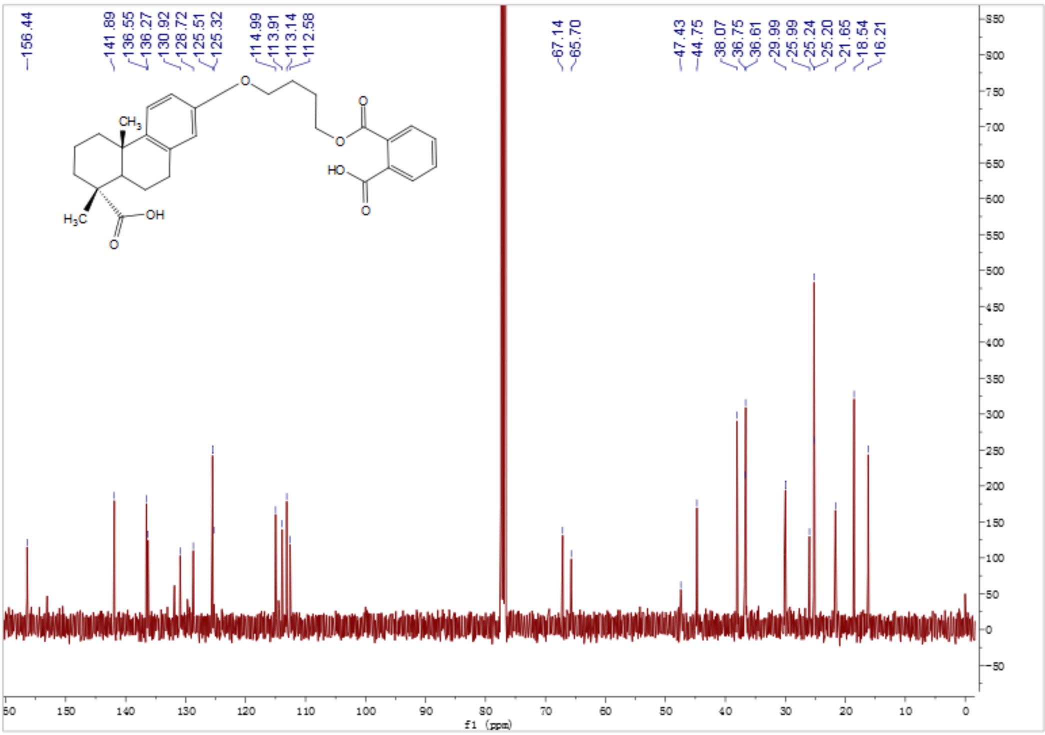

^{13}C NMR ((300 MHz, $CDCl_3$) spectrum of compound 17

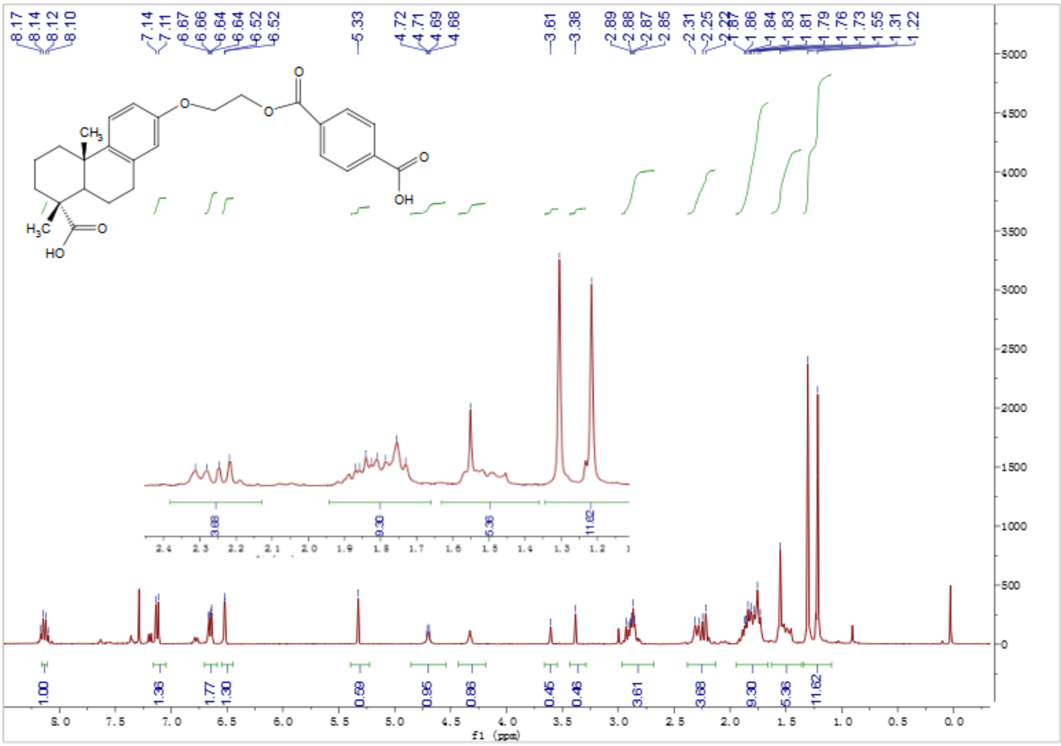

^{1}H NMR (400 MHz, CDCl3) spectrum of compound 18

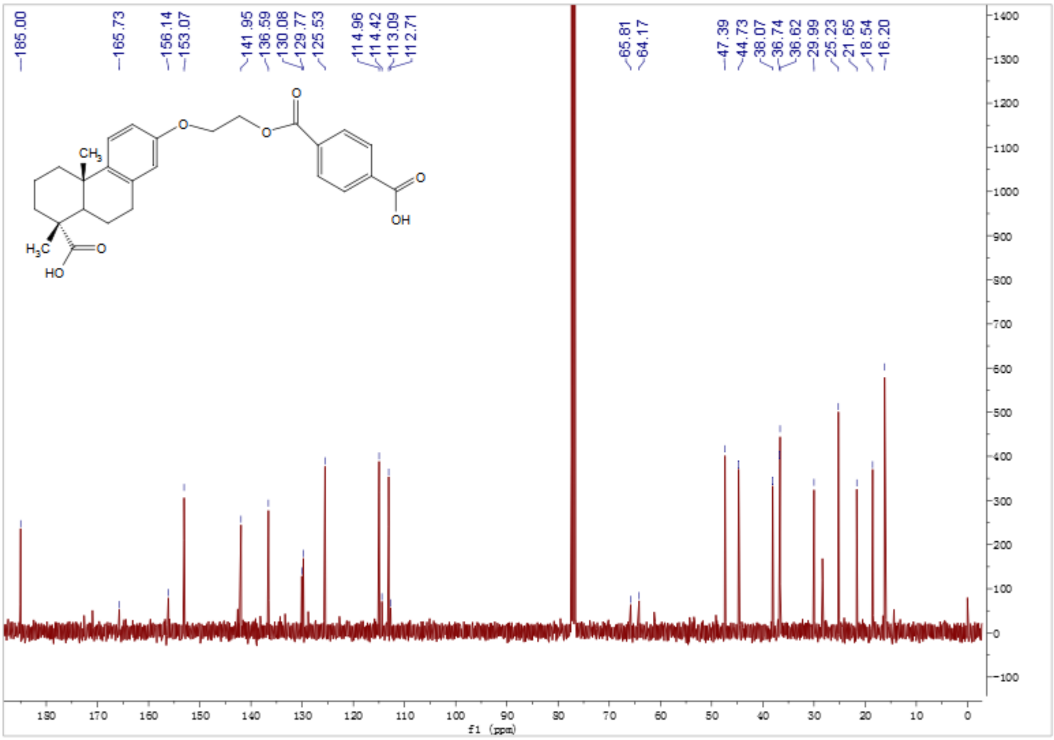

^{13}C NMR (300 MHz, $CDCl_3$) spectrum of compound 18

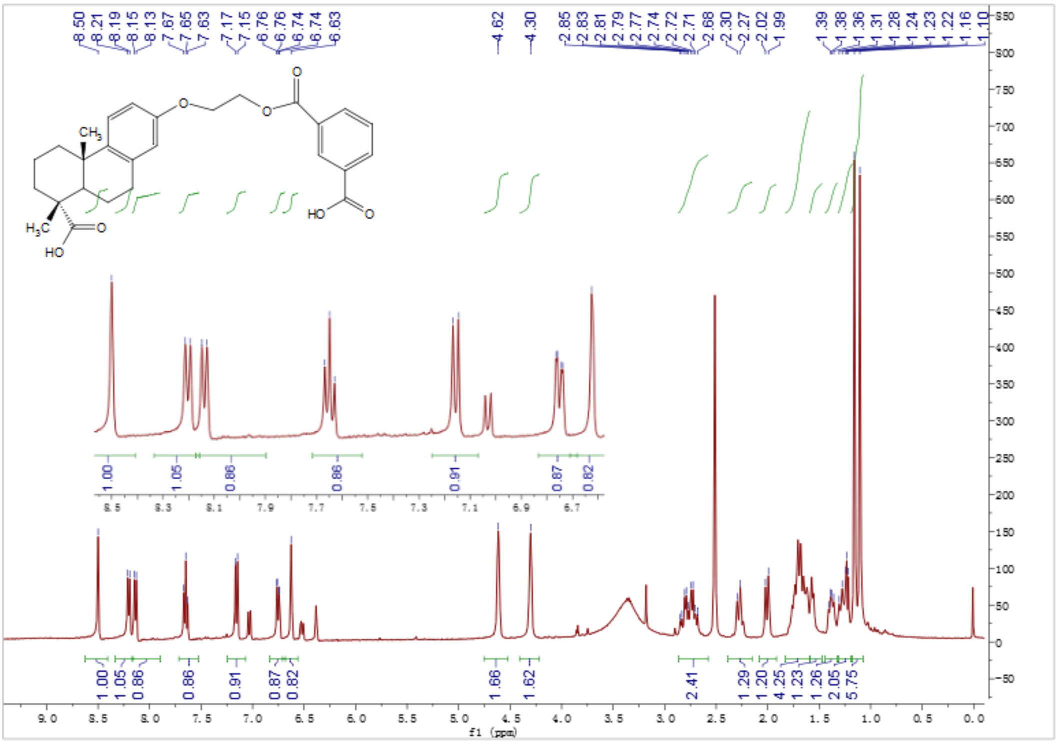

^{1}H NMR (400 MHz, d_6-DMSO) spectrum of compound 19

^{13}C NMR (300 MHz, $CDCl_3$) spectrum of compound 19

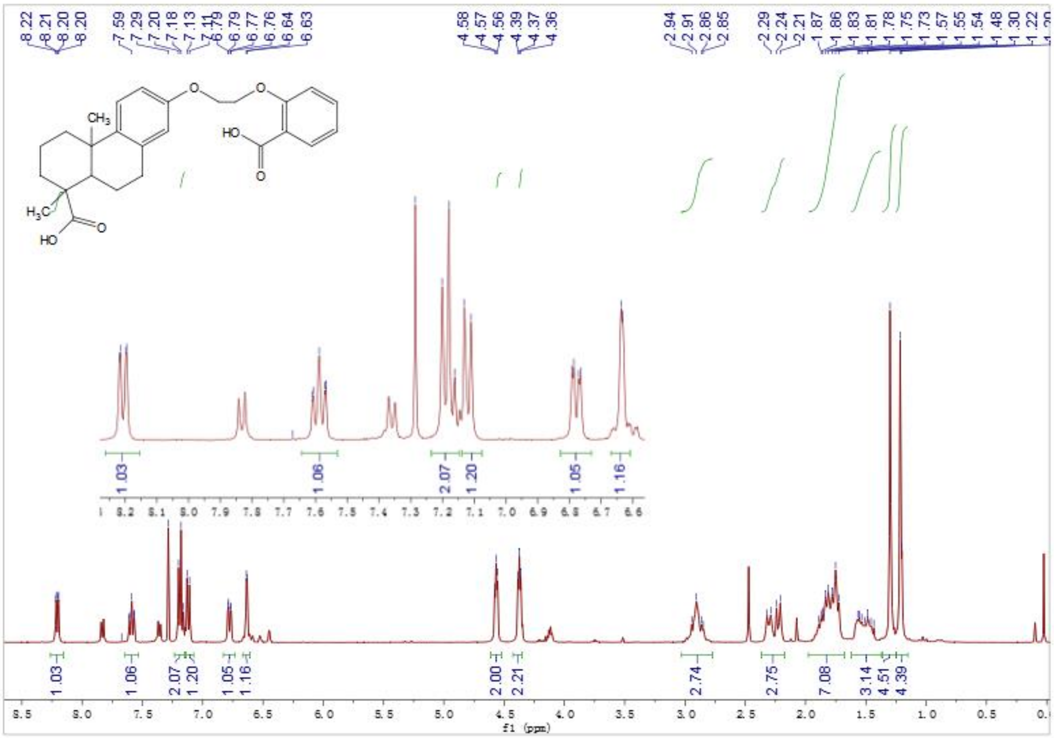

^{1}H NMR (400 MHz, $CDCl_3$) spectrum of compound 20

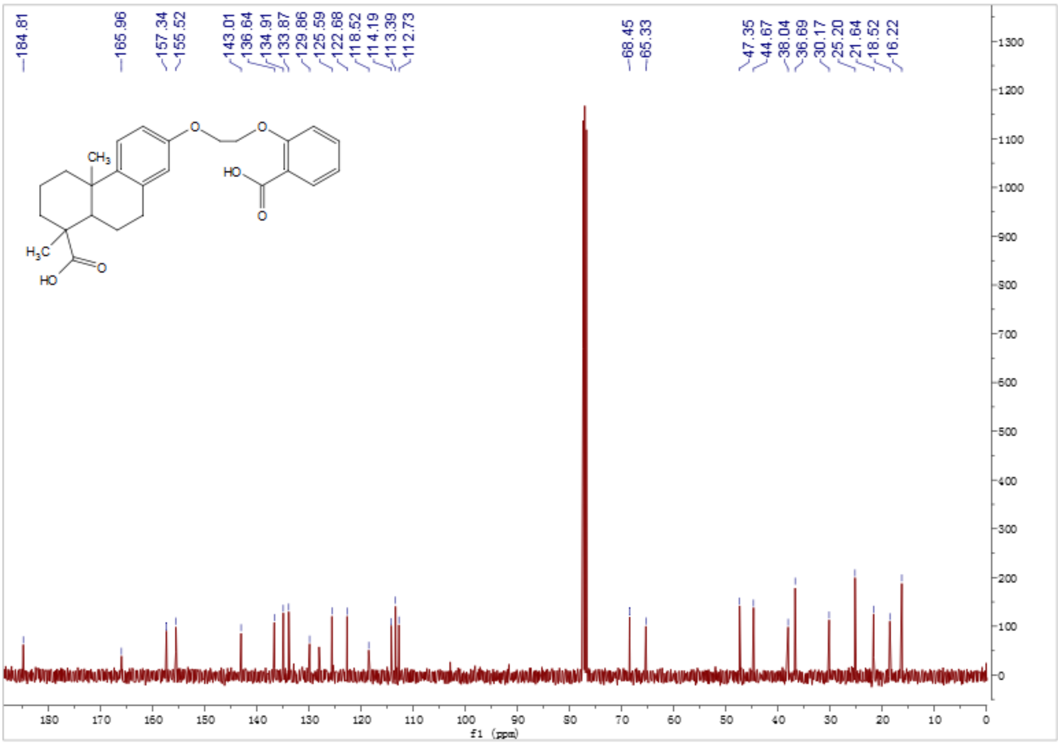

^{13}C NMR (300 MHz, $CDCl_3$) spectrum of compound 20

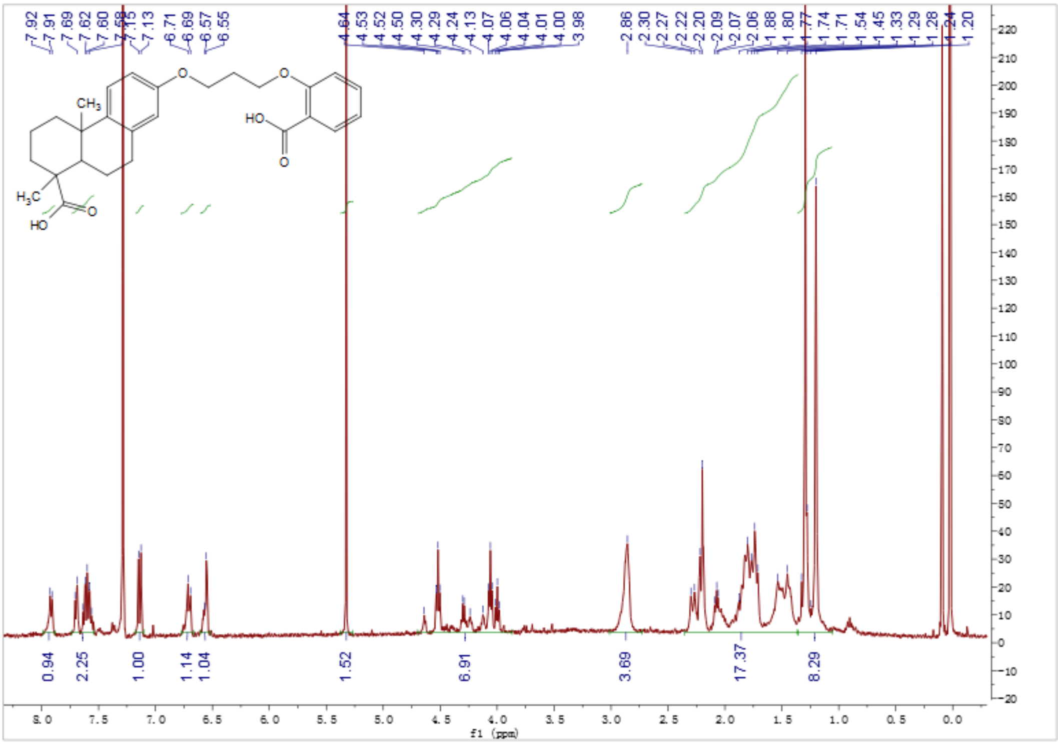

^{1}H NMR (400 MHz, CDCl3) spectrum of compound 21

^{13}C NMR (300 MHz, $CDCl_3$) spectrum of compound 21

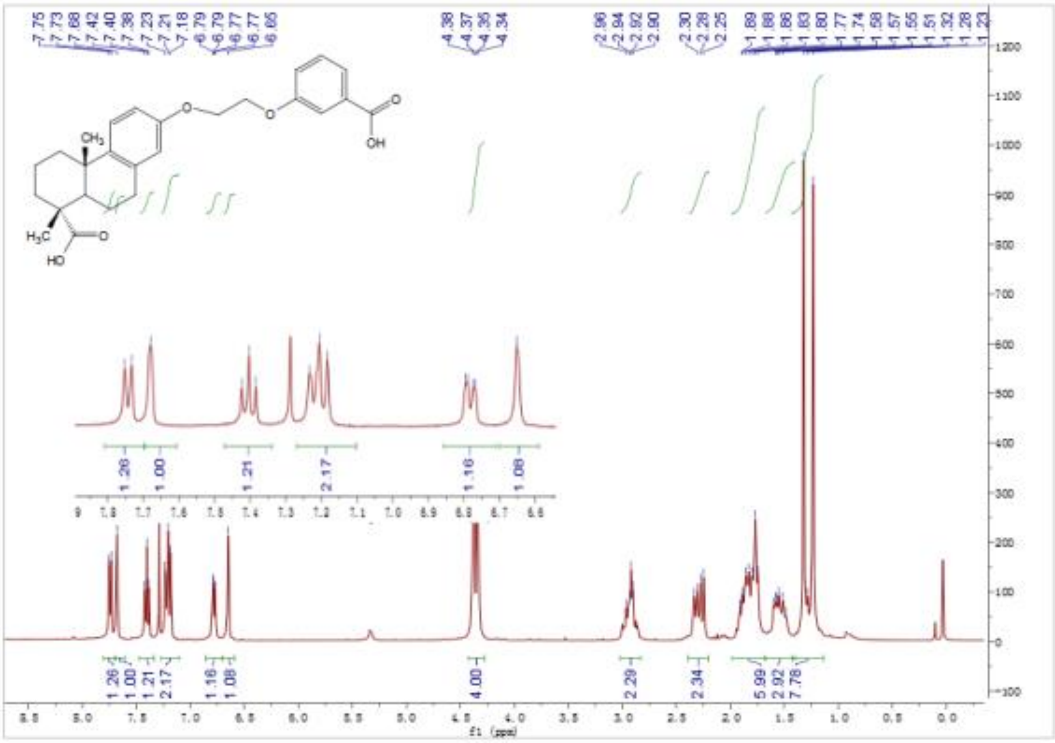

^{1}H NMR (400 MHz, $CDCl_3$) spectrum of compound 22

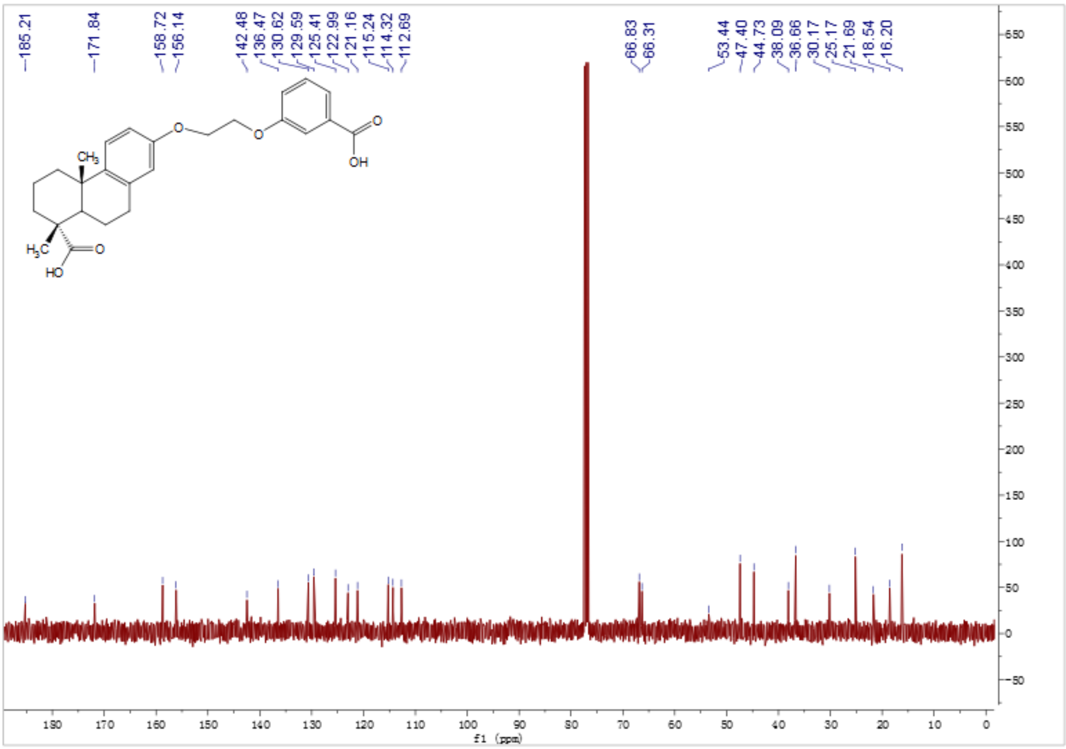

^{13}C NMR (300 MHz, $CDCl_3$) spectrum of compound 22

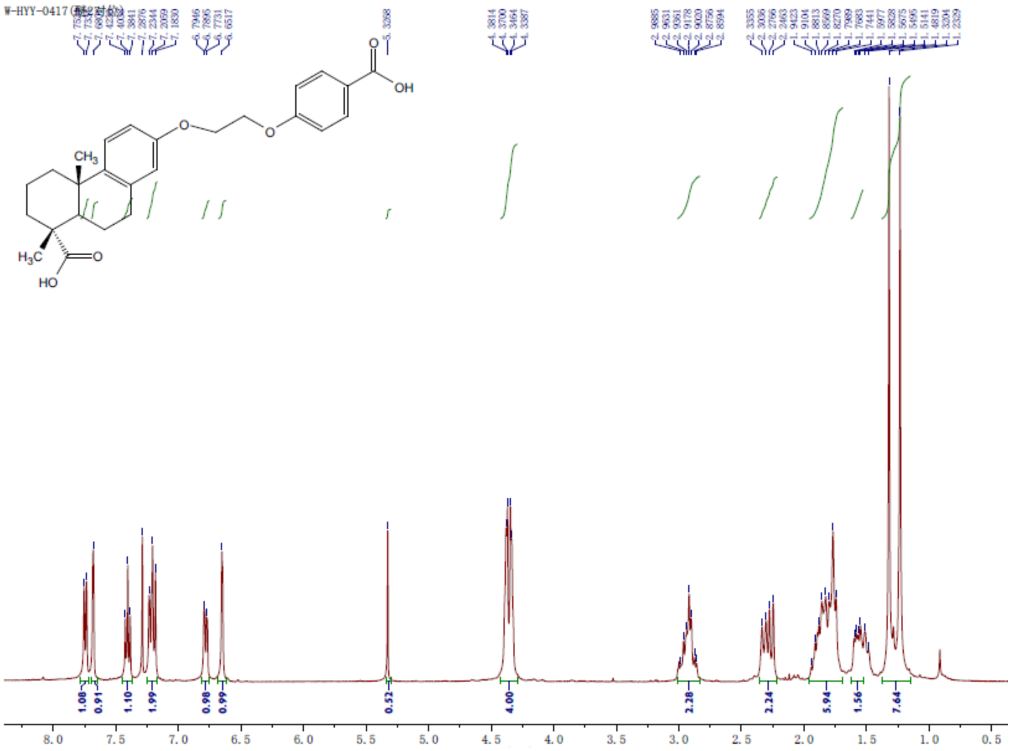

^{1}H NMR (400 MHz, $CDCl_3$) spectrum of compound 23

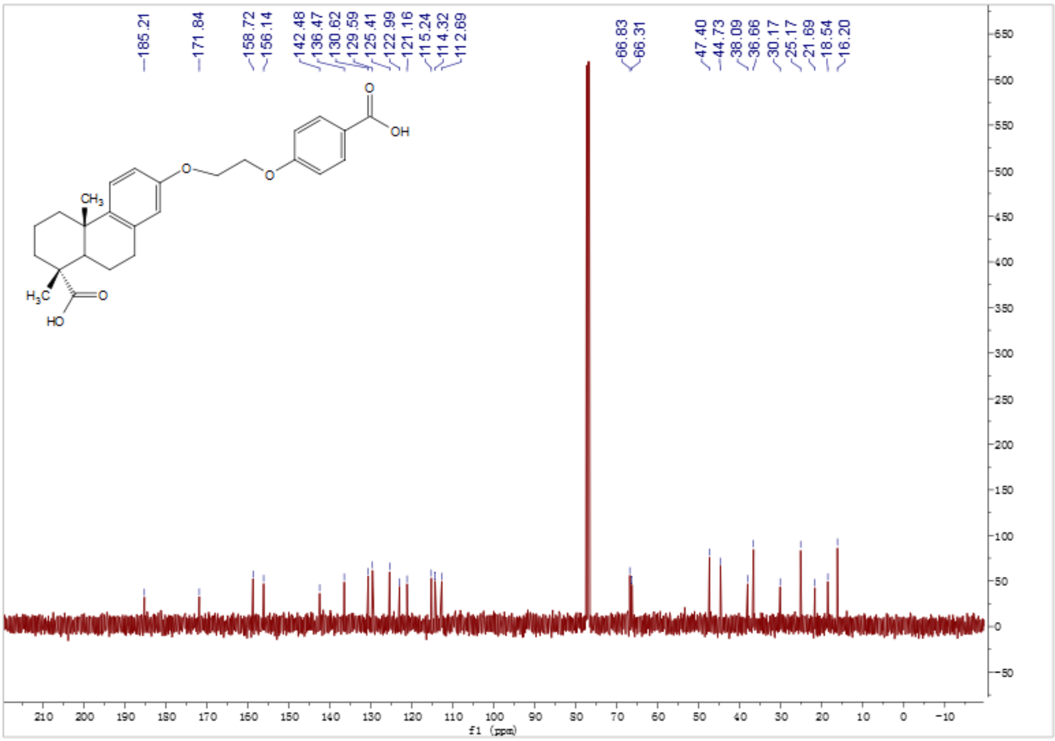

^{13}C NMR (300 MHz, $CDCl_3$) spectrum of compound 23

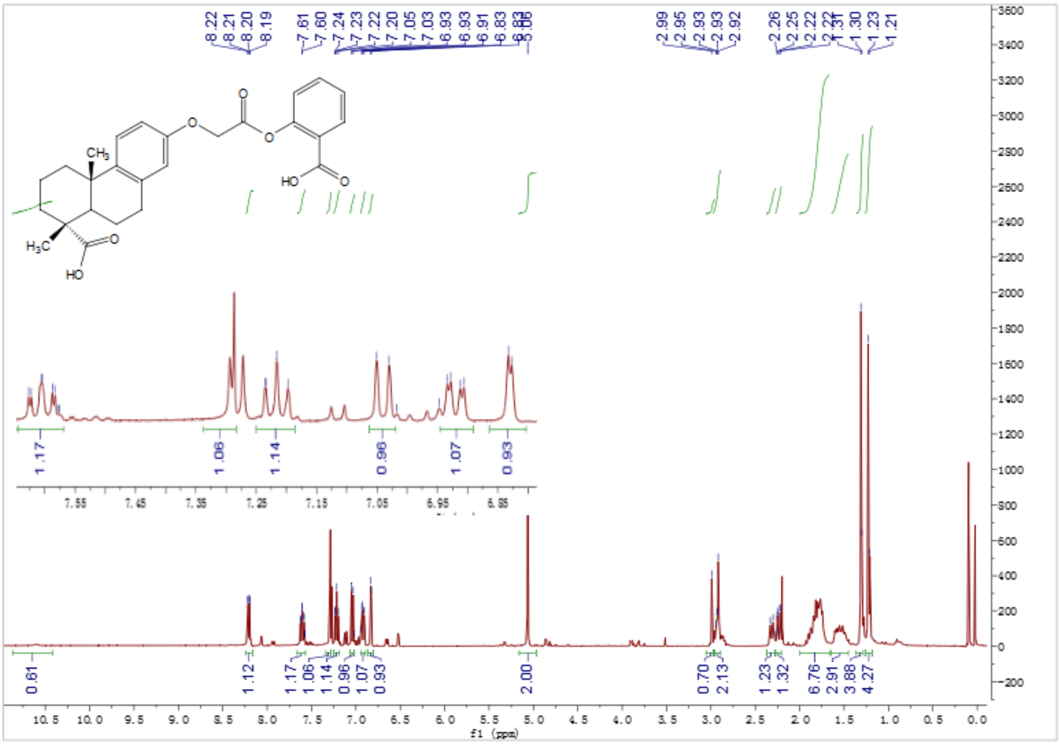

^{1}H NMR (400 MHz, $CDCl_3$) spectrum of compound 24

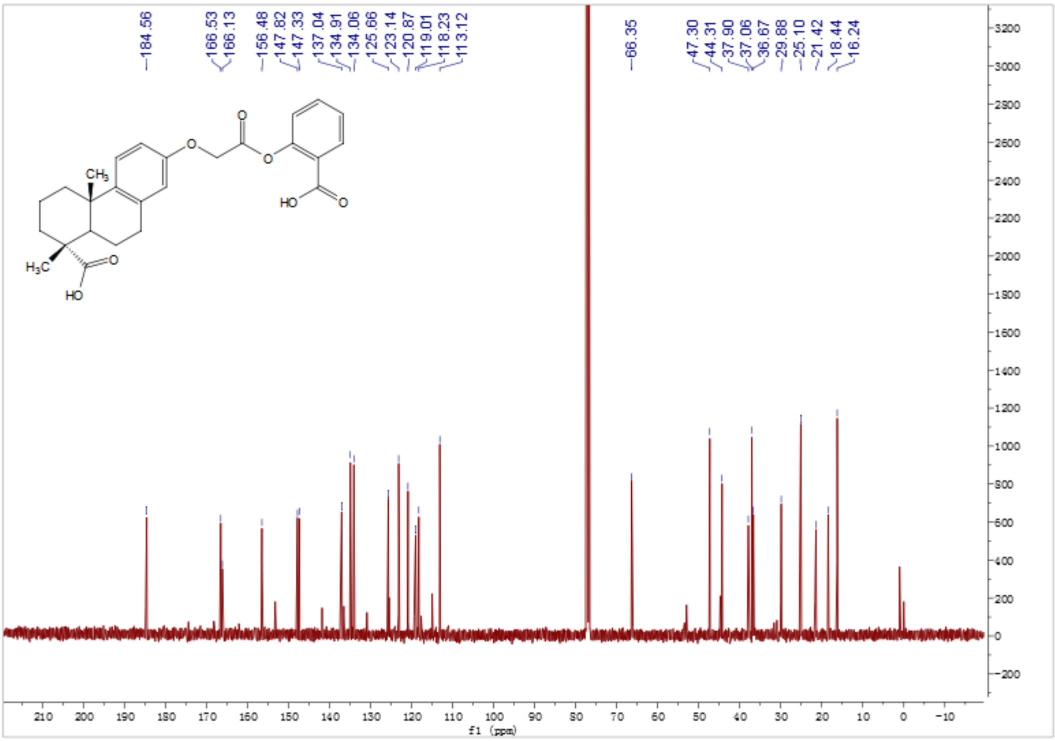

^{13}C NMR (300 MHz, $CDCl_3$) spectrum of compound 24

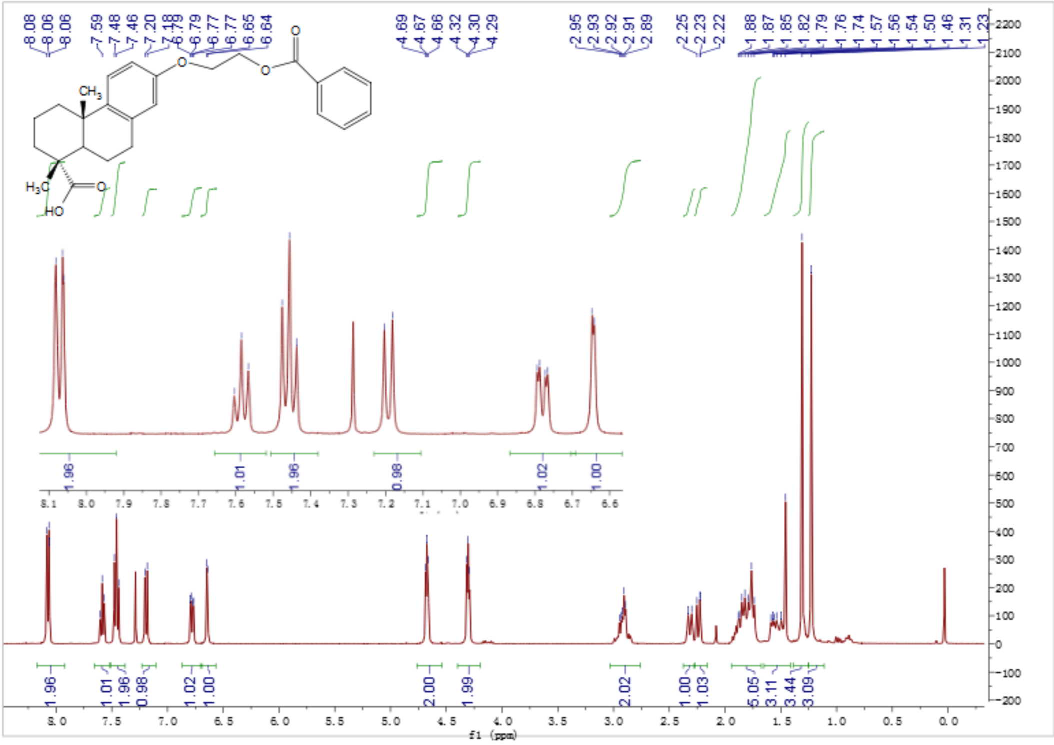

^{1}H NMR (400 MHz, $CDCl_3$) spectrum of compound 25

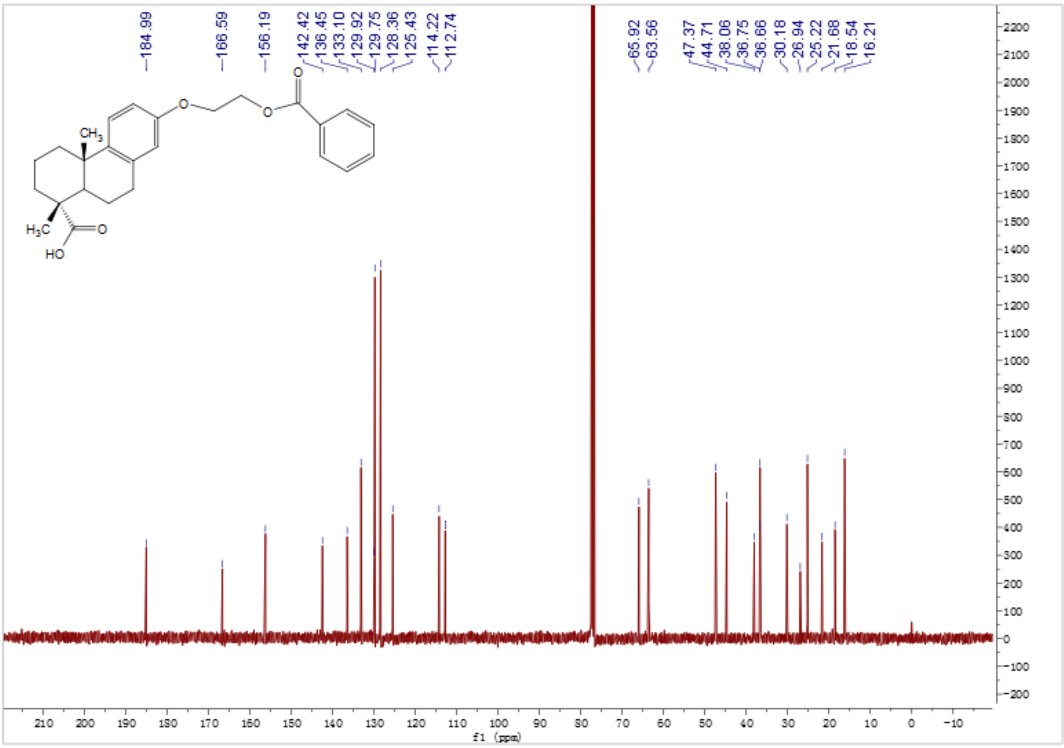

^{13}C NMR (300 MHz, $CDCl_3$) spectrum of compound 25

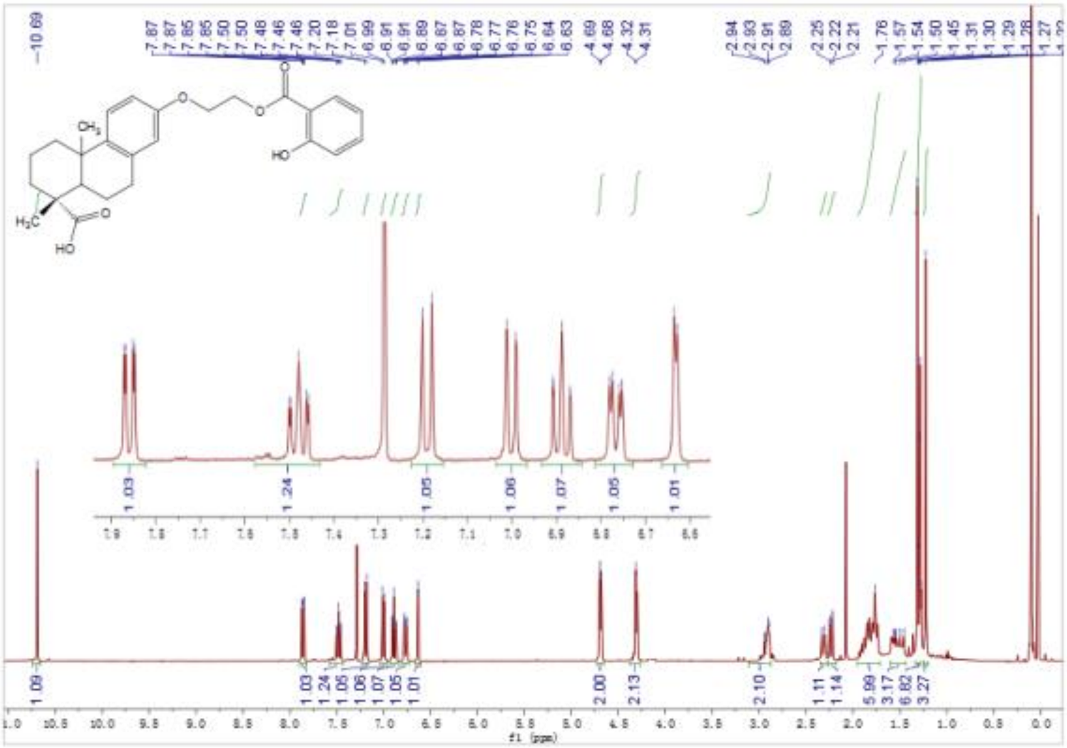

^{1}H NMR (400 MHz, $CDCl_3$) spectrum of compound 26

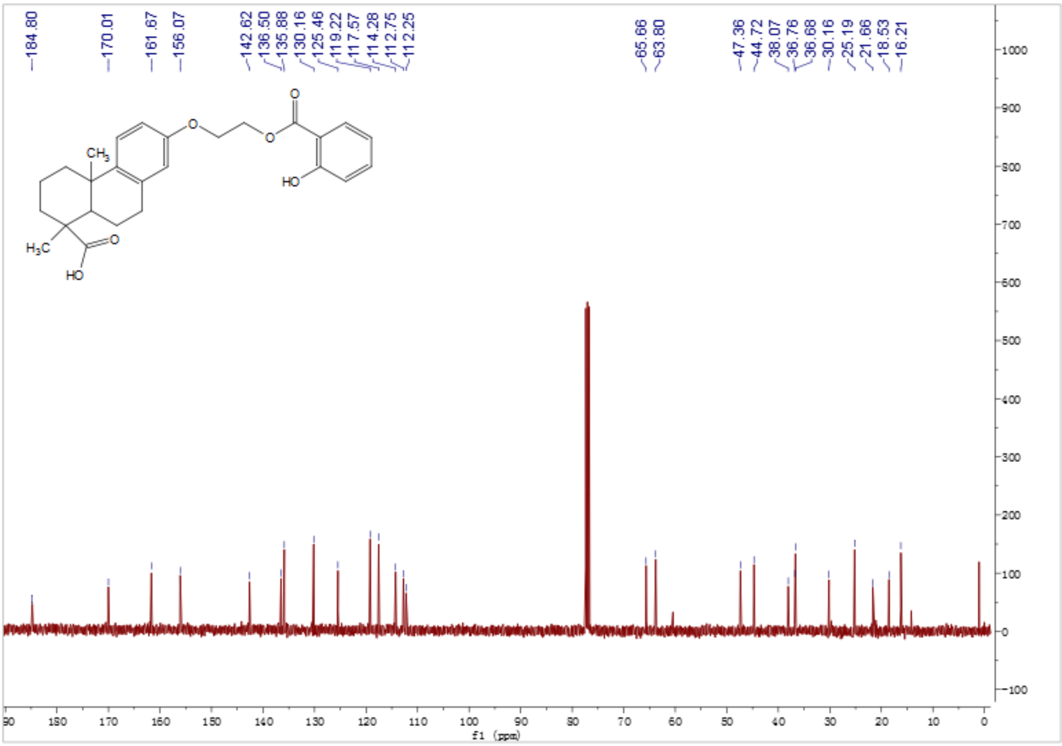

^{13}C NMR (300 MHz, $CDCl_3$) spectrum of compound 26

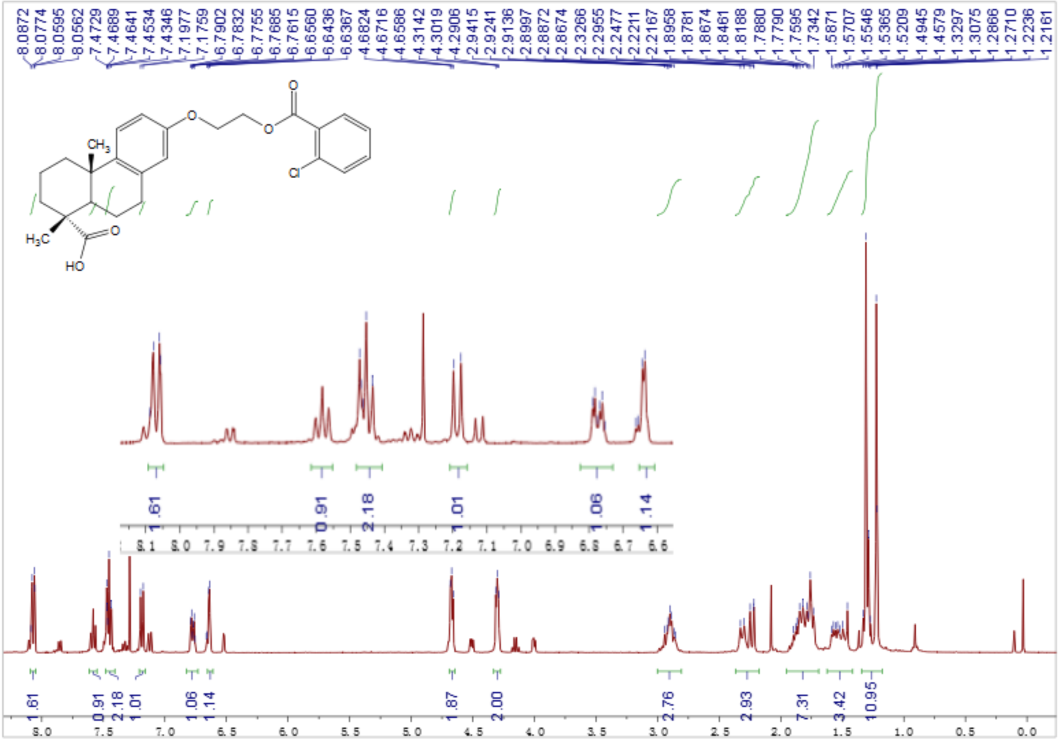

^{1}H NMR (400 MHz, $CDCl_3$) spectrum of compound 27

^{13}C NMR (300 MHz, $CDCl_3$) spectrum of compound 27

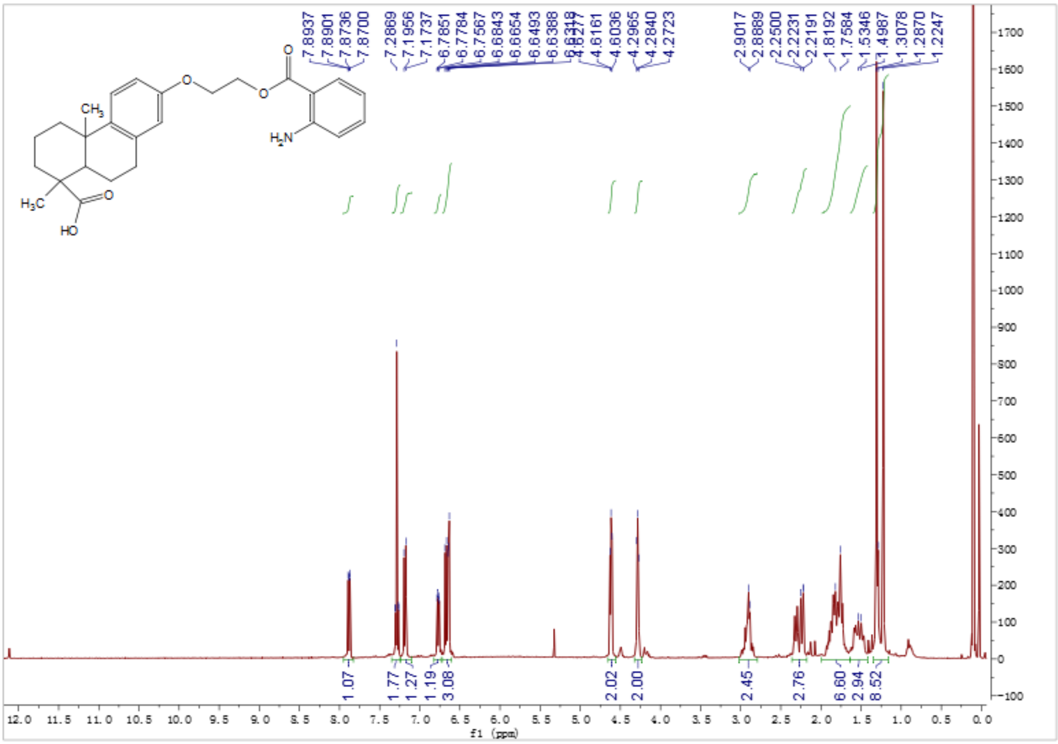

^{1}H NMR (400 MHz, $CDCl_3$) spectrum of compound 28

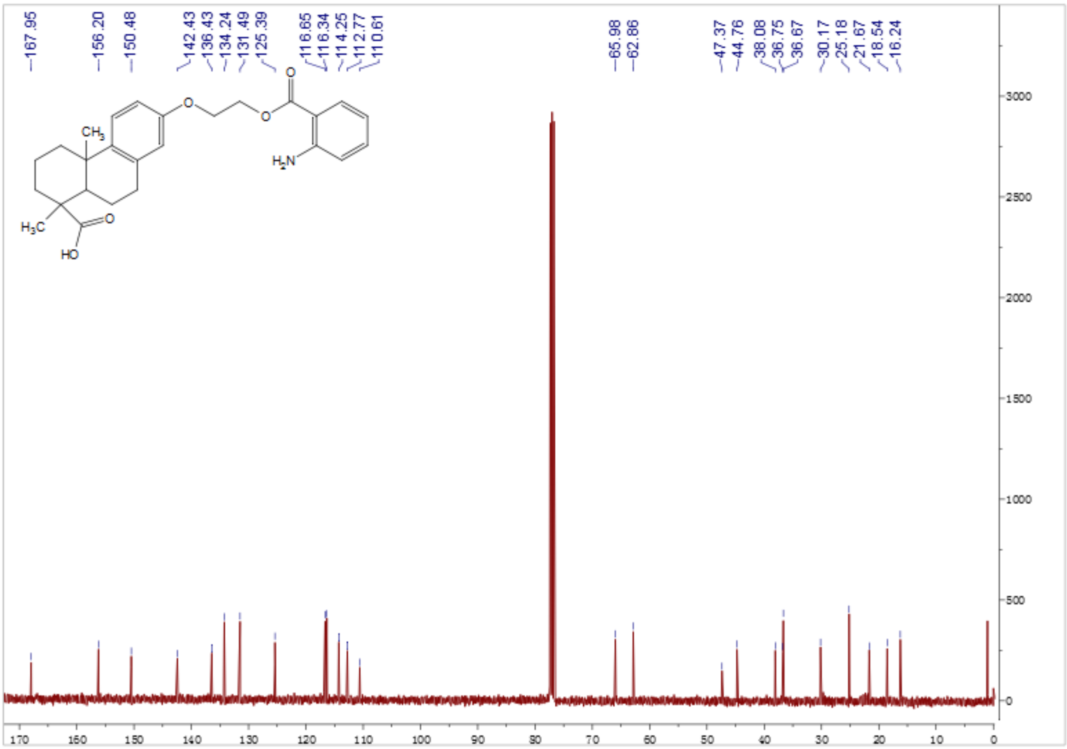

^{13}C NMR (300 MHz, $CDCl_3$) spectrum of compound 28

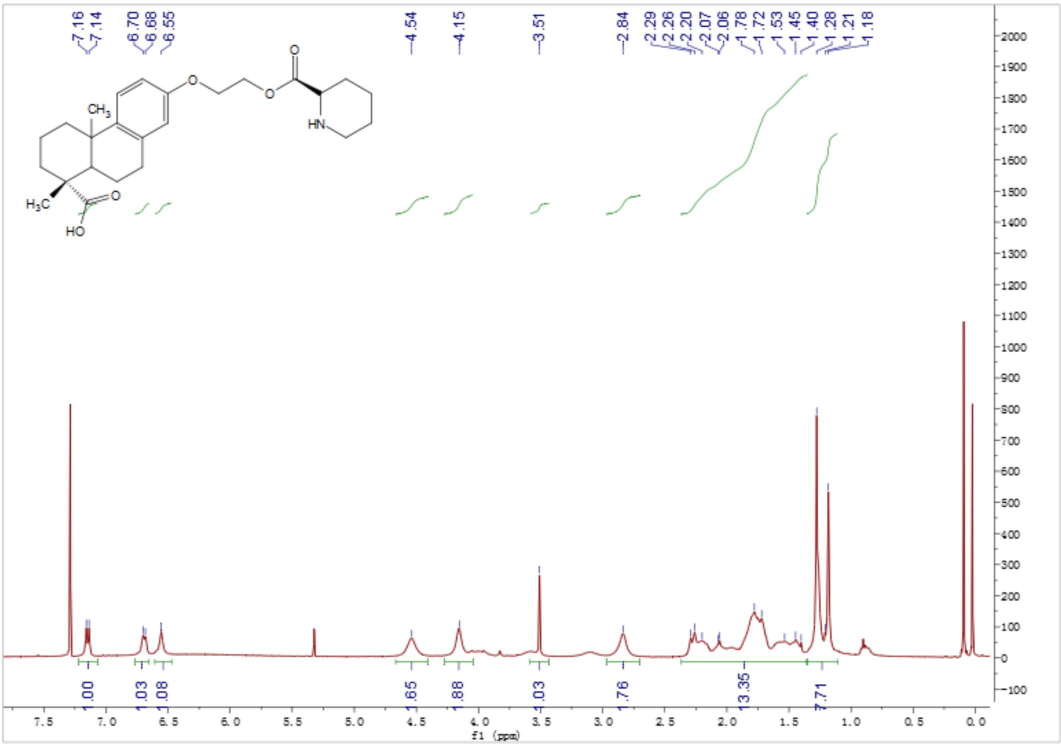

^{1}H NMR (400 MHz, $CDCl_3$) spectrum of compound 29

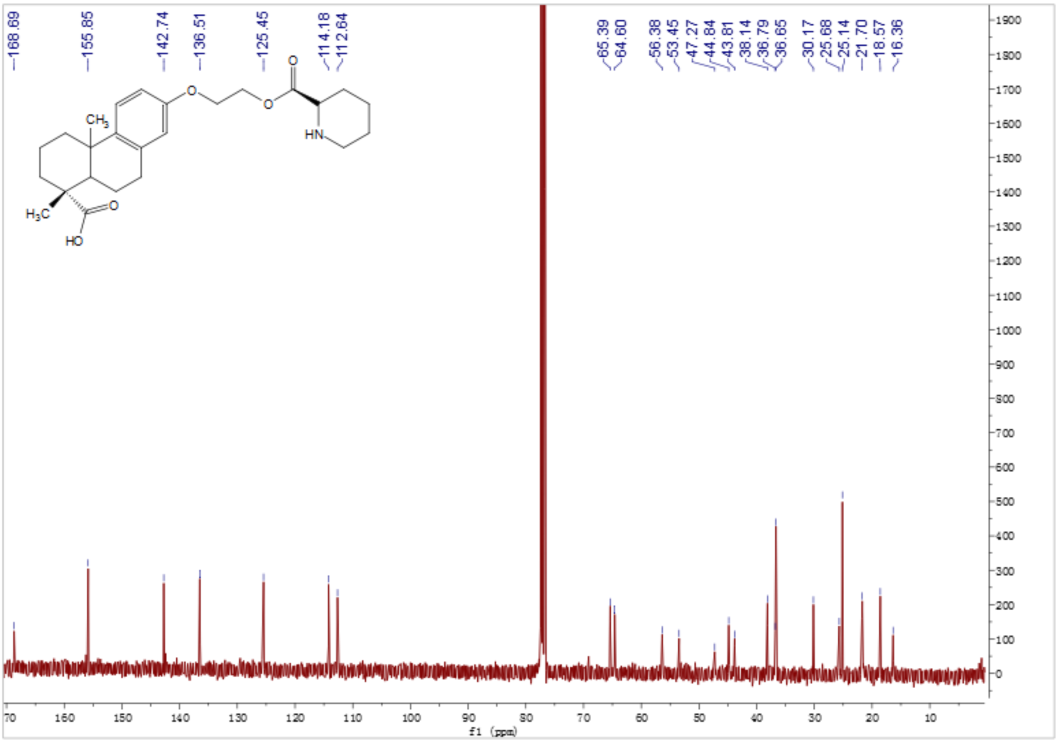

^{13}C NMR (300 MHz, $CDCl_3$) spectrum of compound 29

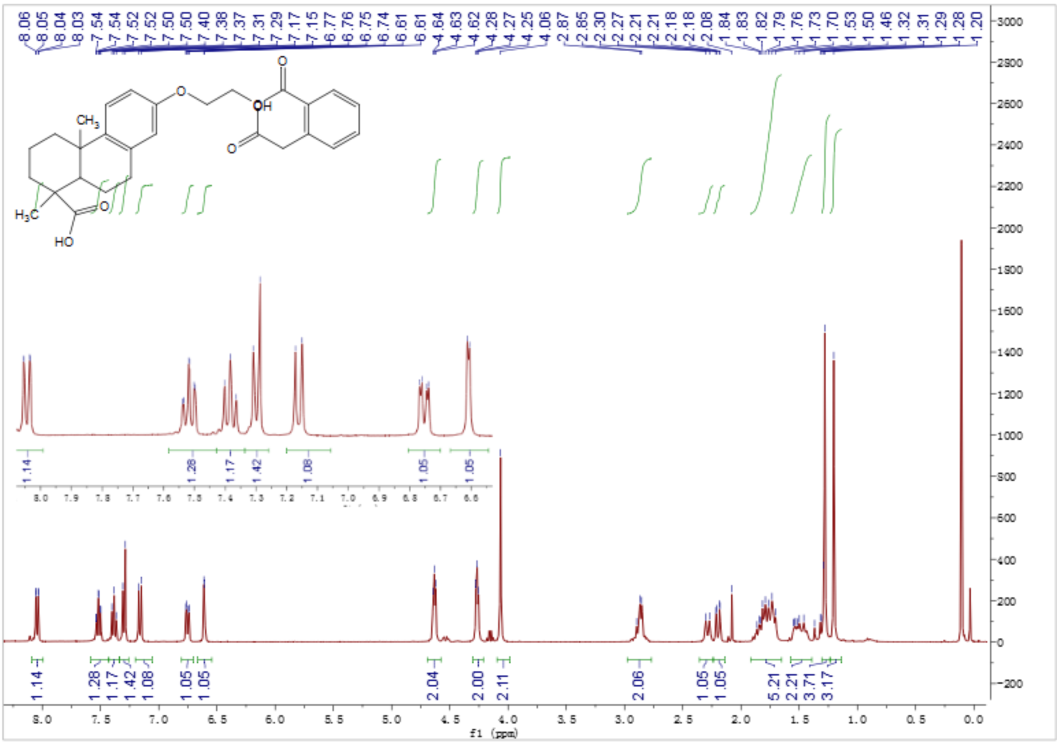

^{1}H NMR (400 MHz, $CDCl_3$) spectrum of compound 30

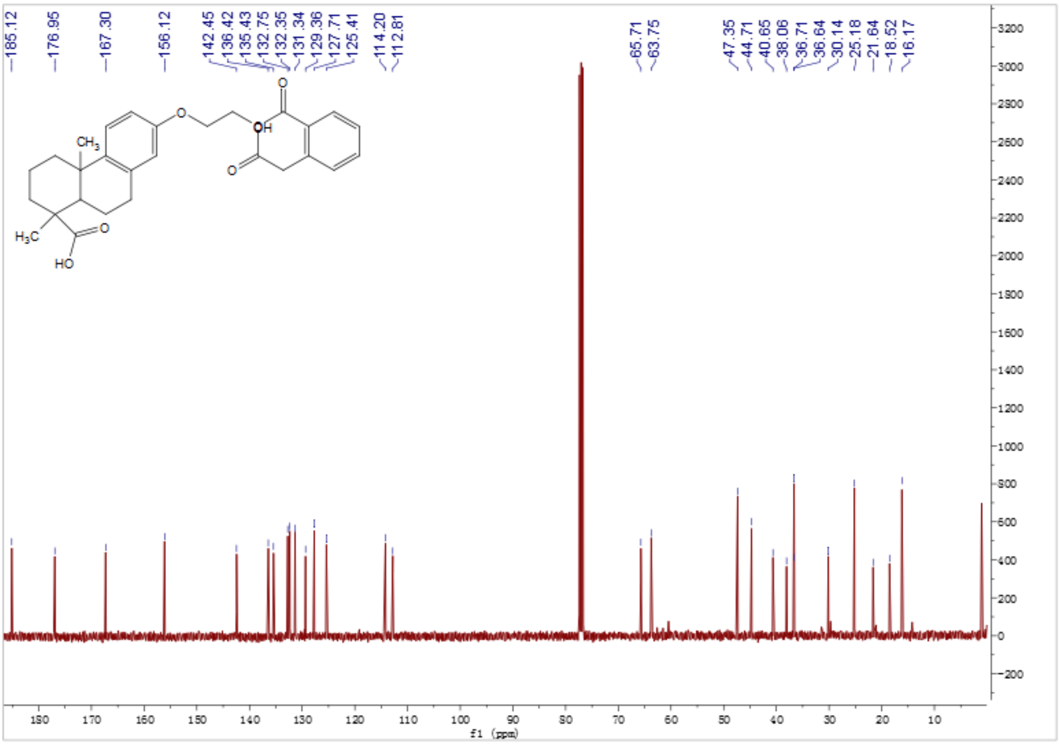

^{13}C NMR (300 MHz, $CDCl_3$) spectrum of compound 30

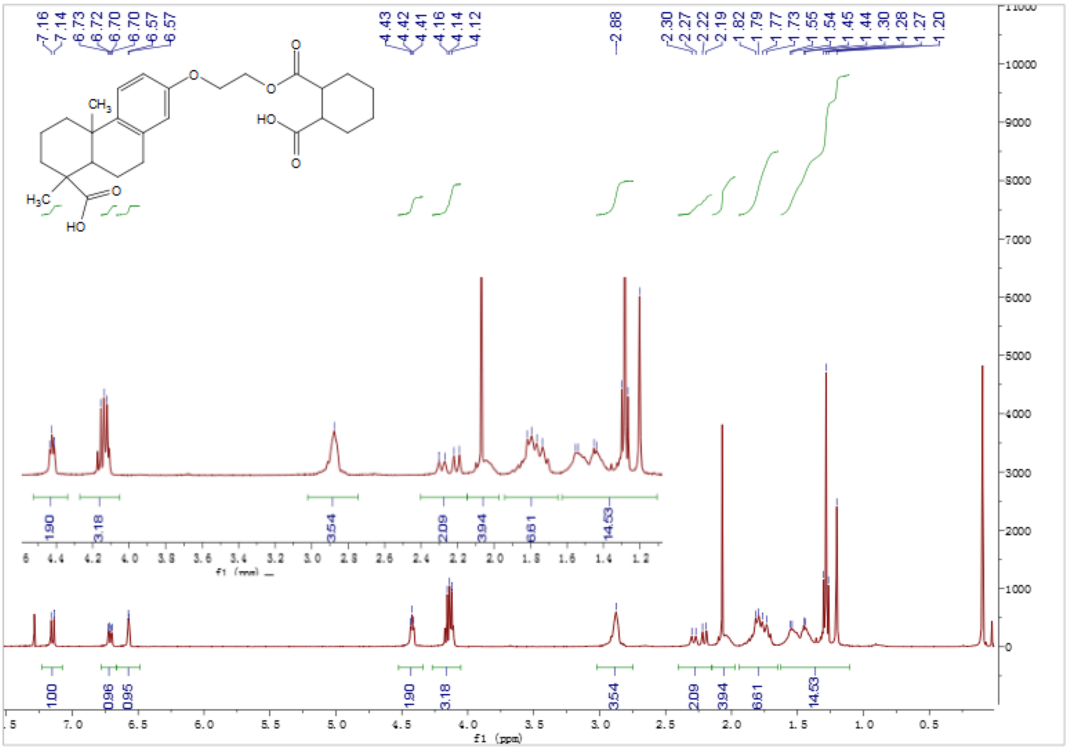

^{1}H NMR (400 MHz, $CDCl_3$) spectrum of compound 31

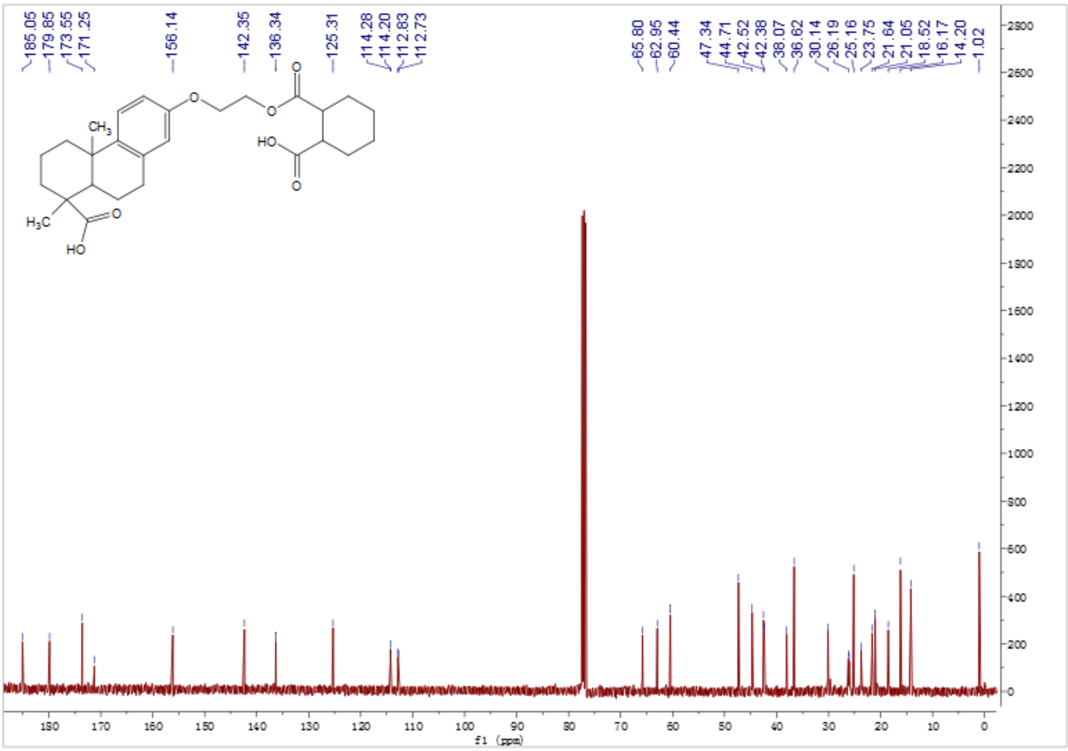

^{13}C NMR (300 MHz, $CDCl_3$) spectrum of compound 31

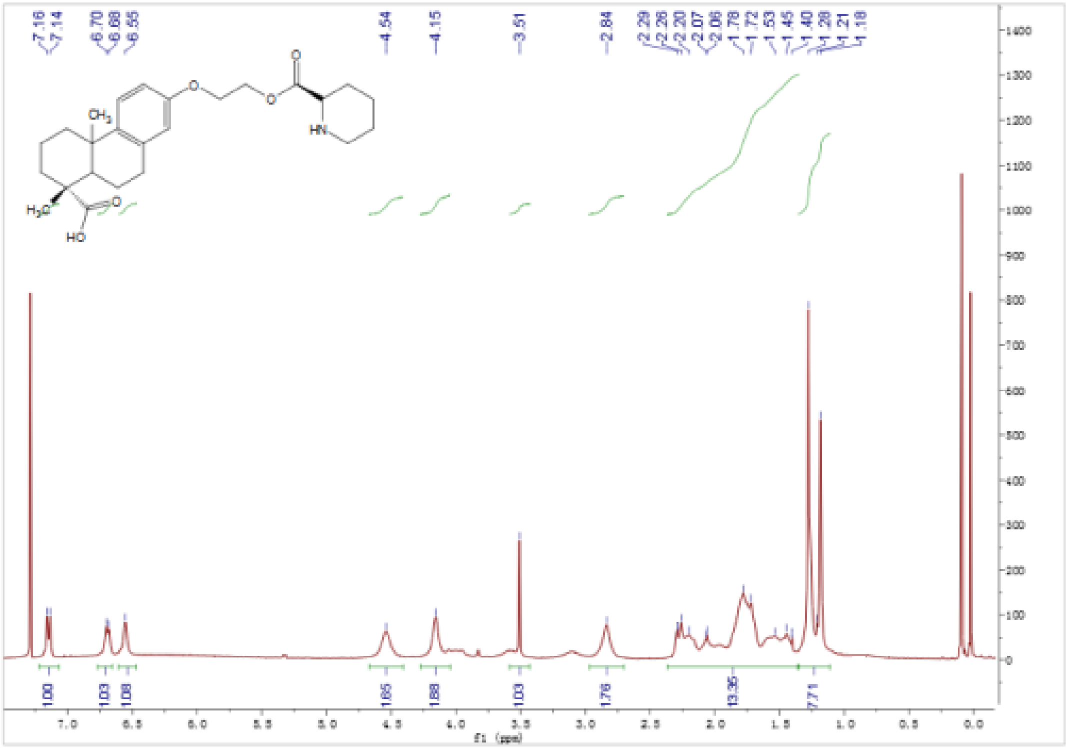

^{1}H NMR (400 MHz, $CDCl_3$) spectrum of compound 32

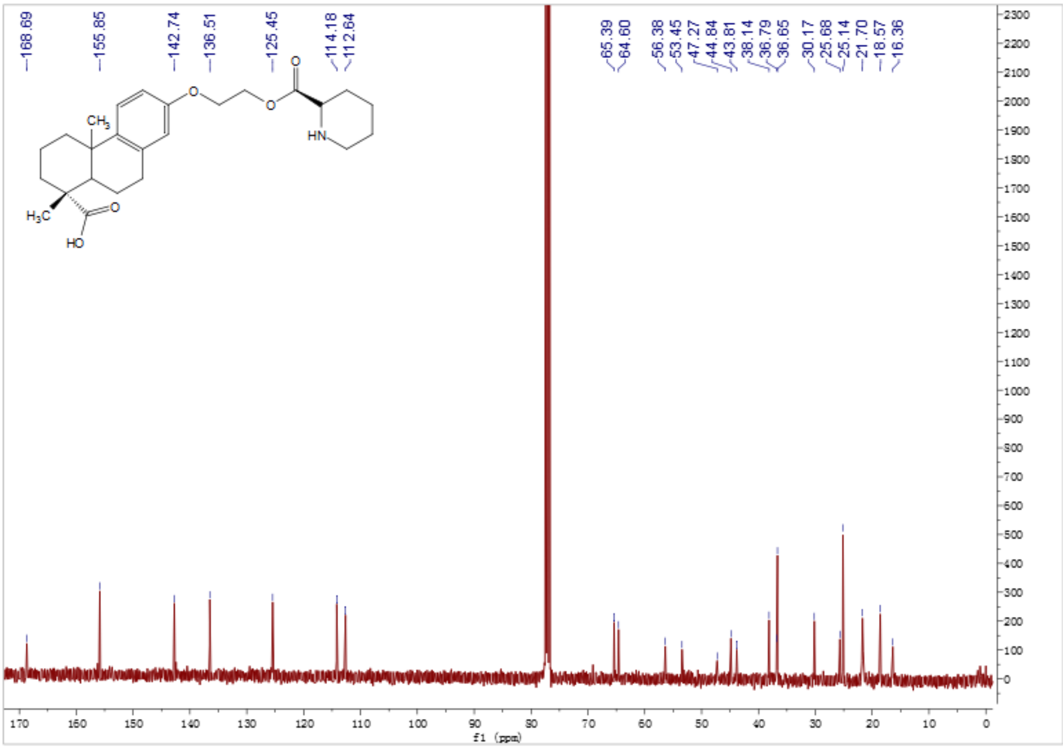

^{13}C NMR (300 MHz, $CDCl_3$) spectrum of compound 32

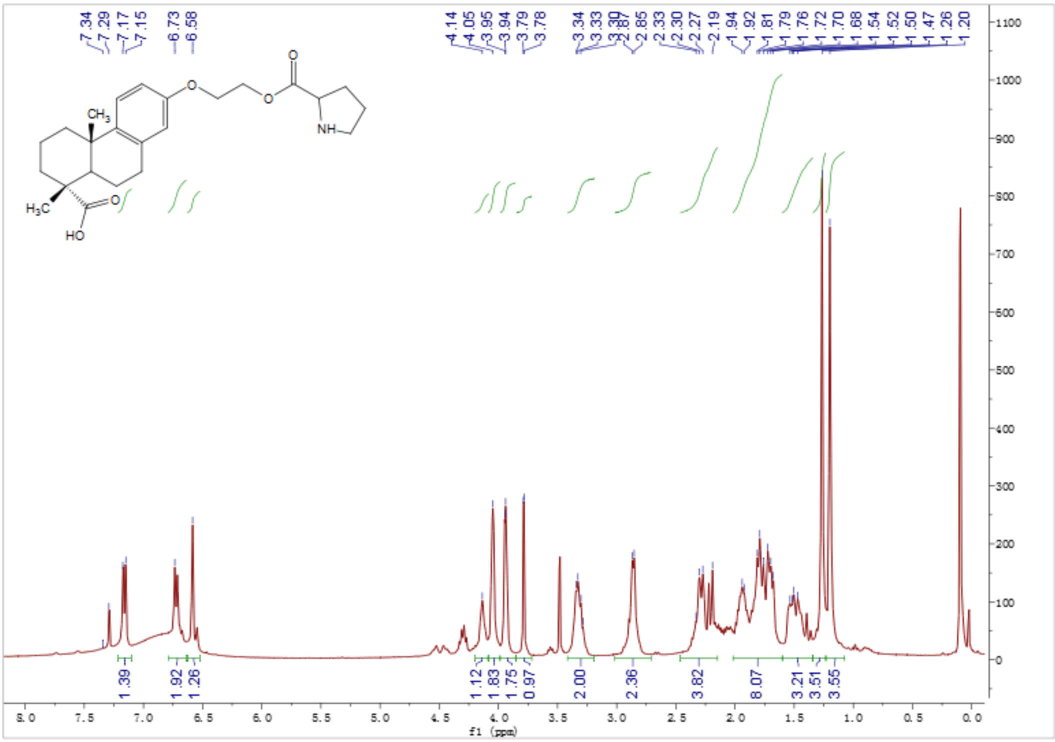

^{1}H NMR (400 MHz, CDCl3) spectrum of compound 33

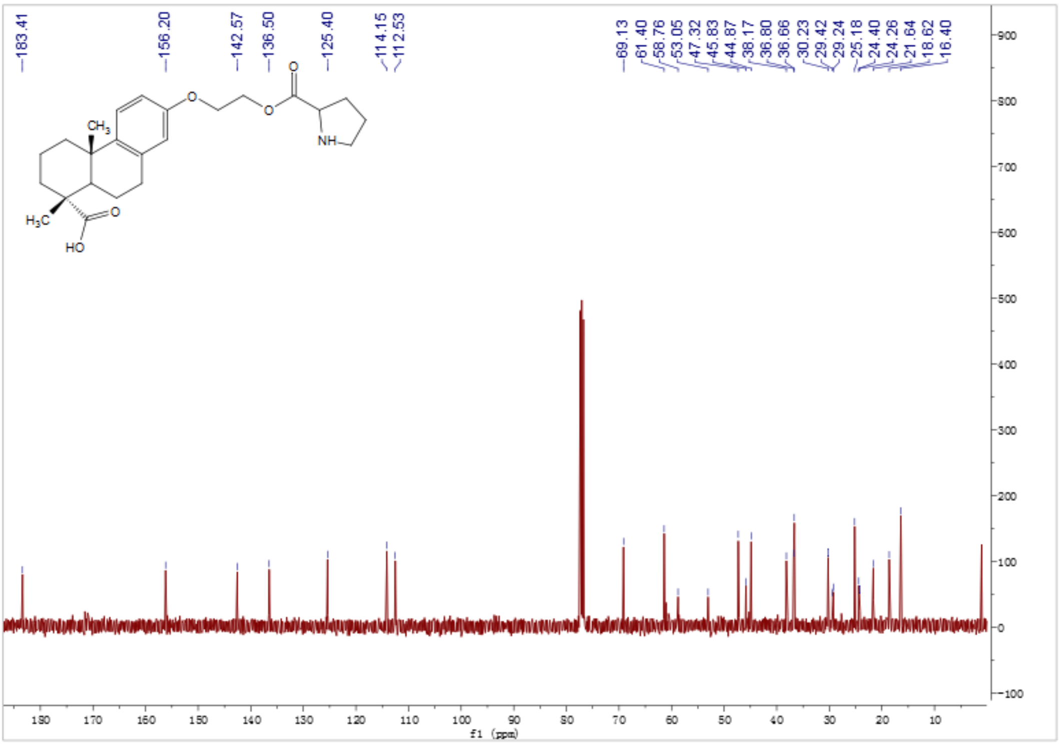

^{13}C NMR (300 MHz, $CDCl_3$) spectrum of compound 33

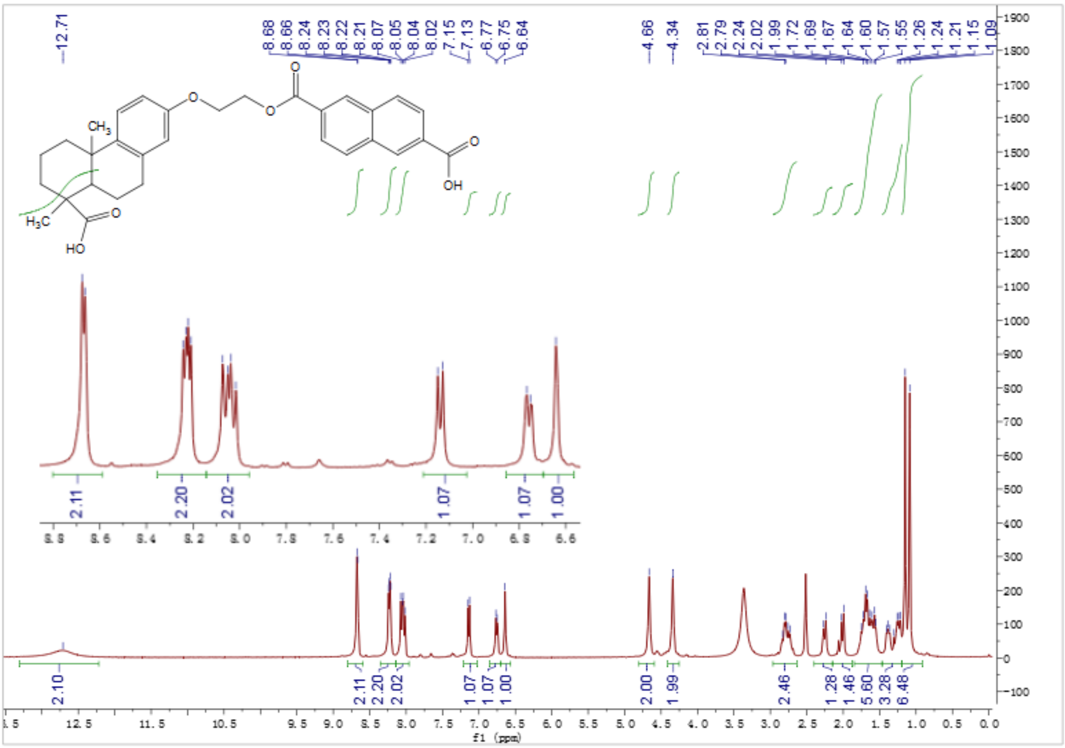

^{1}H NMR (400 MHz, d_6-DMSO) spectrum of compound 34

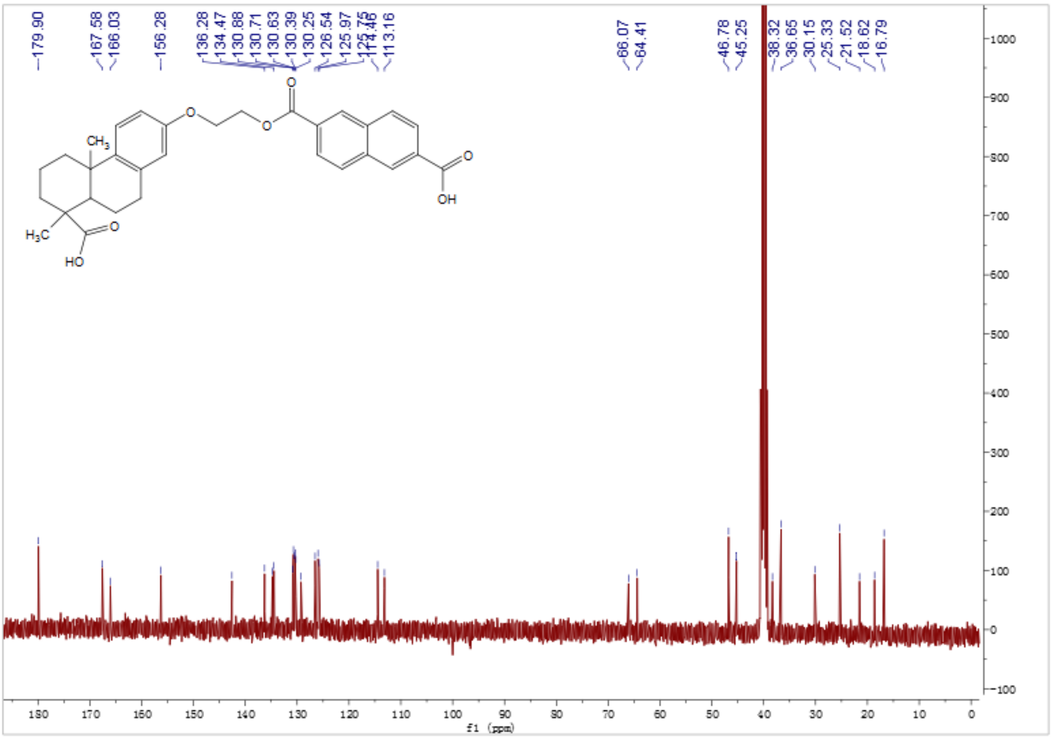

^{13}C NMR (300 MHz, d_6-DMSO) spectrum of compound 34
